# Supplementary material for: Repurposed ethoxzolamide reprograms antitumor immunity through β-TrCP-dependent PD-L1 ubiquitination
Source: Cell Rep Med. 2026 Jul 13;7(7):102920. doi: 10.1016/j.xcrm.2026.102920 (PMC13400190; doi:10.1016/j.xcrm.2026.102920)
Supplement: Document S1. Figures S1–S12 and Tables S1 and S2 [file mmc1.pdf]

Cell Reports Medicine, Volume *7*

Supplemental information

Repurposed ethoxzolamide reprograms antitumor immunity through β-TrCP-dependent PD-L1 ubiquitination

Xuwen Lin, Qun Wang, Mengting Xu, Dianping Yu, Hongmei Hu, Qing Zhang, Jiannan Yao, Mei Xie, Hanchi Xu, Xuefeng Zang, Jia Li, Yu Chen, Linyang Li, Xiaoyu Tao, Xinru Li, Simeng Li, Shize Xie, Yating Tian, Weidong Zhang, Sanhong Liu, and Xinying Xue

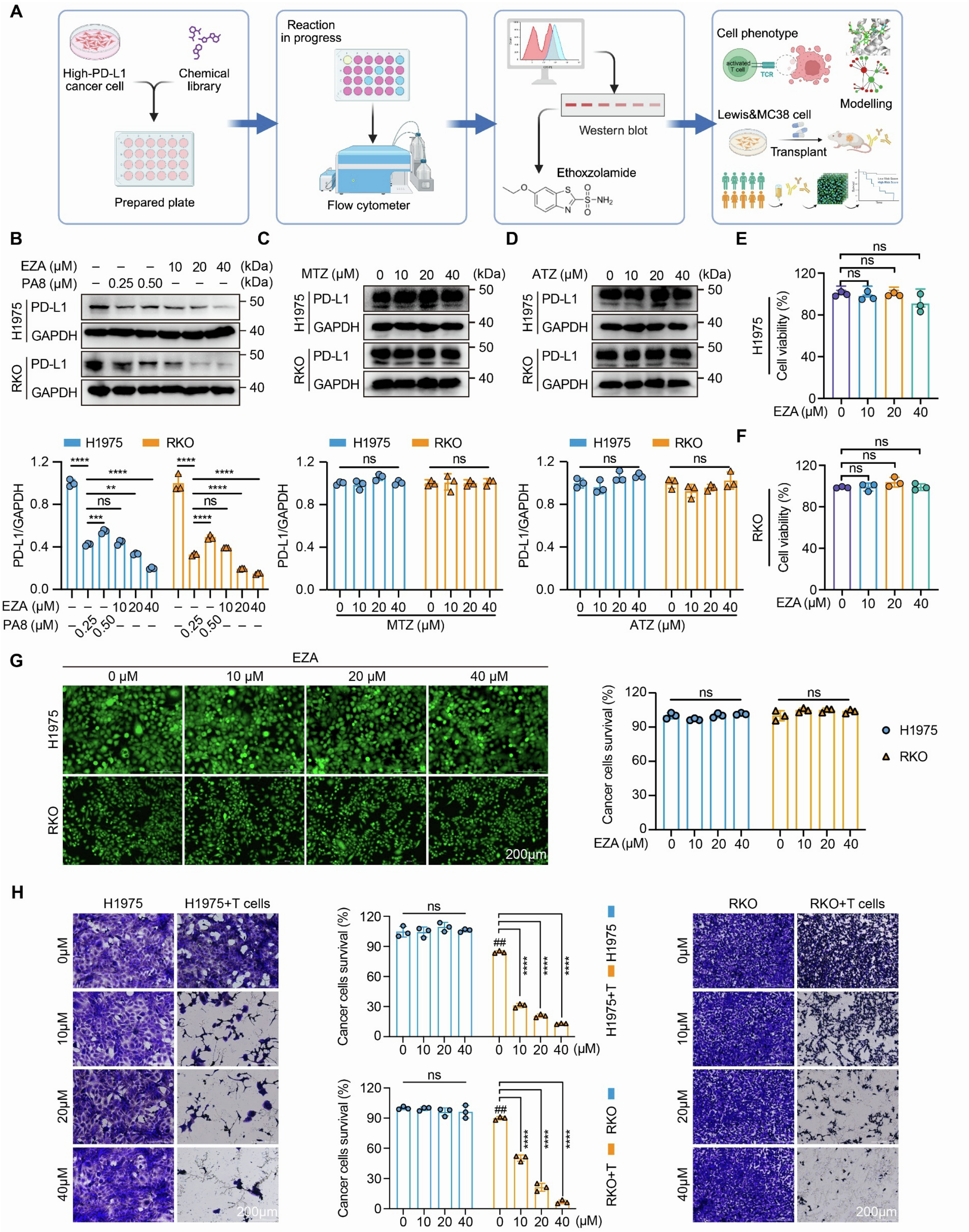

Figure S1. EZA reduces PD-L1 expression without significant cytotoxicity in cancer cells, related to Figure 1.

(A) Workflow for screening a small molecule compound library that reduces PD-L1 expression.

(B) Western blot analysis (top) and quantification (bottom) of PD-L1 protein levels in H1975 and RKO cells treated with the indicated concentrations of EZA for 24 hours. PA8, a known PROTAC PD-L1 degrader (0.25, 0.50 μM), was included as a positive control.

(C) Western blot analysis (top) and quantification (bottom) of PD-L1 protein levels in H1975 and RKO cells treated with methazolamide (MTZ; 0, 10, 20, or 40 μM) for 24 hours.

(D) Western blot analysis (top) and quantification (bottom) of PD-L1 protein levels in H1975 and RKO cells treated with acetazolamide (ATZ; 0, 10, 20, or 40 μM) for 24 hours.

(E-F) Cell viability assessed by CCK-8 assay in H1975 (E) and RKO (F) cells treated with the indicated concentrations of EZA for 24 hours.

(G) The EdU assay was used to quantitatively analyze the ratios of EdU-positive cells to assess the proliferative capacity of H1975 and RKO cells after 24 hours of treatment with various concentrations of EZA.

(H) H1975 and RKO cells were pretreated with the indicated concentrations of EZA for 24 hours and then cocultured with activated primary human T cells for 24 hours. Residual adherent tumor cells were stained with crystal violet and quantified. #P < 0.05 and ##P < 0.01 versus the corresponding tumor cell-only control (H1975 Control or RKO Control); **P < 0.01, ****P < 0.0001 versus the corresponding untreated coculture group (H1975 + T/Jurkat cells or RKO + T/Jurkat cells). Data are presented as mean ± SD from three independent experiments. Statistical significance was determined by one-way ANOVA (E-F) or two-way ANOVA (B, C, G, and H). *$P < 0.05$, **$P < 0.01$, ***$P < 0.001$, ****$P < 0.0001$; ns, not significant.

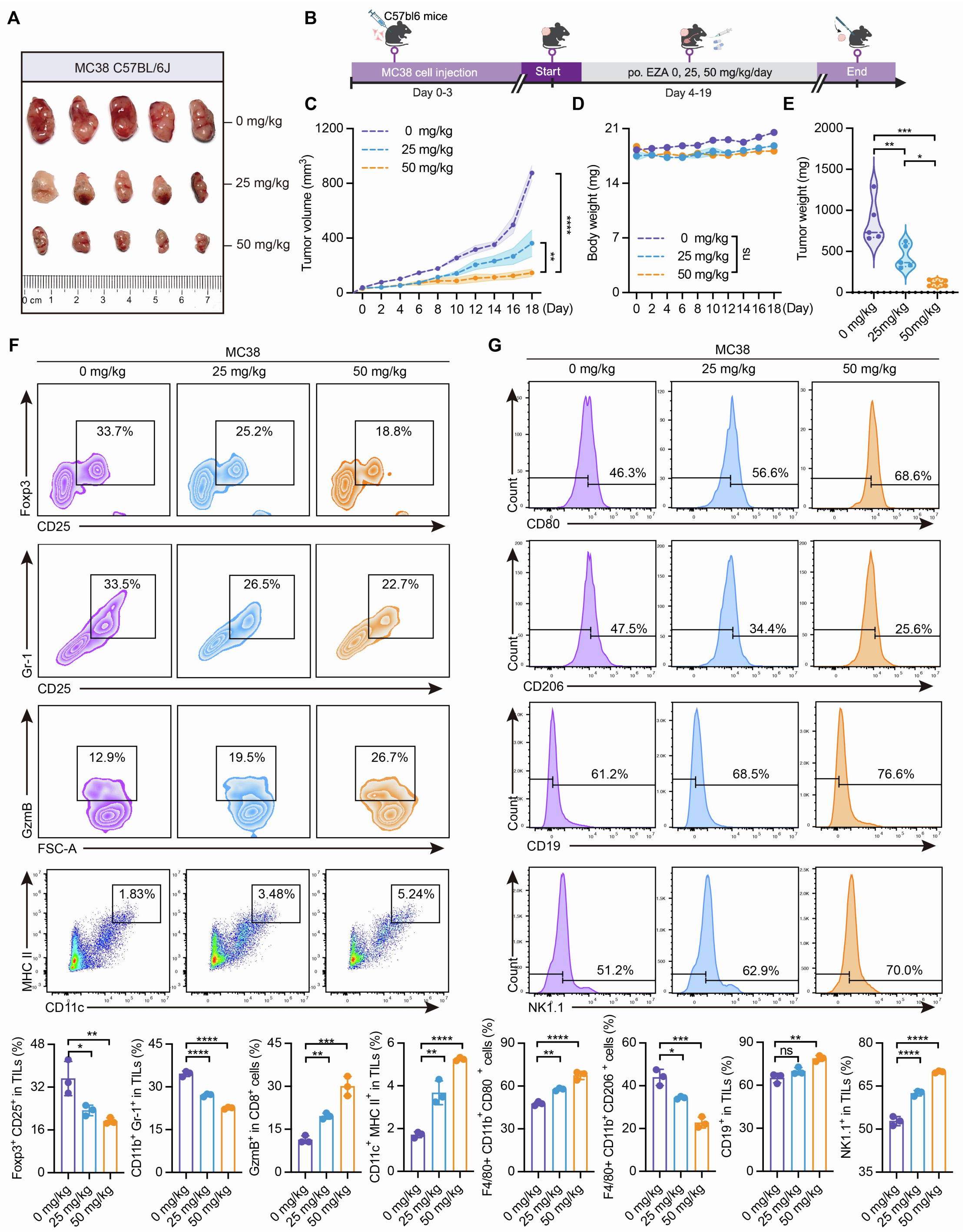

Figure S2. EZA suppresses subcutaneous MC38 colorectal tumor growth by modulating the immune microenvironment, related to Figure 2.

(A) Representative images of subcutaneous MC38 tumors from female C57BL/6J mice treated with vehicle or EZA (25 or 50 mg/kg) for the duration indicated in (B) (n = 5 mice per group).

(B) Schematic of the experimental timeline for the MC38 subcutaneous tumor model and drug administration regimen.

(C) Tumor growth curves of mice during the treatment period.

(D) Body weight changes of mice throughout the experimental timeline.

(E) Final tumor weights at the experimental endpoint.

(F-G) Flow cytometry and quantitative analysis of immune cell infiltration in the tumor microenvironment, including Tregs ($CD4^+CD25^+Foxp3^+$), Granzyme B^+ cytotoxic lymphocytes, MDSCs ($CD11b^+Gr\text{-}1^+$), mature DCs ($CD11c^+MHC\text{-}II^+$), M1 macrophages ($F4/80^+CD11b^+CD80^+$), M2 macrophages ($F4/80^+CD11b^+CD206^+$), and NK cells ($NK1.1^+$). Data in (C-G) are presented as mean ± SD. Statistical significance was determined by one-way ANOVA (E-G) or two-way ANOVA (C, D). *$P < 0.05$, **$P < 0.01$, ***$P < 0.001$, ****$P < 0.0001$; ns, not significant.

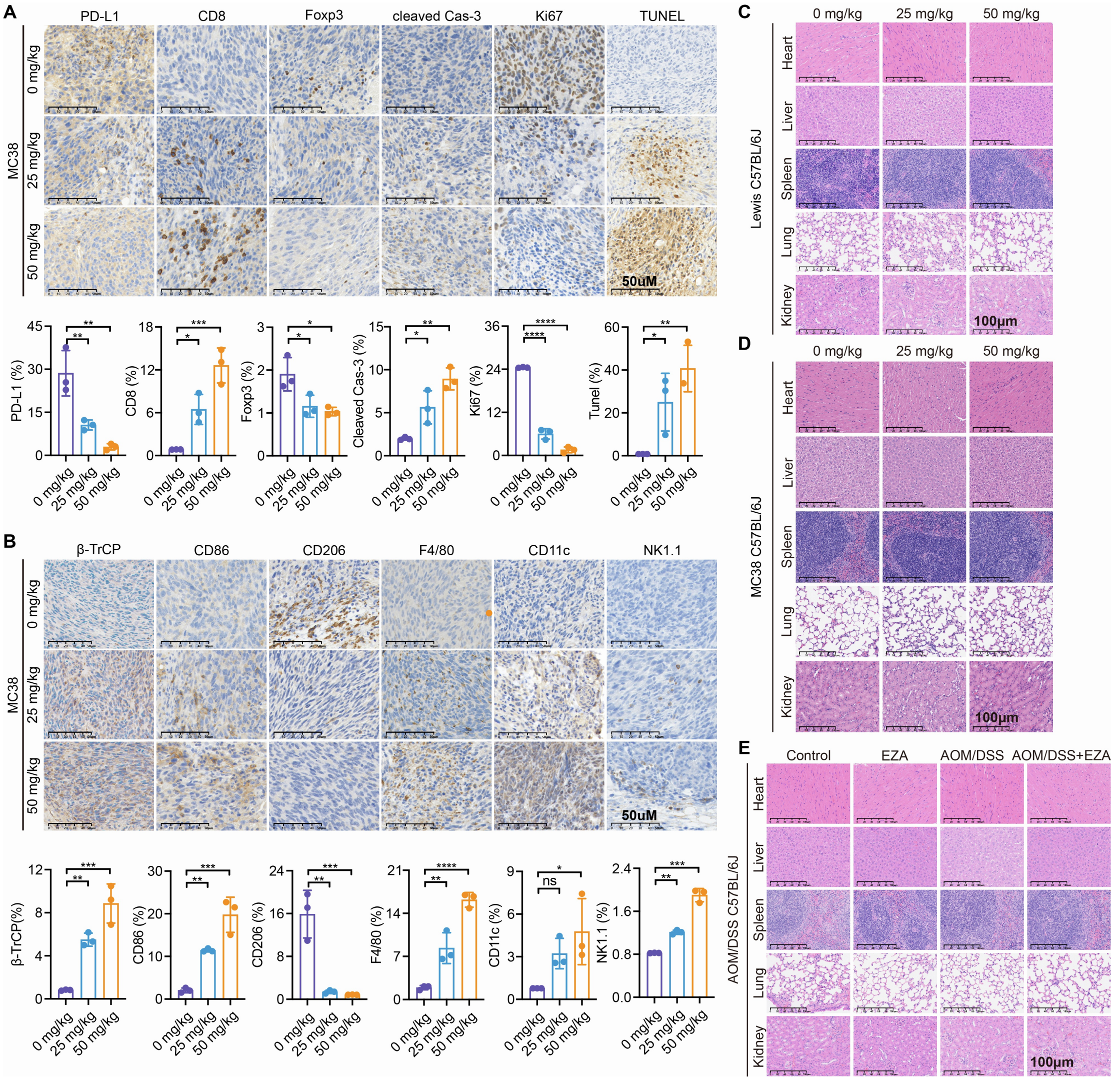

Figure S3. Analysis of tumor microenvironment remodeling and systemic safety following EZA treatment in multiple in vivo models, related to Figures 2 and 3.

(A-B) Immunohistochemical profiling of the tumor microenvironment in MC38 subcutaneous tumors. Representative images of immunohistochemical staining and quantitative analysis of tumor tissues following EZA treatment. The evaluated parameters included PD-L1, CD8, Foxp3, Caspase-3, TUNEL, Ki-67, CD86, F4/80, CD206, CD11c, and NK1.1. Scale bar, 50 μm.

(C-E) Histopathological assessment of major organ safety across multiple tumor models. Representative hematoxylin and eosin-stained sections of major organs (heart, liver, spleen, lung, and kidney) harvested from tumor-bearing mice at the experimental endpoint. (C) Subcutaneous Lewis lung carcinoma (LLC) model treated with vehicle or EZA (25 or 50 mg/kg). (D) Subcutaneous MC38 colorectal tumor model treated with vehicle or EZA (25 or 50 mg/kg). (E) AOM/DSS-induced colitis-associated colorectal cancer model treated with vehicle or EZA (50 mg/kg). No significant treatment-related pathological alterations were observed. Scale bar, 100 μm. Data are presented as mean ± SD from three independent experiments. Statistical significance was determined by one-way ANOVA (A-B)**.** Statistical significance was determined by one-way ANOVA (A-B). *$P < 0.05$, **$P < 0.01$, ***$P < 0.001$, ****$P < 0.0001$; ns, not significant.

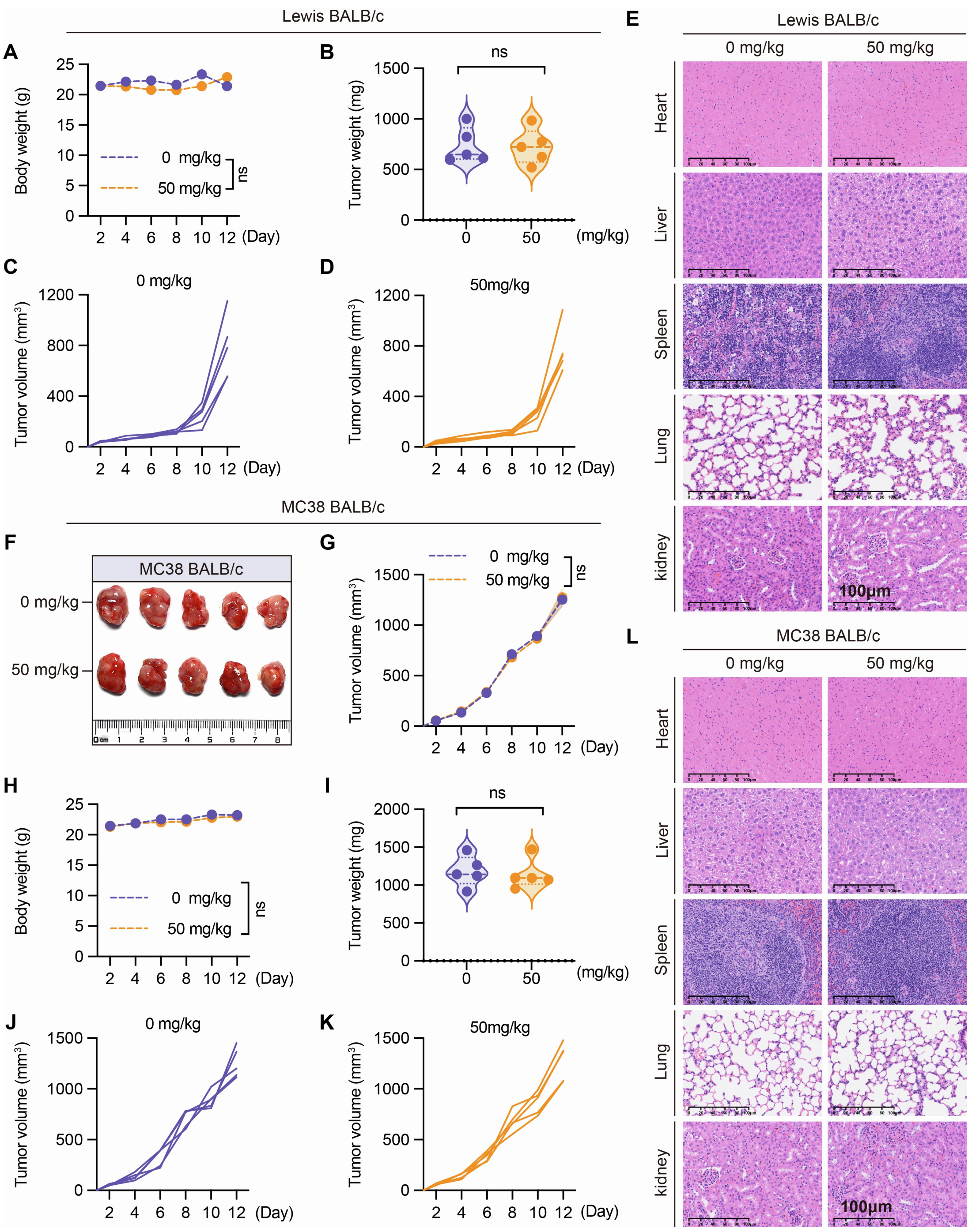

Figure S4. EZA does not inhibit lung or colorectal cancer growth in immunodeficient mouse models, related to Figures 2 and 6.

(A-D) Analysis of the LLC subcutaneous tumor model in nude mice. (A) Body weight changes of mice during the treatment period. (B) Final tumor weights at the experimental endpoint. (C-D) Individual tumor growth curves for mice treated with vehicle (C) or 50 mg/kg EZA (D).

(E) Representative H&E image of major organs from LLC-bearing nude mice at the endpoint. Scale bar, 100 μm.

(F) Representative images of excised MC38 subcutaneous tumors from nude mice treated with vehicle or 50 mg/kg EZA (n = 5 per group).

(G-I) Quantitative analysis of the MC38 model in nude mice. (G) Tumor growth curves. (H) Body weight

changes during treatment. (I) Final tumor weights.

(J-K) Individual tumor growth curves for MC38-bearing nude mice treated with vehicle (J) or 50 mg/kg EZA (K).

(L) Representative H&E-stained sections of major organs from MC38-bearing nude mice at the endpoint. Scale bar, 100 μm. Data in (A-B and G-J) are presented as mean ± SD (n = 5 mice per group). Statistical significance was determined by one-way ANOVA (B and I) or two-way ANOVA (A, G, and H). ns, not significant.

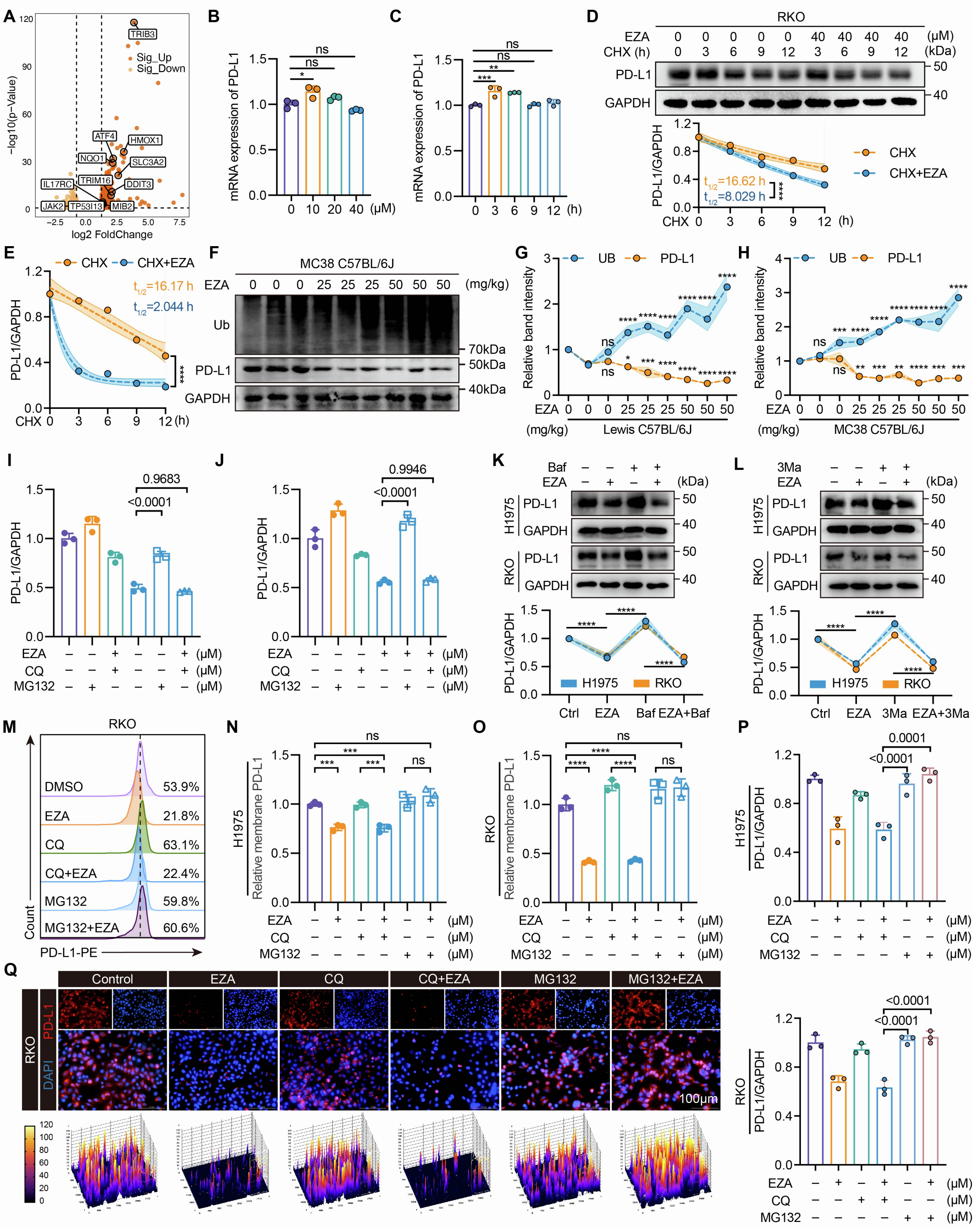

Figure S5. EZA promotes PD-L1 degradation via the ubiquitin-proteasome pathway, related to Figure 4.

(A) Volcano plot of differentially expressed genes (DEGs) in RKO cells treated with 40 µM EZA versus DMSO for 24 hours (|$\log_2$ fold change| > 1, P < 0.05). Upregulated genes are shown in red, downregulated genes in blue.

(B-C) RT qPCR analysis of PD-L1 mRNA levels in RKO cells treated with increasing concentrations of EZA for 24 hours (B) or with 40 µM EZA for the indicated durations (C). Data are normalized to GAPDH.

(D) Western blot analysis of PD-L1 protein levels in RKO cells treated with cycloheximide (CHX, 50 µg/mL) for the indicated times, with or without 40 µM EZA.

(E) Quantification of PD-L1 protein half-life from the data in (D).

(F-G) Analysis of PD-L1 ubiquitination in vivo. (F) Western blot analysis of ubiquitinated PD-L1 in MC38 subcutaneous tumors from mice treated with vehicle or EZA (25 or 50 mg/kg). (G) Corresponding quantitative analysis of the ubiquitination signal.

(H) Quantification of PD-L1 ubiquitination in Lewis lung carcinoma (LLC) subcutaneous tumors from mice treated as indicated (related to Figure 4H).

(I-J) Western blot analysis of PD-L1 protein levels in H1975 (I) and RKO (J) cells treated with EZA (40 μM, 12 h) in the presence or absence of the proteasome inhibitor MG132 (5 μM, 6 h) or the lysosome inhibitor chloroquine (CQ, 40 μM, 6 h).

(K-L) Western blot analysis (top) and quantification (bottom) of PD-L1 levels in H1975 and RKO cells co-treated with EZA and bafilomycin A1 (Baf, 800 nM, 12 h) (K) or 3-methyladenine (3MA, 1 mM, 6 h) (L).

(M) Flow cytometry analysis of cell-surface PD-L1 expression in RKO cells treated as in (I-J).

(N-O) Flow cytometry analysis of cell-surface PD-L1 expression in H1975 (N) and RKO (O) cells under the indicated treatment conditions.

(P) Quantification of the mean fluorescence intensity (MFI) of membrane PD-L1 from immunofluorescence analysis in H1975 cells (related to Figure 4G).

(Q) Representative immunofluorescence (IF) images showing membrane PD-L1 (red) in RKO cells treated as in (M). The 2.5D reconstruction (bottom) quantifies the fluorescence intensity distribution. Scale bar, 100 μm. Data are presented as mean ± SD from three independent experiments. Statistical significance was determined by one-way ANOVA (B, C, I, J, and N-Q) or two-way ANOVA (D, E, G, H, K and L). *$P < 0.05$, **$P < 0.01$, ***$P < 0.001$, ****$P < 0.0001$; ns, not significant.

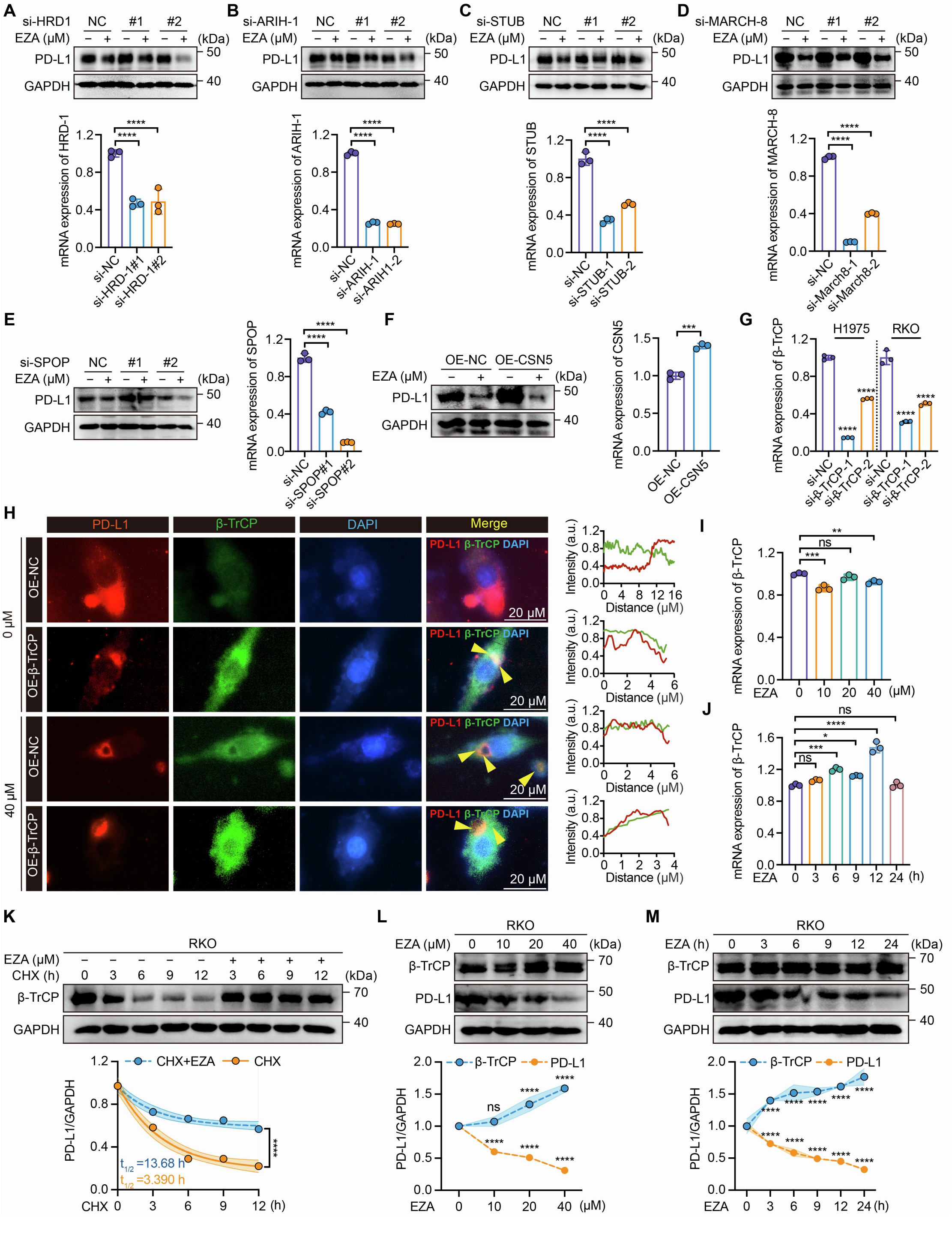

Figure S6. EZA promotes PD-L1 ubiquitination and degradation by targeting β-TrCP, related to Figure 4.

(A-F) Western blot analysis of PD-L1 protein levels in RKO cells treated with 40 μM EZA for 24 hours following knockdown or overexpression of indicated E3 ubiquitin ligases: (A) HRD1, (B) ARIH1, (C) STUB1, (D) MARCH8, (E) SPOP, and (F) CSN5. RT-qPCR confirmed the knockdown or overexpression efficiency.

(G) RT-qPCR analysis confirming the knockdown efficiency of β-TrCP in RKO cells transfected with si-β-TrCP.

(H) Representative immunofluorescence images showing the subcellular colocalization of PD-L1 (red)

and β-TrCP (green) in RKO cells transiently overexpressing β-TrCP (OE-β-TrCP) or a control vector (OE-NC), following treatment with 40 μM EZA for 6 hours. Scale bar, 20 μm.

(I-J) RT-qPCR analysis of β-TrCP mRNA expression in H1975 cells. (I) Dose-dependent effect after 24-hour treatment with the indicated concentrations of EZA. (J) Time-dependent effect after treatment with 40 μM EZA.

(K) Western blot analysis (top) and quantification (bottom) of PD-L1 protein levels in RKO cells treated with cycloheximide (CHX, 50 μg/mL) over time, with or without a 12-hour pre-treatment with 40 μM EZA.

(L-M) Western blot analysis (top) and quantification (bottom) of β-TrCP protein levels in RKO cells. (L) Dose-dependent effect after 24-hour treatment with the indicated concentrations of EZA. (M) Time-dependent effect after treatment with 40 μM EZA. Data are presented as mean ± SD from three independent experiments. Statistical significance was determined by one-way ANOVA (A-F and H-J) or two-way ANOVA (G, K, L and M). *$P < 0.05$, **$P < 0.01$, ***$P < 0.001$, ****$P < 0.0001$; ns, not significant.

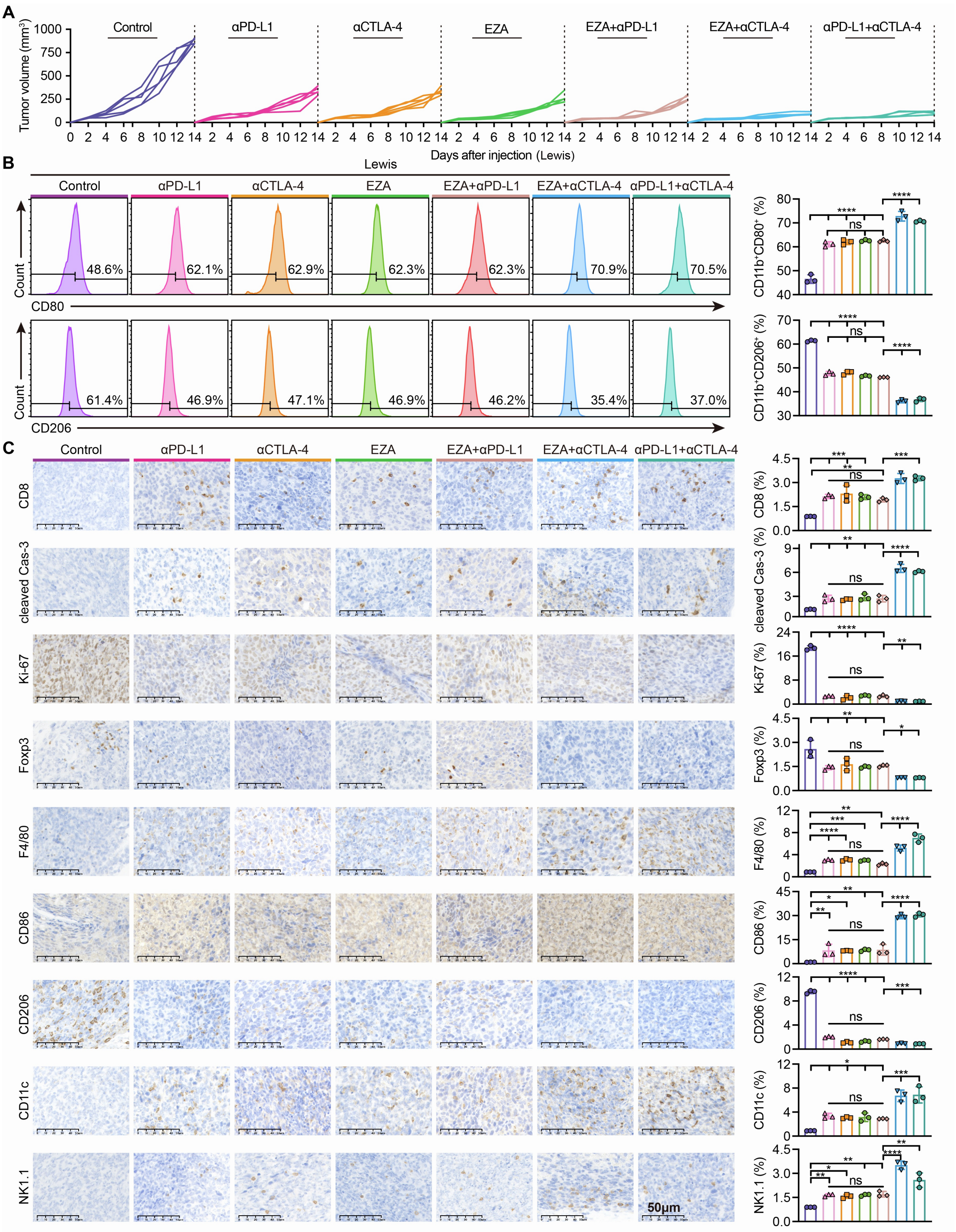

Figure S7. EZA synergizes with anti-CTLA-4 therapy to enhance antitumor immunity in the subcutaneous Lewis lung carcinoma model, related to Figure 6.

(A) Individual tumor growth curves for C57BL/6J mice bearing subcutaneous Lewis lung carcinoma (LLC) and treated with vehicle, EZA (50 mg/kg), anti-PD-L1 antibody (100 μg/mouse), anti-CTLA-4 antibody (100 μg per mouse), or their combination (n = 5 mice per group).

(B) Flow cytometric quantification of the following tumor-infiltrating macrophage subsets: M1 ($F4/80^{+}CD11b^{+}CD80^{+}$) and M2 ($F4/80^{+}CD11b^{+}CD206^{+}$).

(C) Immunohistochemical staining images and quantification of LLC tumor: CD8, Foxp3, cleaved Caspase-3, Ki-67, CD86, F4/80, CD206, CD11c, and NK1.1. Scale bar, 50 μm. Data are presented as

mean ± SD. Statistical significance was determined by one-way ANOVA (B, C). *$P < 0.05$, **$P < 0.01$, ***$P < 0.001$, ****$P < 0.0001$; ns, not significant.

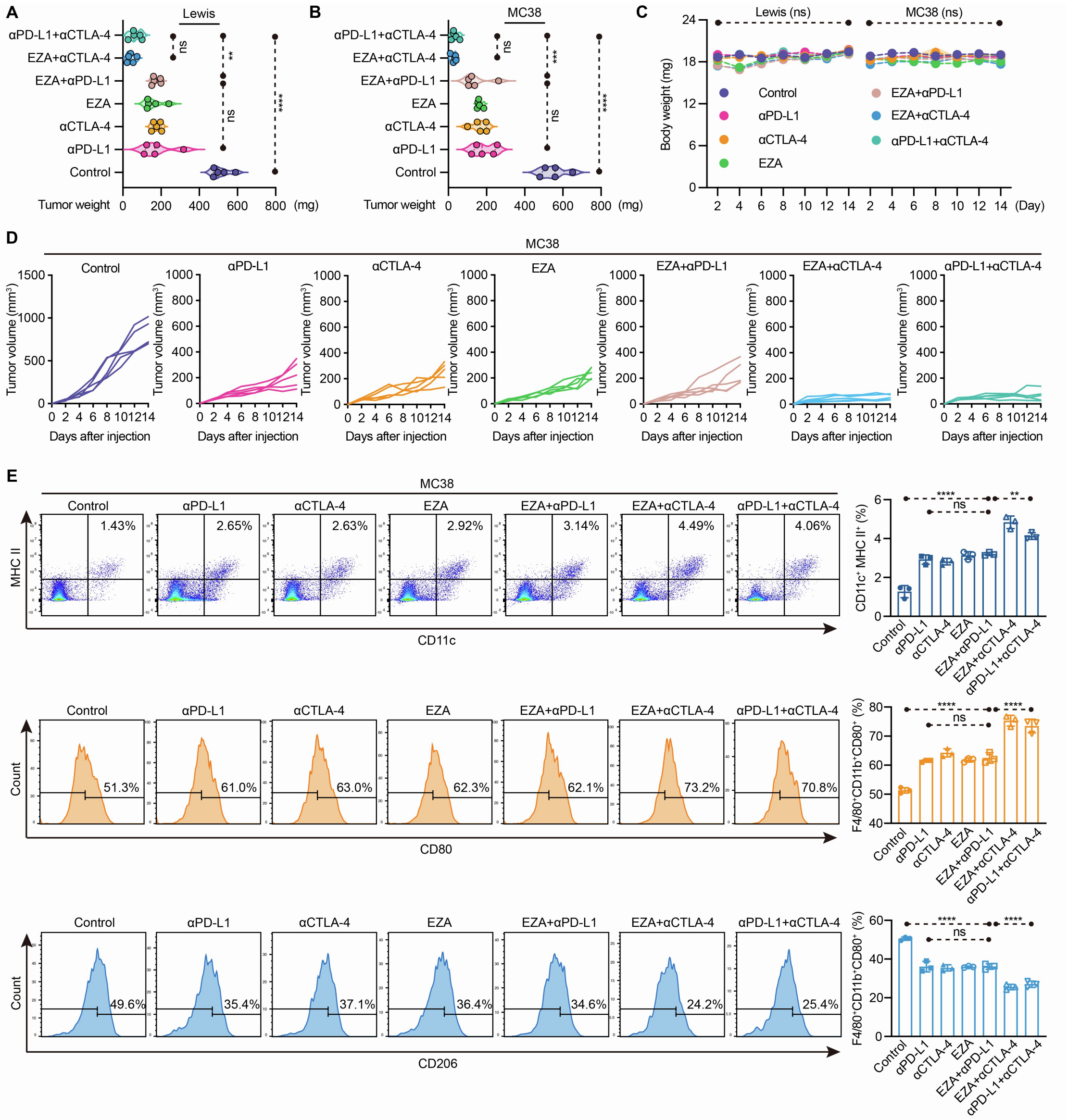

Figure S8. EZA synergizes with anti-CTLA-4 therapy to enhance antitumor immunity in the MC38 colorectal cancer subcutaneous model, related to Figure 6. (A) Schematic of the experimental design for drug treatment in MC38 tumor-bearing mice. Female C57BL/6J mice were treated with vehicle, EZA (50 mg/kg), anti-PD-L1 antibody (100 μg/mouse), anti-CTLA-4 antibody (100 μg/mouse), or the indicated combination (n = 5 mice per group).

(B) Body weight changes of mice during the treatment period.

(C) Final tumor weights at the experimental endpoint.

(D) Individual tumor growth curves for each mouse in the treatment groups described in (A). Tumor volume was measured twice weekly.

(E) Flow cytometric analysis of immune cell subsets within the tumor immune microenvironment. Quantified populations include mature dendritic cells ($CD11c^{+}MHC\text{-}II^{+}$), M1 macrophages ($F4/80^{+}CD11b^{+}CD86^{+}$), and M2 macrophages ($F4/80^{+}CD11b^{+}CD206^{+}$). Data in (B), (C), (D), and (E) are presented as mean ± SD. Statistical significance was determined by two-way ANOVA (B, D) or by one-way ANOVA (C, E). *$P < 0.05$, **$P < 0.01$, ***$P < 0.001$, ****$P < 0.0001$; ns, not significant.

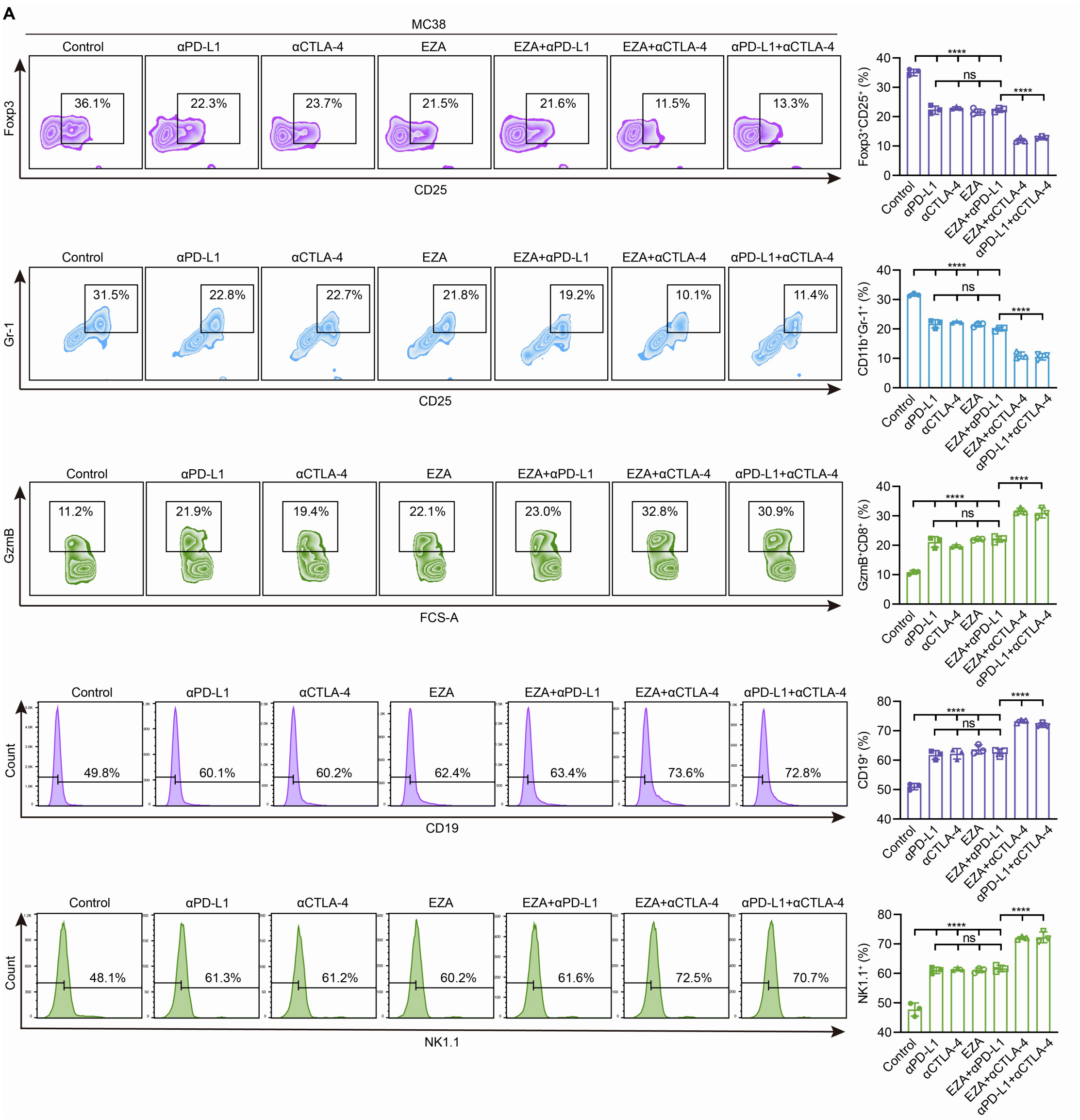

Figure S9. Flow cytometric analysis of immune cell modulation by the EZA/anti-CTLA-4 combination in the subcutaneous MC38 colorectal cancer model, related to Figure 6.

(A) Quantification of immune cell subsets within the tumor immune microenvironment of MC38 tumors from mice treated with vehicle, EZA (50 mg/kg), anti-PD-L1 antibody (100 μg/mouse), anti-CTLA-4 antibody (100 μg per mouse), or their combination. Quantified populations include Tregs ($CD4^+CD25^+Foxp3^+$), MDSCs ($CD11b^+Gr\text{-}1^+$), Granzyme B^+ cytotoxic lymphocytes, B cells ($CD19^+$), NK cells ($NK1.1^+$), and M1 macrophages ($F4/80^+CD11b^+CD86^+$). Data are presented as mean ± SD. Statistical significance was determined by one-way ANOVA (A). *$P < 0.05$, **$P < 0.01$, ***$P < 0.001$, ****$P < 0.0001$; ns, not significant.

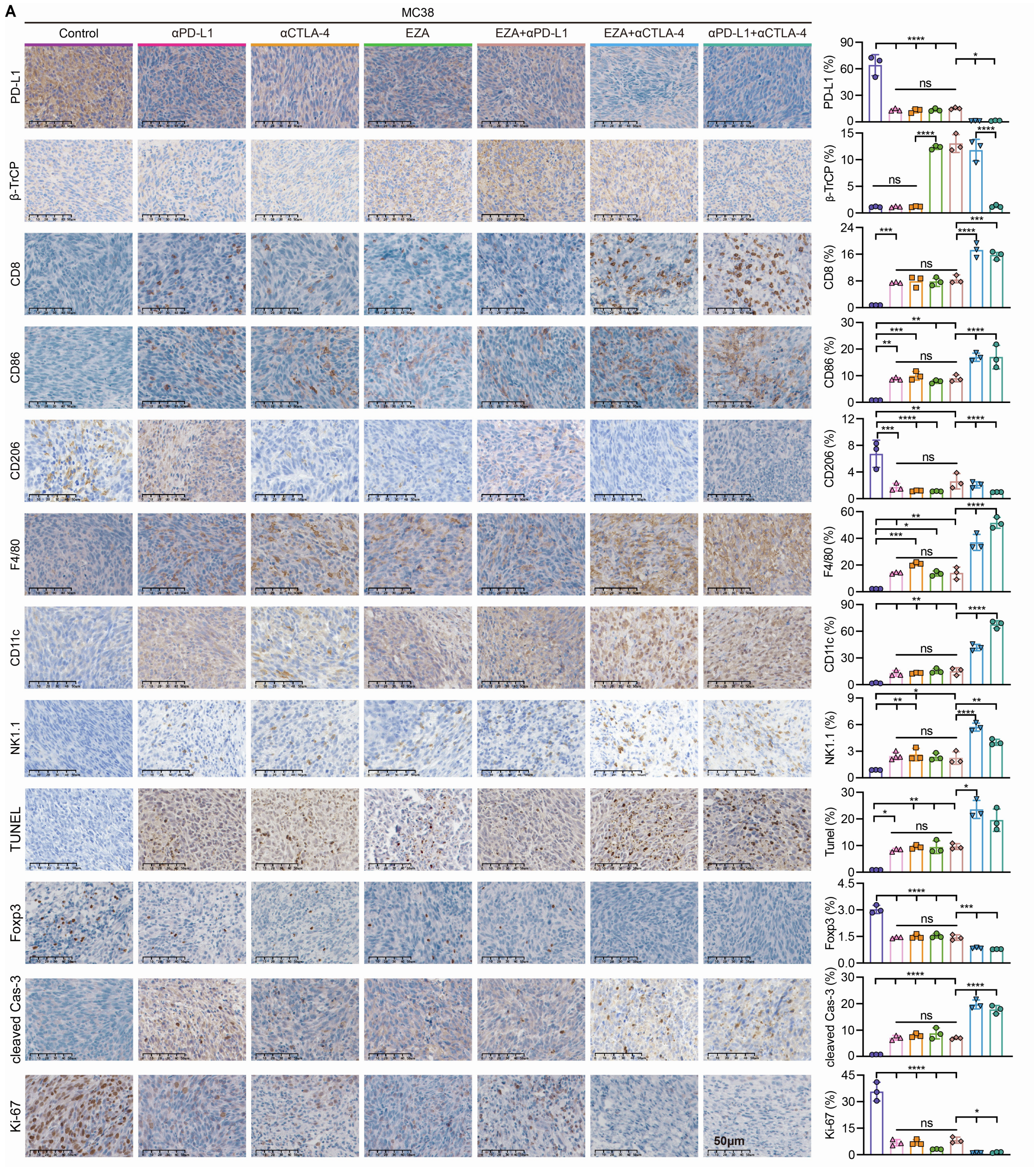

Figure S10. Immunohistochemical analysis of immune cell modulation by the EZA/anti-CTLA-4 combination in the subcutaneous MC38 colorectal cancer model, related to Figure 6.

(A) Immunohistochemical and quantitative analysis of the MC38 tumor microenvironment from mice treated with vehicle, EZA (50 mg/kg), anti-PD-L1 antibody (100 μg/mouse), anti-CTLA-4 antibody (100 μg/mouse), or their combination: PD-L1, β-TrCP, CD8, CD86, F4/80, CD206, CD11c, NK1.1, TUNEL, cleaved caspase-3, Ki-67, and Foxp3+ Tregs. scale bar: 50 μm. Data in (A) are presented as mean ± SD. Statistical significance was determined by one-way ANOVA (A). **P* < 0.05, ***P* < 0.01, ****P* < 0.001, *****P* < 0.0001; ns, not significant.

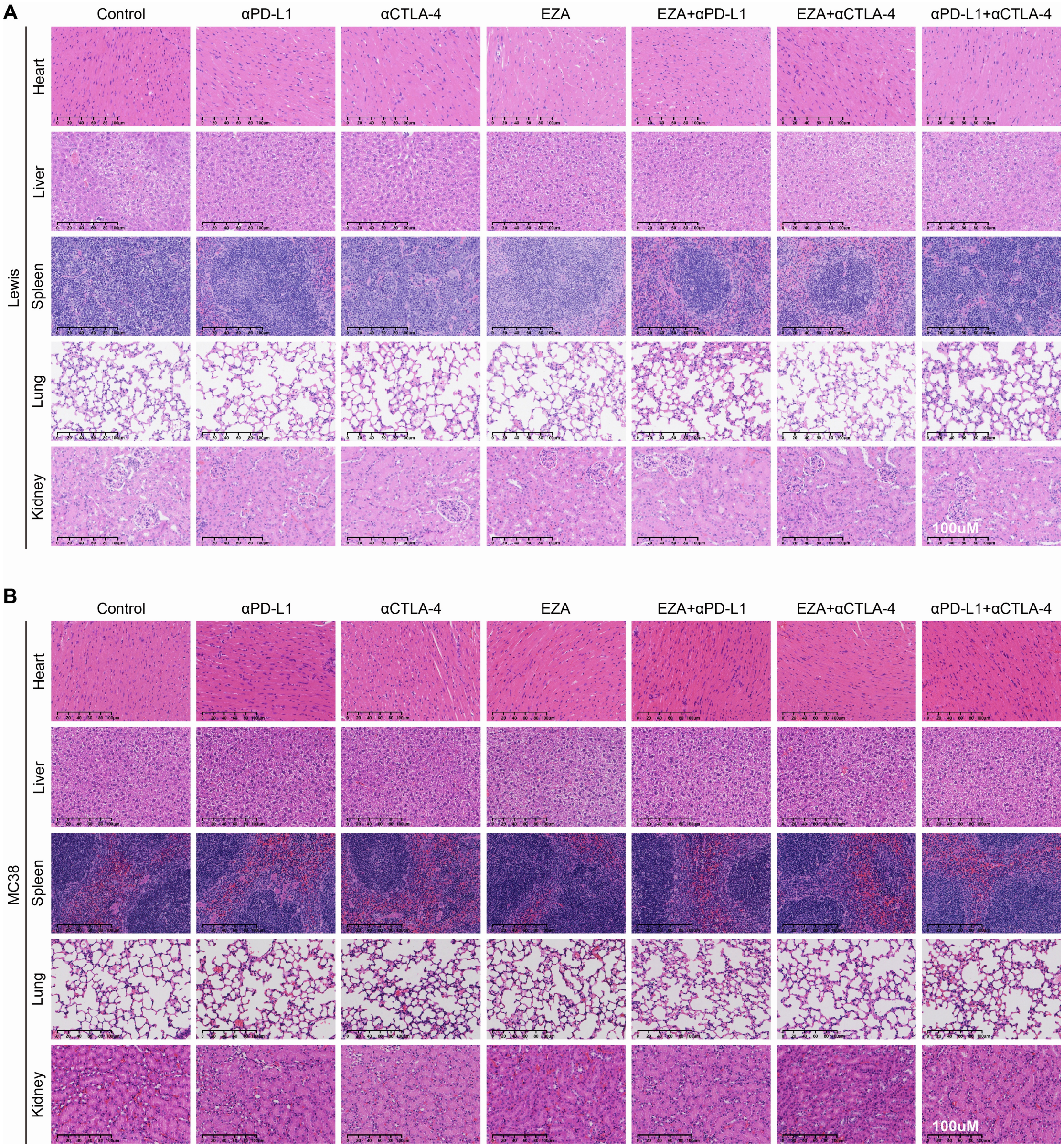

Figure S11. Safety evaluation of EZA and its combination therapy in subcutaneous tumor models, related to Figures 6.

(A-B) Histopathological assessment of major organs by H&E staining in: (A) subcutaneous Lewis lung carcinoma model; (B) subcutaneous MC38 colorectal cancer model (scale bar: 100 μm). No significant treatment-related pathological alterations were observed in any examined organ. Scale bar, 100 μm.

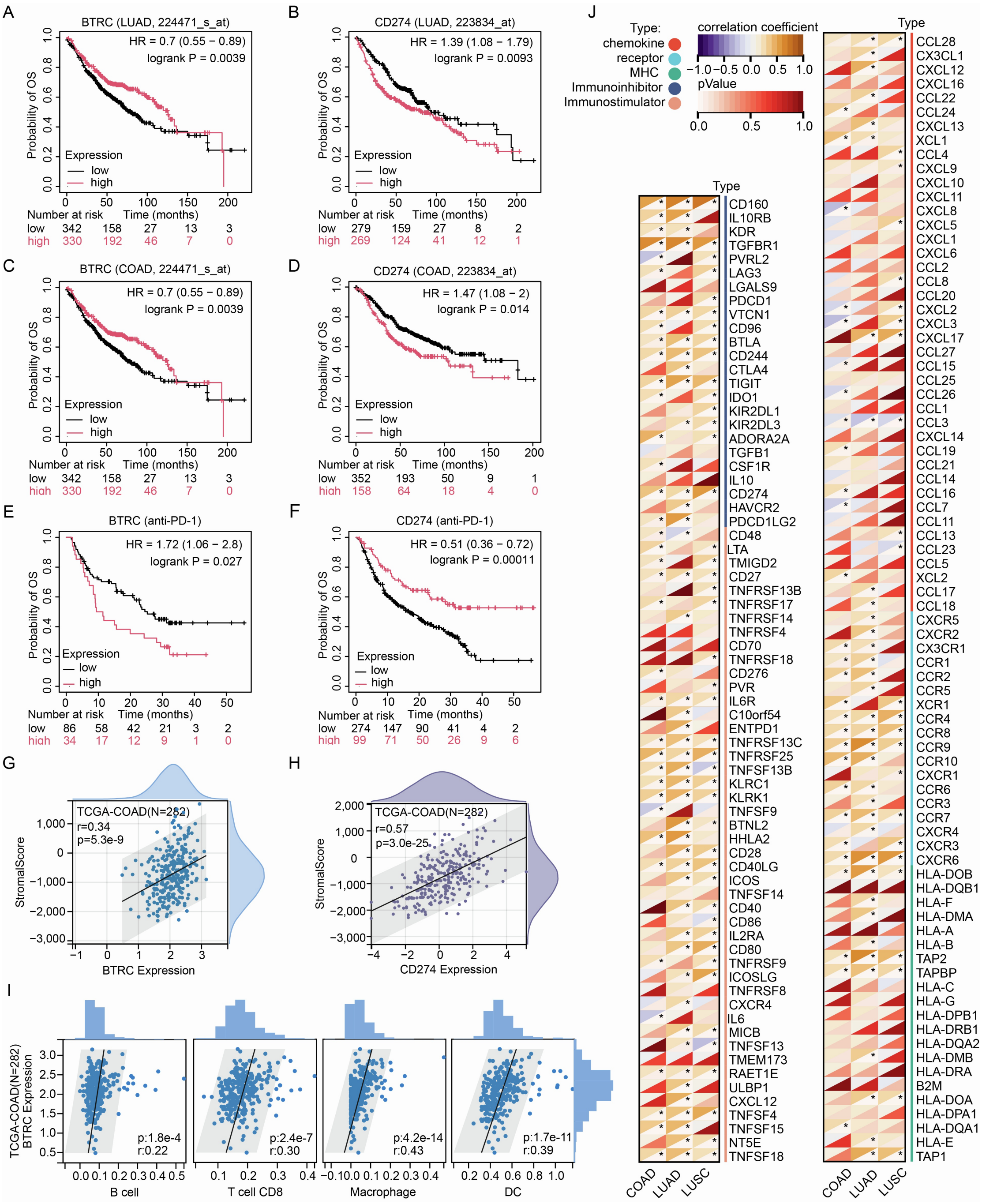

Figure S12. Prognostic and immunomodulatory significance of β-TrCP (BTRC) and PD-L1 (CD274) across cancer types, related to Figures 7.

(A-D) Kaplan-Meier overall survival (OS) analysis in the TCGA cohorts. Patients with lung adenocarcinoma (LUAD, A-B) and colon adenocarcinoma (COAD, C-D) were stratified by high or low expression of BTRC (A, C) or CD274 (B, D). *P* values were calculated using the log-rank test.

(E-F) Correlation between OS and BTRC (E) or CD274 (F) expression in a pancancer cohort of patients treated with anti-PD-1 therapy.

(G-H) Stromal scores estimated by the ESTIMATE algorithm in the TCGA-COAD cohort, plotted against expression levels of BTRC (G) and CD274 (H).

(I) Spearman correlation analysis between BTRC expression and the infiltration levels of selected immune cell types (B cells, CD8^{+} T cells, macrophages, dendritic cells) in the TCGA-COAD cohort.

(J) Heatmap of Pearson correlation coefficients between BTRC expression and 150 immune-related genes across TCGA-COAD, LUAD, and lung squamous cell carcinoma (LUSC) cohorts. Genes are grouped into five functional categories: chemokines (n=41), receptors (n=18), MHC molecules (n=21), immunoinhibitors (n=24), and immunostimulators (n=46). Survival differences in (A-F) were assessed using the log-rank test. Correlations in (G-I) were evaluated by Spearman's rank correlation, while the correlation matrix in (J) was generated using Pearson correlation. *$P < 0.05$.

Supplementary tables

Table S1. Baseline Demographic, Clinical, and Pathologic Characteristics of NSCLC Patients Treated with Neoadjuvant Immunochemotherapy, related to Figure 7

**Variable**	**Overall** N = 32[1]	**Non-Responder** N = 16[1]	**Responder** N = 16[1]	***P*-value**[2]
**Response**				<0.001
pCR[3]	10 (31%)	0 (0%)	10 (63%)	
MPR[4]	6 (19%)	0 (0%)	6 (38%)	
Non-MPR	16 (50%)	16 (100%)	0 (0%)	
**Sex**				>0.9
Female	5 (16%)	3 (19%)	2 (13%)	
Male	27 (84%)	13 (81%)	14 (88%)	
**Age**				0.3
<65	19 (59%)	8 (50%)	11 (69%)	
≥65	13 (41%)	8 (50%)	5 (31%)	
**Histology**				0.015
LUAD[5]	9 (28%)	8 (50%)	1 (6.3%)	
LUSC[6]	23 (72%)	8 (50%)	15 (94%)	
**T**				>0.9
1b	1 (3.1%)	0 (0%)	1 (6.3%)	
1c	1 (3.1%)	1 (6.3%)	0 (0%)	
2a	4 (13%)	2 (13%)	2 (13%)	
2b	7 (22%)	4 (25%)	3 (19%)	
3	10 (31%)	5 (31%)	5 (31%)	
4	9 (28%)	4 (25%)	5 (31%)	
**N**				0.3
0	6 (19%)	4 (25%)	2 (13%)	
1	5 (16%)	1 (6.3%)	4 (25%)	
2	20 (63%)	11 (69%)	9 (56%)	
3	1 (3.1%)	0 (0%)	1 (6.3%)	
**cTNM stage**				0.7
IB	2 (6.3%)	1 (6.3%)	1 (6.3%)	

Variable	Overall<br>N = 32[1]	Non-Responder<br>N = 16[1]	Responder<br>N = 16[1]	*P*-value[2]
IIA	1 (3.1%)	1 (6.3%)	0 (0%)	
IIB	6 (19%)	4 (25%)	2 (13%)	
IIIA	9 (28%)	3 (19%)	6 (38%)	
IIIB	13 (41%)	7 (44%)	6 (38%)	
IIIC	1 (3.1%)	0 (0%)	1 (6.3%)	
**TRG**[7]				<0.001
0	10 (31%)	0 (0%)	10 (63%)	
1	6 (19%)	0 (0%)	6 (38%)	
2	14 (44%)	14 (88%)	0 (0%)	
3	2 (6.3%)	2 (13%)	0 (0%)	
**PS**[8]				0.7
≥ 1	11 (34%)	6 (38%)	5 (31%)	
< 1	21 (66%)	10 (62%)	11 (69%)	
**Adverse effect**				0.4
0	22 (69%)	9 (56%)	13 (81%)	
1	6 (19%)	4 (25%)	2 (13%)	
2	4 (13%)	3 (19%)	1 (6.3%)	
**Change in CT value (ΔCT)**[9]	0.35 (0.17, 0.54)	0.22 (0.12, 0.36)	0.44 (0.27, 0.82)	0.024
**PFS (month)**[10]	27.6 ± 16.8	18.9 ± 14.2	36.4 ± 13.8	0.036
**OS (month)**[11]	29.4 ± 14.8	22.5 ± 13.1	36.2 ± 13.5	0.022

[1]n (%); Median (Q1, Q3); [2]Fisher's exact test; Pearson's Chi-squared test; Wilcoxon rank sum test; [3]pCR, pathological complete response; [4]MPR, major pathologic response;[5]LUAD, Lung Adenocarcinoma;[6]LUSC, Lung Squamous Cell Carcinoma; [7]TRG, Tumor Regression Grade; [8]PS, performance status; [9]ΔCT, Ratio of Post-treatment to Pre-treatment Maximum Tumor Diameter on Computed Tomography; [10]PFS, Progression-Free Survival; [11]OS, Overall Survival.

Table S2. Sequences of siRNAs and PCR Primers, related to STAR Methods

siRNA/PCR Primer	Sequences (5′→3′)
si-β-TrCP-1	GCGUUGUAUUCGAUUUGAUAA
si-β-TrCP-2	GCUGAACUUGUGUGCAAGGAA
si-PD-L1-1	TCAATTGTCATATTGCTAC
si-PD-L1-2	TTGACTCCATCTTTCTTCA
si-HRD1-1	UGUCUGGCCUUCACCGUUU
si-HRD1-2	CCAAGAGACUGCCCUGCAA
si-ARIH1-1	CGAGAUAUUUCCCAAGAUUUU
si-ARIH1-2	CCAUGUUGUUAAAGUCCAAUA
si-SPOP-1	CACAAGGCUAUCUUAGCAGCU
si-SPOP-2	CUCCUACAUGUGGACCAUCAA
si-MARCH8-1	GGACATTTCATGAGTCATT
si-MARCH8-2	GGAAGAGACTCAAGGCCTA
si-STUB1-1	GCAGUCUGUGAAGGCGCACUU
si-STUB1-2	CCCAAGUUCUGCUGUUGGACU
PD-L1 *F*	GGCATTTGCTGAACGCAT
PD-L1 *R*	CAATTAGTGCAGCCAGGT
T180A PD-L1 *F*	GGTAAGACCgCCACCACCAATTTCAAG
T180A PD-L1 *R*	ATTGGTGGTGGcGGTCTTACCACTCAGG
S184A PD-L1 *F*	ACCACCAATgCCAAGAGAGAGGAGAAGCTTTTC
S184A PD-L1 *R*	TCCTCTCTCTTGGcATTGGTGGTGGTGGTCTTA
β-TrCP *F*	TGGCTCATCTGACAACACTATC
β-TrCP *R*	CGAATACAACGCACCAATTCC
β-actin *F*	ATTCCTATGTGGGCGACGAG
β-actin *R*	CCAGATTTTCTCCATGTCGTCC
HRD1 *F*	TGCGTAACATCCACACACTG
HRD1 *R*	CTTTGAGTTTGTATCTTGGATGCC
ARIH1 *F*	GCATCTTCAGGTAGCACAAGGC

ARIH1 *R*	ACTTTGATGGAAACCTGGAGAA
SPOP *F*	GCCAGTGAAATACGAGTTAGGG
SPOP *R*	CCTGGAGCGCTTAAAGGTCA
STUB1 *F*	CGAATACAACGCACCAATTCC
STUB1 *R*	TCAAGGAGCAGGGCAATCGTCT
MARCH8 *F*	AGTGACATTCCACGTCATTGC
MARCH8 *R*	GATCTCCTCAGCAGTACGGTC
