# Supplementary material for: Repurposed ethoxzolamide reprograms antitumor immunity through β-TrCP-dependent PD-L1 ubiquitination
Source: Cell Rep Med. 2026 Jul 13;7(7):102920. doi: 10.1016/j.xcrm.2026.102920 (PMC13400190; doi:10.1016/j.xcrm.2026.102920)
Supplement: Document S2. Article plus supplemental information [file mmc2.pdf]

Cell Reports
Medicine

Article

Repurposed ethoxzolamide reprograms antitumor immunity through β-TrCP-dependent PD-L1 ubiquitination

Graphical abstract

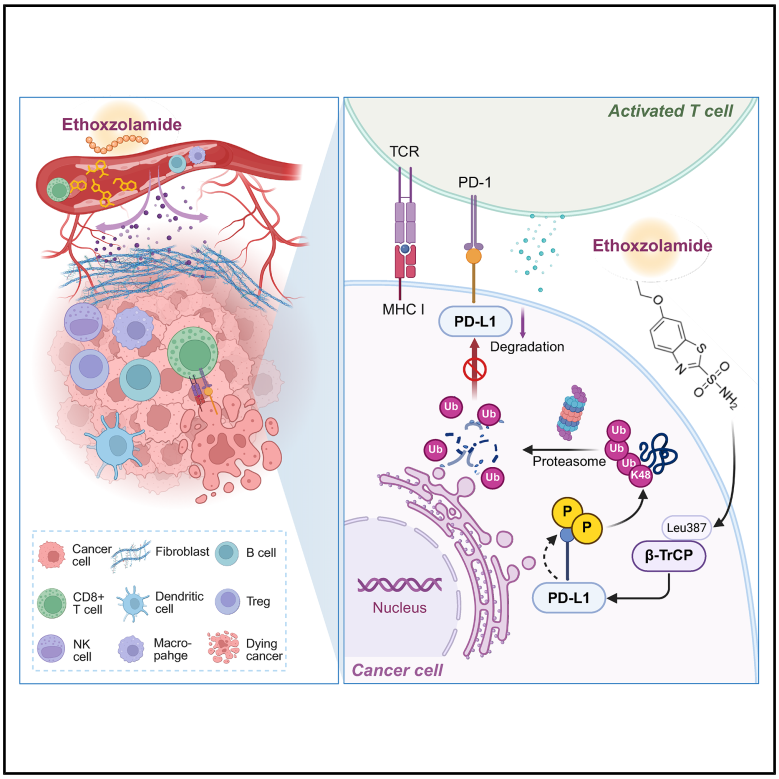

Highlights

- Ethoxzolamide is repurposed as a negative regulator of PD-L1 in cancer immunotherapy
- Ethoxzolamide triggers PD-L1 degradation by binding to β-TrCP Leu387
- Ethoxzolamide remodels tumor immune microenvironment and synergizes with anti-CTLA-4
- The PD-L1/β-TrCP axis may associate with immunotherapy response

Authors

Xuwen Lin, Qun Wang, Mengting Xu, ..., Weidong Zhang, Sanhong Liu, Xinying Xue

Correspondence

wdzhangy@hotmail.com (W.Z.),
liush@shutcm.edu.cn (S.L.),
xuexinying@xwhosp.org (X.X.)

In brief

Lin et al. reveal that the drug ethoxzolamide triggers the degradation of PD-L1, an immune-suppressing protein on cancer cells. This reprogramming of the tumor environment overcomes resistance to immunotherapy, suggesting a potential strategy for treating solid tumors.

Lin et al., 2026, Cell Reports Medicine 7, 102920
July 21, 2026 © 2026 The Author(s). Published by Elsevier Inc.
https://doi.org/10.1016/j.xcrm.2026.102920

CellPress

Cell Reports Medicine

CellPress
OPEN ACCESS

Article

Repurposed ethoxzolamide reprograms antitumor immunity through β-TrCP-dependent PD-L1 ubiquitination

Xuwen Lin,[1,7] Qun Wang,[2,7] Mengting Xu,[2,7] Dianping Yu,[2] Hongmei Hu,[2] Qing Zhang,[2] Jiannan Yao,[3] Mei Xie,[1] Hanchi Xu,[2] Xuefeng Zang,[1] Jia Li,[1] Yu Chen,[1] Linyang Li,[2] Xiaoyu Tao,[2] Xinru Li,[2] Simeng Li,[2] Shize Xie,[4] Yating Tian,[4] Weidong Zhang,[4,5,*] Sanhong Liu,[2,*] and Xinying Xue[1,6,8,*]
[1]Department of Respiratory and Critical Care, Xuanwu Hospital of Capital Medical University, National Clinical Research Center for Geriatric Diseases, Beijing 100053, China
[2]State Key Laboratory of Discovery and Utilization of Functional Components in Traditional Chinese Medicine, Shanghai Frontiers Science Center of TCM Chemical Biology, Institute of Interdisciplinary Integrative Medicine Research, Shanghai 201203, China
[3]Beijing Chao-Yang Hospital Department of Oncology, Capital Medical University, Beijing 100006, China
[4]Department of Phytochemistry, School of Pharmacy, Second Military Medical University, Shanghai 200433, China
[5]Institute of Medicinal Plant Development, Chinese Academy of Medical Sciences & Peking Union Medical College, Beijing 100193, China
[6]Department of Respiratory and Critical Care, Shandong Second Medical University, Weifang 261053, China
[7]These authors contributed equally
[8]Lead contact
*Correspondence: wdzhangy@hotmail.com (W.Z.), liush@shutcm.edu.cn (S.L.), xuexinying@xwhosp.org (X.X.)
https://doi.org/10.1016/j.xcrm.2026.102920

SUMMARY

Despite the clinical efficacy of PD-1/PD-L1 blockade in solid tumors, resistance mediated by PD-L1 protein stabilization necessitates alternative strategies. Our study identifies ethoxzolamide (EZA), a carbonic anhydrase inhibitor, as a negative regulator of PD-L1. EZA binds to the Leu387 residue of the E3 ubiquitin ligase β-TrCP, triggering K48-linked polyubiquitination and proteasomal degradation of PD-L1. Functionally, EZA downregulates tumor cell PD-L1, restoring T cell-mediated cytotoxicity *in vitro*. In Lewis lung carcinoma and MC38 murine models, EZA reprograms the tumor immune microenvironment (reducing MDSC/Treg infiltration while bolstering cytotoxic response). EZA synergizes with anti-CTLA-4 therapy to overcome treatment resistance. Clinical analysis of a neoadjuvant immunotherapy cohort of non-small cell lung cancer (NSCLC) patients reveals that high PD-L1 with low β-TrCP expression associates with superior response, suggesting that this axis may warrant further investigation. Our findings elucidate an antitumor mechanism of EZA and expand its therapeutic potential.

INTRODUCTION

Programmed death-ligand 1 (PD-L1) is a pivotal immune checkpoint molecule that enables tumor cells to evade immune surveillance by binding to PD-1 on T cells and suppressing their activation.[1,2] Immune checkpoint therapy (ICT) targeting the PD-1/PD-L1 axis has revolutionized treatment for diverse malignancies, including melanoma, non-small cell lung cancer (NSCLC), and colorectal cancer (CRC).[2–5] However, two major clinical challenges remain. Foremost, resistance poses a substantial hurdle: even among patients with these ICT-sensitive tumor types, a substantial proportion exhibit primary or acquired resistance, leading to treatment failure.[6,7] Second, immune-related adverse events (irAEs) present a critical concern: a subset of patients experience irAEs affecting vital organs, which not only severely impact quality of life but also disrupt treatment continuity and efficacy.[8,9] These challenges underscore the limitations inherent in current antibody-based PD-1/PD-L1 blockade strategies and highlight the urgent clinical need for alternative therapeutic strategies that modulate endogenous PD-L1 expression.[10,11]

In tumor cells, PD-L1 protein expression is complexly regulated at both the transcriptional level and through dynamic post-translational mechanisms such as the ubiquitin-proteasome system (UPS) and lysosomal degradation pathways.[12–16] These coordinated processes determine the PD-L1 protein abundance in the tumor immune microenvironment (TIME). Thus, for tumors with high PD-L1 expression, developing targeted strategies to promote PD-L1 degradation is highly clinically important.[17] Beyond monoclonal antibodies, research efforts have also focused on small-molecule inhibitors, including BMS compounds and peptide mimetics, which are designed to directly disrupt the PD-1/PD-L1 interaction.[18,19] At present, the development of small-molecule-mediated PD-L1 selective degraders, such as proteolysis-targeting chimeras (PROTACs) and molecular glues, represents a promising frontier.[16,17,20] This strategy offers distinct potential advantages: (1) it may overcome resistance by achieving more complete elimination

© 2026 The Author(s). Published by Elsevier Inc.
This is an open access article under the CC BY license (http://creativecommons.org/licenses/by/4.0/).

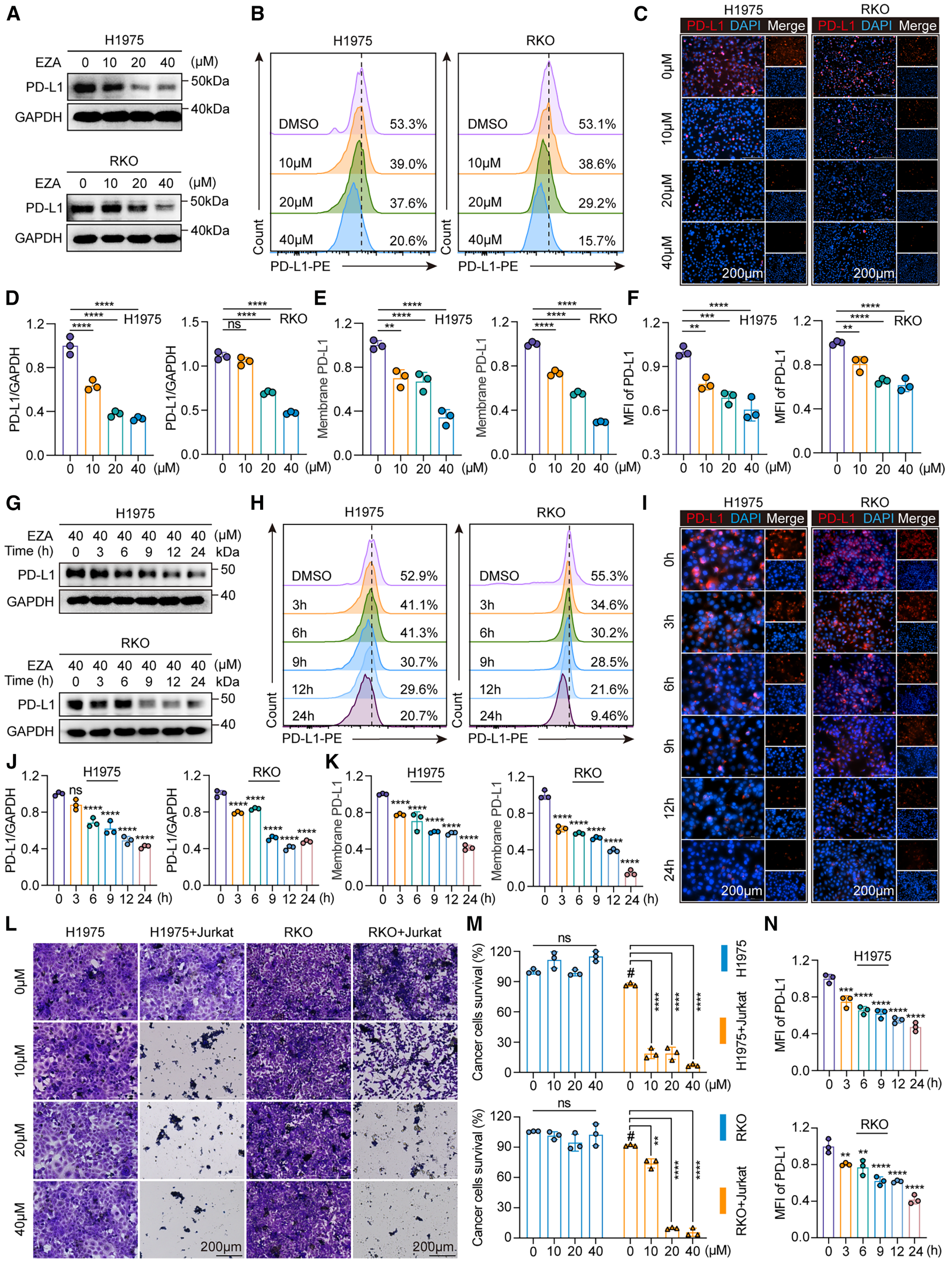

Figure 1. EZA enhances T cell-mediated antitumor immunity by downregulating PD-L1 expression

(A) Western blot analysis of PD-L1 protein levels in H1975 and RKO cells treated with EZA (0, 10, 20, 40 μM) for 24 h.

(B) Flow cytometry analysis of membrane PD-L1 expression in H1975 and RKO cells treated with EZA (0, 10, 20, 40 μM) for 24 h.

(C) Immunofluorescence (IF) staining for membrane PD-L1 in H1975 and RKO cells treated with EZA (0, 10, 20, 40 μM) for 24 h. Scale bars, 200 μm.

(D–F) (D) Quantification of PD-L1 protein levels from (A), (E) mean fluorescence intensity (MFI) of PD-L1 from flow cytometry in (B), and (F) relative fluorescence intensity from IF images in (C).

(legend continued on next page)

of PD-L1 rather than mere receptor blockade[20] and (2) it has the potential to mitigate irAEs through spatiotemporally controlled or tissue-selective degradation.[20] Furthermore, when compared with monoclonal antibodies, small-molecule immunomodulators provide superior tissue penetration, controllable pharmacokinetic profiles, and lower immunogenicity,[21] establishing them as promising candidates for next-generation cancer immunotherapies.[20]

Carbonic anhydrase inhibitors (CAIs) have traditionally been employed for diuresis and for the treatment of glaucoma and epilepsy.[22] Recent research, however, has revealed their potential antitumor activity.[22] Ethoxzolamide (EZA), an amphiphilic second-generation sulfonamide-based CAI, has been repurposed as an anti-infective agent due to its efficacy against *Neisseria gonorrhoeae* and *Helicobacter pylori*.[22–24] Notably, previous studies have indicated that CAIs may exert adjunctive anticancer effects by targeting CA isoforms (such as CA IX and CA XII) that are overexpressed within the tumor metabolic microenvironment.[22,25] These effects primarily involve the modulation of tumor metabolism and pH homeostasis; for instance, CAIs like EZA can induce microenvironmental acidosis.[22,25] However, existing research has focused predominantly on nonimmune mechanisms. Building upon this foundation, our study revealed that EZA functions as a PD-L1-targeting degrader through an antitumor immune mechanism independent of CA inhibition. By integrating multiomics analyses, protein-protein interaction assays, and functional validation both *in vitro* and *in vivo*, we demonstrate that EZA binds to the E3 ubiquitin ligase β-transducin repeat-containing protein (β-TrCP/BTRC). This interaction triggers the phosphorylation of PD-L1, initiates a K48-linked polyubiquitination cascade, and ultimately promotes proteasome-dependent PD-L1 degradation. This finding reveals a mechanism of EZA and suggests its potential application in cancer immunotherapy, independent of its classical CA inhibitory activity. Moreover, this work provides a critical mechanistic blueprint for the development of small-molecule degraders targeting the PD-L1-mediated immune evasion pathway.

RESULTS

EZA enhances T cell-mediated antitumor immunity by downregulating PD-L1 expression

Our study initially utilized RKO colon cancer cells as a model system to screen a small-molecule compound library, with the goal of identifying potential compounds capable of downregulating PD-L1 expression and thereby enhancing the efficacy of PD-L1-targeted immunotherapy (screening workflow shown in Figure S1A). After primary screening, we further investigated the effect of EZA on PD-L1 protein turnover using lung cancer (H1975) and colon cancer (RKO) cell models, both of which exhibit high endogenous PD-L1 expression (Figure 1A). The results demonstrated that EZA treatment markedly reduced PD-L1 protein levels in both cell lines, in a dose- and time-dependent manner (Figures 1A, 1D, 1G, and 1J). In addition, compared with the CRBN-recruiting PROTAC PD-L1 degrader PA8,[26] EZA demonstrated significant PD-L1 degradation activity (Figure S1B). Flow cytometry and immunofluorescence (IF) analyses further confirmed that EZA effectively suppressed membrane PD-L1 expression, with inhibition correlating with higher concentrations and longer treatment durations (Figures 1B, 1C, 1E, 1F, 1H, 1I, 1K, and 1N). Given that EZA is a known CAI, a critical subsequent question was whether its canonical enzymatic inhibition contributed to PD-L1 reduction. To address this, we treated H1975 and RKO cells with two established CAIs: methazolamide (CA II preferring) and acetazolamide (active against CA IX) (Figures S1C and S1D). Under these conditions, neither compound significantly altered PD-L1 protein levels, indicating that PD-L1 downregulation is a function of EZA that is not shared by the other CAIs tested. Cell viability and EdU (5-ethynyl-2′-deoxyuridine)proliferation assays further confirmed that there was no significant cytotoxicity at the tested EZA concentrations (Figures S1E–S1G), demonstrating that PD-L1 downregulation was independent of growth inhibition.

Previous studies have shown that PD-L1 on tumor cells mediates T cell tolerance via PD-1 binding and that inhibiting PD-L1 increases tumor cell sensitivity to T cell-mediated killing.[27] As EZA markedly reduced PD-L1 expression *in vitro*, we further explored its impact on tumor immune function. Tumor cells were cocultured with activated engineered Jurkat cells overexpressing PD-1 and granzyme B in a T cell-killing assay. The results showed that EZA treatment enhanced T cell-mediated tumor cell killing in H1975 and RKO cells (Figures 1L and 1M). Similarly, pretreatment with EZA significantly enhanced the killing capacity of primary T cells against tumor cells (Figure S1H).

EZA suppresses subcutaneous tumor growth via immune activation

To validate the enhanced T cell activity observed *in vitro*, we evaluated the antitumor efficacy of EZA *in vivo* using subcutaneous tumor models of Lewis lung carcinoma (LLC) (Figures 2A–2E) and MC38 colon cancer in C57BL/6J mice (Figures S2A–S2E). The mice received oral gavage of EZA (25 or 50 mg/kg) from day 4 to day 15 post-LLC inoculation. The results revealed that EZA inhibited LLC growth in a dose-dependent manner, with

(G) Western blot analysis of PD-L1 protein levels in H1975 and RKO cells treated with 40 μM EZA for the indicated durations (0, 3, 6, 9, 12, 24 h).
(H) Flow cytometry analysis of membrane PD-L1 expression in H1975 and RKO cells treated with 40 μM EZA for the indicated durations.
(I) Representative IF images of membrane PD-L1 in H1975 and RKO cells treated with 40 μM EZA for the indicated durations. Scale bars, 200 μm.
(J and K) (J) Quantification of PD-L1 protein levels from (G). (K) PD-L1 MFI from (H).
(L) Crystal violet staining of residual adherent H1975 or RKO cells following a 48-h coculture with PD-1-overexpressing Jurkat T cells. Tumor cells were pretreated with the indicated concentrations of EZA prior to coculture. Scale bars, 200 μm.
(M) Quantification of residual tumor cells from (L), normalized to the untreated control group without T cells. #$p < 0.05$ vs. the corresponding tumor cell-only control (H1975 control or RKO control); **$p < 0.01$ and ****$p < 0.0001$ vs. the corresponding untreated coculture group (H1975 + T cells or RKO + T cells).
(N) Quantification of relative fluorescence intensity from IF images in (I).
Data in (D)–(F), (J)–(K), (M), and (N) are presented as mean ± SD from three independent experiments. Statistical significance was determined by one-way ANOVA (D–F, J–K, and N) or two-way ANOVA (M). *$p < 0.05$, **$p < 0.01$, ***$p < 0.001$, ****$p < 0.0001$; ns, not significant. See also Figure S1.

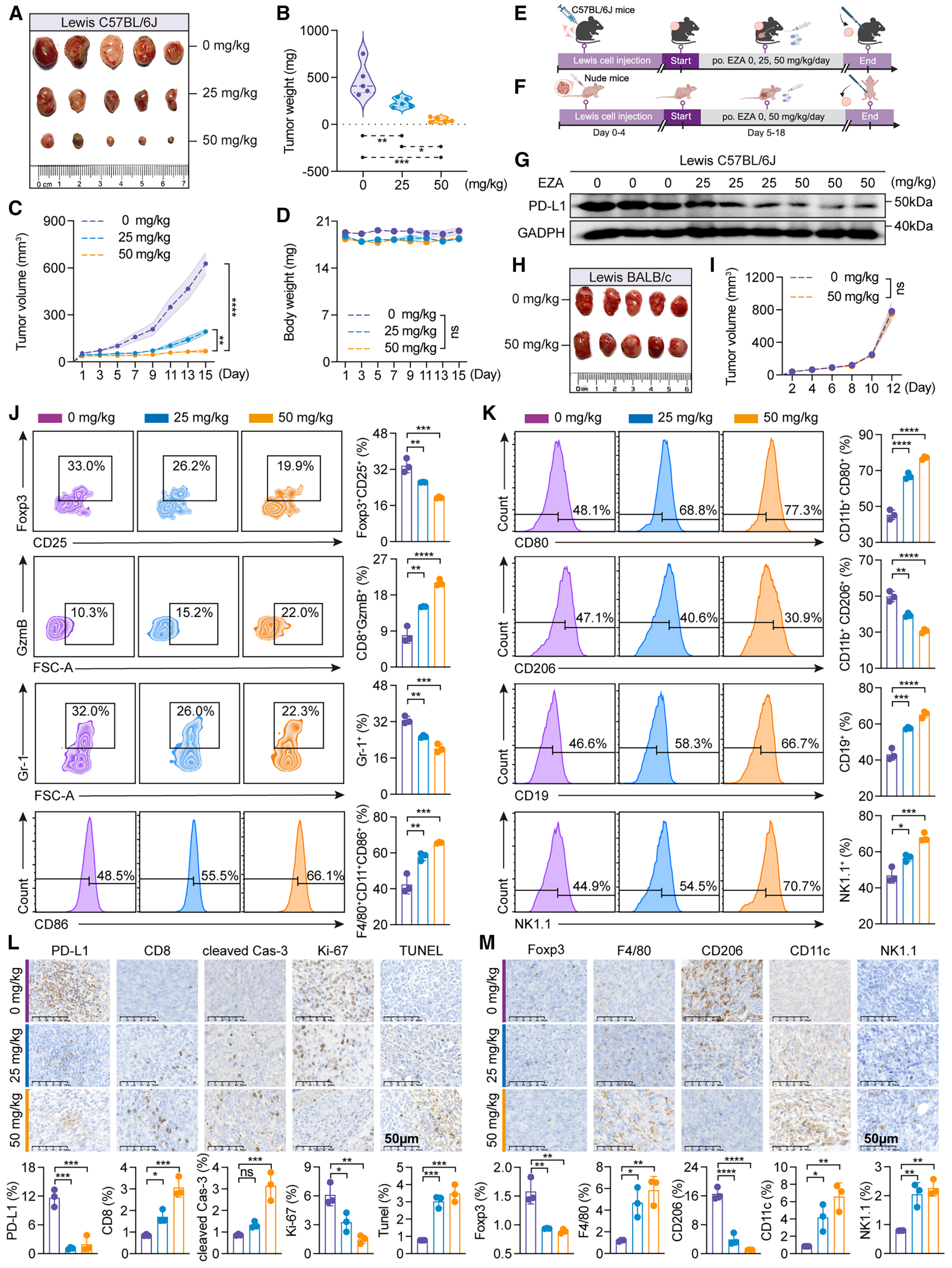

Figure 2. EZA suppresses subcutaneous tumor growth via immune activation

(A) Representative images of excised tumors from Lewis lung carcinoma (LLC)-bearing C57BL/6J mice treated with vehicle or EZA (25 or 50 mg/kg) (*n* = 5 mice per group).

(B–D) Tumor weights (B), tumor growth curves (C), and body weight changes (D) of mice in (A).

(E and F) Schematic of the experimental designs in C57BL/6J mice (E) and nude mice (F).

(G) Western blot analysis of PD-L1 expression in LLC tumors from C57BL/6J mice treated with vehicle or EZA (25 or 50 mg/kg).

(legend continued on next page)

tumor inhibition rates of 69.26% and 89.13% at 25 and 50 mg/kg, respectively (Figures 2A and 2C). Similar antitumor effects were observed in the MC38 model, with tumor inhibition rates of 58.5% and 83.24% at 25 and 50 mg/kg, respectively (Figures S2A–S2C). Strikingly, in T cell-deficient nude mice, EZA failed to suppress both LLC (Figures 2F, 2H, 2I, and S4A–S4D) and MC38 (Figures S4F–S4K) tumors, demonstrating that its efficacy depends on functional T cell-mediated immunity. Importantly, neither C57BL/6J nor nude mice experienced significant body weight loss (Figures 2B, S2D, S4A, and S4H) and histopathological analysis (H&E staining) of major organs revealed no signs of toxicity (Figures S3C, S3D, S4E, and S4L), confirming that EZA is both effective and safe *in vivo*.

We next examined PD-L1 levels and immune cell infiltration within tumor tissue. As presented in Figure 2G, EZA significantly reduced PD-L1 expression in a dose-dependent manner. Flow cytometry analysis of the TIME revealed that (1) EZA markedly reduced the frequency of immunosuppressive Tregs and myeloid-derived suppressor cells (MDSCs)[28,29]; (2) it increased the abundance of granzyme B$^+$ effector T cells, CD19$^+$ B cells, NK1.1$^+$ natural killer (NK) cells, and activated M1 macrophages (F4/80$^+$CD11b$^+$CD86$^+$/CD80$^+$); and (3) it was accompanied by a significant reduction in M2 macrophages (F4/80$^+$CD11b$^+$CD206$^+$) (Figures 2J and 2K). These immune reprogramming effects were further confirmed via immunohistochemistry (IHC) (Figure 2L and 2M). Additionally, EZA induced tumor cell apoptosis, as evidenced by increasing cleaved caspase-3 activity and TUNEL$^+$ signals and significantly reduced proliferative activity marked by Ki-67 staining. In the MC38 model, both flow cytometry and IHC consistently demonstrated TIME remodeling phenotypes (Figures S2F, S2G, S3A, and S3B). Together, these data indicated that EZA mediates the selective degradation of PD-L1 on tumor cells, reverses PD-1/PD-L1-mediated T cell suppression, and remodels the immunosuppressive TIME to elicit robust antitumor immunity.

EZA can suppress AOM/DSS-induced colorectal cancer progression

Colitis-associated CRC (CAC) arises from chronic inflammation and is driven by a complex TIME.[30] The azoxymethane (AOM)/dextran sulfate sodium (DSS)-induced murine model mimics human CAC pathogenesis and is widely used for studying inflammation-driven carcinogenesis and therapeutic evaluation.[4,31,32] Using this model, we investigated the impact of EZA on colitis-associated tumorigenesis and its underlying immunomodulatory mechanisms[33] (Figure 3A). As shown in Figure 3B, EZA treatment significantly suppressed tumor progression in AOM/DSS-treated C57BL/6J mice, as evidenced by a reduced colonic tumor burden, fewer adenoma-like lesions, and attenuated high-grade dysplasia and adenocarcinoma formation (Figure 3C). Additionally, EZA treatment alleviated body weight loss, prevented colon shortening, and decreased tumor number and diameter (Figures 3D–3F). Quantitatively, EZA achieved a 96.10% reduction in large tumors (>4 mm), a 14.29% decrease in medium-sized tumors (2–4 mm), and a 57.58% overall reduction in total tumor count, underscoring its efficacy in inflammation-driven CRC. Histopathological assessment confirmed the absence of significant organ toxicity (Figure S3E), highlighting EZA's favorable safety profile.

IHC further demonstrated that EZA downregulated PD-L1, Foxp3, and Ki-67 expression while enhancing the infiltration of CD8$^+$ T cells, NK cells, and CD11c$^+$ dendritic cells (DCs). Elevated cleaved caspase-3 and TUNEL signals indicated increased tumor apoptosis, and macrophage polarization shifted toward the M1 phenotype with a concurrent decrease in the M2 population (Figure 3G). Together, these findings suggest that EZA suppresses CAC by reshaping the TIME by reducing PD-L1-mediated immunosuppression and promoting a cytotoxic T cell-driven antitumor response.

EZA promotes the ubiquitination-mediated degradation of PD-L1 by targeting β-TrCP

To elucidate the mechanism of EZA-induced PD-L1 downregulation, we performed transcriptome analysis on RKO cells treated with EZA, identifying 453 upregulated and 166 downregulated genes. Gene Ontology (GO) enrichment revealed a significant overrepresentation of ubiquitination-related pathways among these differentially expressed genes[34,35] (Figures 4A, 4B, and S5A). These results suggest that EZA might enhance PD-L1 degradation by modulating the UPS. Consistent with a post-translational mechanism, PD-L1 mRNA levels remained unchanged after EZA treatment (Figures S5B and S5C). Cycloheximide (CHX)-based protein stability assays in H1975 cells confirmed that EZA significantly accelerated PD-L1 turnover, shortening its half-life from 16.17 h (CHX alone) to 2.044 h (CHX+EZA) (Figures 4C and S5E). This trend was recapitulated in RKO cells (CHX: $t_{1/2}$ = 16.62 h; CHX + EZA: $t_{1/2}$ = 8.029 h) (Figure S5D). To identify the specific degradation pathway, H1975 and RKO cells were treated with EZA in combination with either the proteasome inhibitor MG132 or lysosomal/autophagy inhibitors such as chloroquine (CQ), bafilomycin A1, or 3-methyladenine. Western blot analysis revealed that only MG132 blocked EZA-induced PD-L1 degradation (Figures 4D and S5I–S5L), confirming UPS dependence. Immunoprecipitation (IP) assays directly demonstrated enhanced PD-L1-ubiquitin conjugation following EZA treatment (Figure 4E). Finally, flow

(H and I) (H) Representative tumor images and (I) corresponding growth curves from LLC-implanted nude mice treated with vehicle or EZA (50 mg/kg) (*n* = 5 mice per group).

(J and K) Flow cytometry analysis of immune cell subsets within the tumor immune microenvironment (TIME) of LLC tumors from C57BL/6J mice. Quantified populations include Tregs (CD4$^+$CD25$^+$Foxp3$^+$), granzyme B$^+$ cytotoxic cells, Gr-1$^+$ MDSCs, M1 macrophages (F4/80$^+$CD11b$^+$CD86$^+$/CD80$^+$), M2 macrophages (F4/80$^+$CD11b$^+$CD206$^+$), and NK cells (NK1.1$^+$), with quantitative results shown.

(L and M) Representative immunohistochemical staining and quantification of the TIME in LLC tumor sections from C57BL/6J mice, including PD-L1, CD8$^+$ T cells, Foxp3$^+$ Tregs, cleaved caspase-3, TUNEL, Ki-67, F4/80$^+$ macrophages, CD206$^+$ macrophages, CD11c$^+$ DCs, and NK1.1$^+$ cells. Scale bars, 100 μm.

Data in (B)–(D) and (I)–(N) are presented as mean ± SD. Statistical significance was determined by one-way ANOVA (B and J–M) or two-way ANOVA (C, D, and I). *$p < 0.05$, **$p < 0.01$, ***$p < 0.001$, ****$p < 0.0001$; ns, not significant. See also Figures S2–S4.

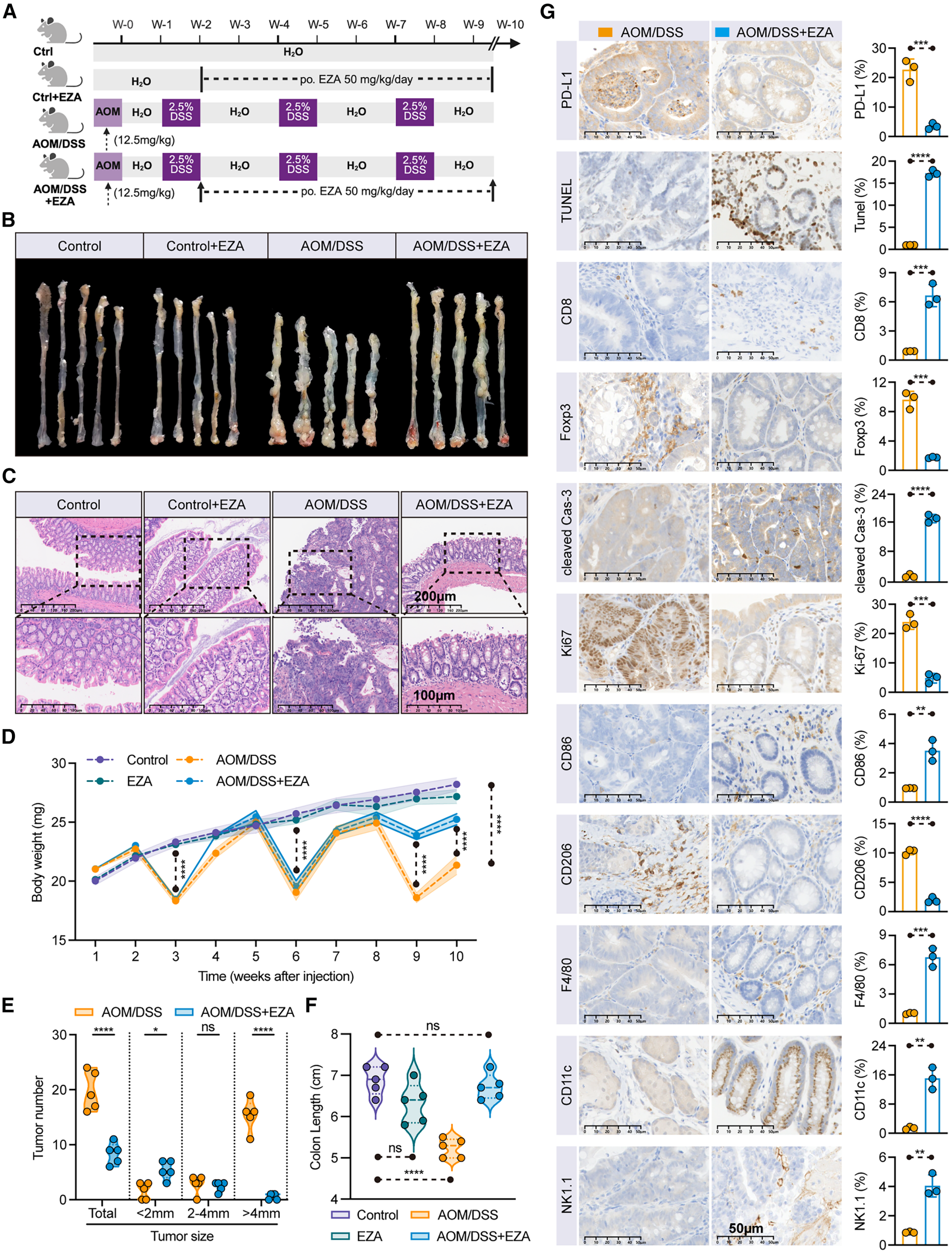

Figure 3. EZA can suppress AOM/DSS-induced colorectal cancer progression

(A) Schematic of the experimental design for the AOM/DSS-induced colorectal cancer model. Mice received a single intraperitoneal injection of AOM (12.5 mg/kg) on day 1, followed by three cycles of DSS treatment (2.5% in drinking water for 1 week, alternating with 2 weeks of normal water).
Mice were treated with vehicle or EZA (50 mg/kg) as indicated (*n* = 5 mice per group).
(B) Gross morphology of colorectal tumors from each experimental group.
(C) Representative hematoxylin and eosin (H&E)-stained sections of colorectal tissues Scale bars, 100 μm.
(D) Dynamic changes in body weight across the experimental groups.

(legend continued on next page)

cytometry and IF confirmed that EZA promoted PD-L1 degradation specifically via the proteasome pathway (Figures 4F, 4G, and S5M–S5Q). *In vivo*, EZA treatment increased overall protein ubiquitination in LLC and MC38 tumors (Figures 4H and S5F–S5H), supporting its role in promoting PD-L1 ubiquitination. In summary, EZA facilitates PD-L1 ubiquitination, thereby targeting it for proteasomal degradation and reducing its protein levels.

The UPS serves as the primary pathway regulating intracellular PD-L1 turnover.[36,37] This process relies on an enzymatic cascade involving E1, E2, and E3 enzymes, with E3 ubiquitin ligases conferring substrate specificity.[36,37] Several E3 ligases and deubiquitinating enzymes, including β-TrCP, HRD1, ARIH1, SPOP, STUB1, MARCH8, and CSN5, have been implicated in PD-L1 degradation.[31,38–45] To identify the mediators of EZA's effect on PD-L1, we performed small interfering RNA (siRNA)-mediated knockdown of these candidates in RKO cells. Only β-TrCP knockdown blocked EZA-induced PD-L1 degradation, pinpointing β-TrCP as the key mediator (Figures 4I and S6A–S6G). Furthermore, the endogenous interaction between PD-L1 and β-TrCP was confirmed by co-immunoprecipitation (coIP) (Figures 4J and 4K). To determine whether EZA directly binds β-TrCP, we performed a cellular thermal shift assay.[46] EZA (100 μM) significantly stabilized β-TrCP at 46°C, 49°C, and 52°C, indicating direct engagement (Figure 4L). Moreover, when lysates were incubated with increasing EZA concentrations at 46°C, β-TrCP stability increased in a dose-dependent manner (Figure 4M). Likewise, pronase digestion at a fixed ratio (1:300) showed enhanced stabilization of β-TrCP with higher EZA concentration (Figure 4N). Coimmunofluorescence analysis revealed that β-TrCP knockdown reduced its colocalization with PD-L1 (Figure 4O), whereas β-TrCP overexpression enhanced colocalization. Interestingly, EZA treatment further increased PD-L1/β-TrCP colocalization, demonstrating that EZA augments β-TrCP's recruitment to PD-L1. Molecular docking was employed to predict potential binding interfaces between EZA and β-TrCP. Analysis of top-ranking poses suggested that EZA might engage with a pocket involving residues Leu347, Leu387, and Arg560 (Figures 4P–4R). To experimentally validate these predictions, we performed site-directed mutagenesis on these residues. Subsequent binding affinity measurements via microscale thermophoresis in 293T cells revealed high-affinity interaction between EZA and wild-type (WT) β-TrCP (K_d = 1.25 μM). This binding was essentially abolished with the Leu387A mutant, whereas mutations at Leu347 (K_d = 11.7 μM) and Arg560 (K_d = 13.2 μM) resulted in a less-pronounced reduction in affinity (Figure 4S). This identifies Leu387 as the critical residue for the EZA-β-TrCP interaction. In addition, EZA significantly upregulated β-TrCP mRNA levels after 12 h (Figures S6I and S6J), slowed β-TrCP degradation in CHX-chase assays (Figure S6K), and elevated β-TrCP protein levels in a dose- and time-dependent manner (Figures S6L–S6M). These data collectively demonstrate that EZA both binds to and stabilizes β-TrCP, enhancing its expression.

EZA induces β-TrCP-mediated, phosphorylation-dependent K48-linked ubiquitination and degradation of PD-L1

A previous study revealed that glycogen synthase kinase-3β (GSK3β) phosphorylates PD-L1 at Thr180 and Ser184, creating a phosphodegron recognized by β-TrCP.[38] To evaluate whether this modification is required for the action of EZA, we generated a PD-L1 T180A/S184A double mutant and measured its stability in 293T cells by CHX chase assays. EZA treatment markedly reduced the half-life of WT-PD-L1 (from 33.22 to 7.213 h) but had no significant effect on the T180A/S184A mutant (from 33.14 to 31.38 h) (Figures 5A and 5B). Moreover, IF analysis revealed that EZA enhanced colocalization of β-TrCP with WT-PD-L1 in β-TrCP-overexpressing cells but failed to promote interaction with the T180A/S184A mutant (Figure 5C). Consistently, coIP revealed that EZA increased β-TrCP binding to WT-PD-L1, whereas the T180A/S184A mutation significantly impaired this interaction (Figure 5D). These data indicate that Thr180/Ser184 (T180/S184) phosphorylation is indispensable for EZA-mediated PD-L1 turnover through β-TrCP. To evaluate the functional relevance of PD-L1 phosphorylation, we cocultured RKO cells expressing either WT or T180A/S184A PD-L1 with Jurkat T cells. As shown in Figures 5E–5G, T180A/S184A mutation significantly attenuated EZA-induced T cell cytotoxicity compared with WT PD-L1, demonstrating that T180/S184 phosphorylation is essential for EZA-mediated immune-potentiating effect. To further functionally validate whether EZA exerts antitumor effects via β-TrCP-mediated PD-L1 degradation, we cocultured PD-L1- or β-TrCP-knockdown RKO cells with PD-1-overexpressing Jurkat cells. As expected, PD-L1 knockdown enhanced T cell cytotoxicity comparably with EZA (Figures 5H and 5K). Crucially, β-TrCP knockdown completely abolished EZA-driven T cell cytotoxicity (Figures 5I and 5K), whereas β-TrCP overexpression did not augment it further (Figures 5J and 5K). Conclusively, these findings demonstrate that EZA targets β-TrCP to promote PD-L1 ubiquitination and degradation, thereby relieving PD-L1/PD-1-mediated immunosuppression and enhancing T cell antitumor activity.

K48- and K63-linked polyubiquitin chains constitute the two most abundant modifications in the UPS, which predominantly function in the ubiquitin-proteasome degradation pathway.[4,47] To determine how EZA mediates PD-L1 degradation through the UPS by targeting specific proteins, we performed quantitative proteomics on RKO cells treated with 40 μM EZA for 24 h. Differential expression profiling revealed several key regulators (e.g., RNF6,[48] IRF3,[49] and TOLLIP[50]) associated with K48-specific polyubiquitin assembly and proteasomal targeting

(E) Quantification of tumor burden, including total tumor number and tumor size per mouse.
(F) Measurement of colon length at the experimental endpoint.
(G) Immunohistochemical analysis of the TIME, including staining and quantification of PD-L1, $CD8^+$ cytotoxic T cells, $Foxp3^+$ Tregs, cleaved caspase-3, TUNEL, Ki-67, $CD86^+$, $F4/80^+$, $CD206^+$ macrophages, $CD11c^+$ DCs, and $NK1.1^+$ cells. Scale bar, 50 μm.
Data in (D)–(G) are presented as mean ± SD.
Statistical significance was determined by one-way ANOVA (F and G) or two-way ANOVA (D and E). $^*p < 0.05$, $^{**}p < 0.01$, $^{***}p < 0.001$, $^{****}p < 0.0001$; ns, not significant. See also Figure S3.

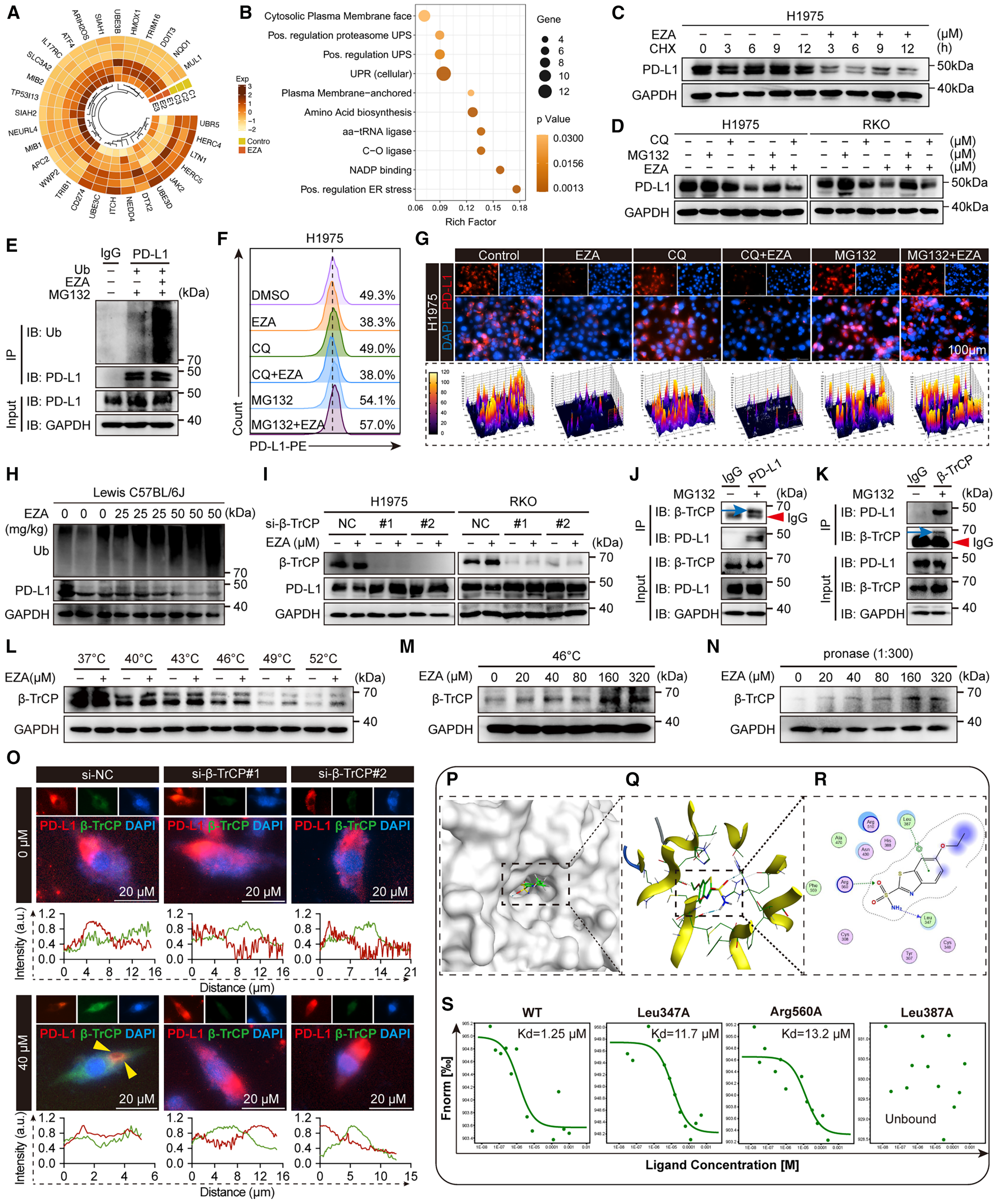

Figure 4. EZA promotes the ubiquitination-mediated degradation of PD-L1 by targeting β-TrCP

(A) Heatmap of differentially expressed genes in RKO cells treated with DMSO or 40 μM EZA for 24 h. The color gradient represents the magnitude of the gene expression changes.

(B) Top 10 significantly enriched GO pathways. pos., positive; UPS, ubiquitin-proteasome system; ER, endoplasmic reticulum; UPR, unfolded protein response.

(legend continued on next page)

(Figure 5L). GO and Kyoto Encyclopedia of Genes and Genomes (KEGG) enrichment analysis further underscored the involvement of protein polyubiquitination and proteasome pathways (e.g., GO:0070936) (Figures 5M and 5N). In ubiquitin mutation assays, EZA markedly increased PD-L1 polyubiquitination in cells expressing WT-Ub or K63R-Ub, but not in cells expressing K48R-Ub (Figure 5O). These results demonstrate that EZA specifically induces K48-linked polyubiquitination to target PD-L1 for degradation. Collectively, our data demonstrate that EZA binds to and stabilizes β-TrCP, thereby facilitating its recruitment to phosphorylated PD-L1. This enhanced recruitment drives K48-linked polyubiquitination and subsequent proteasome-mediated degradation of PD-L1.

EZA exhibits PD-L1 blockade-like antitumor activity and exerts synergistic effects with anti-CTLA-4

Although cytotoxic T-lymphocyte antigen 4 (CTLA-4) blockade has established clinical benefits in cancer immunotherapy, its efficacy as a monotherapy is limited by suboptimal response rates and significant irAEs.[51–53] We hypothesized that combining EZA with CTLA-4 blockade could potentiate antitumor efficacy through synergistic mechanisms. To test this, C57BL/6J mice bearing LLC or MC38 tumors received EZA monotherapy, anti-PD-L1 monotherapy, anti-CTLA-4 monotherapy, or their combinations (Figures 6A–6C and S8A–S8D). All treatments significantly inhibited tumor growth; notably, EZA alone showed efficacy comparable to that of the other antibody monotherapies. The EZA and anti-CTLA-4 combination achieved antitumor activity comparable to the combination of anti-PD-L1 and anti-CTLA-4 (Figures 6A, 6C, and S7A). Notably, the lack of further enhancement beyond PD-L1 blockade suggests that EZA's effects are primarily mediated through PD-L1 downregulation. All regimens were well tolerated, with no significant weight loss or organ toxicity observed (Figures S8C, S11A, and S11B).

Next, multicolor flow cytometry of LLC tumor-infiltrating lymphocytes revealed that EZA and anti-CTLA-4, compared with monotherapies, more profoundly reduced the immunosuppressive Tregs ($CD4^+CD25^+Foxp3^+$) and activated MDSCs ($Gr\text{-}1^+$). Concurrently, this combination synergistically amplified cytotoxic immunity, as evidenced by the expansion of granzyme B^+ effector cells, NK cells, and $CD19^+$ B cells, coupled with macrophage repolarization toward the proinflammatory M1 phenotype ($CD86^+/CD80^+$) and suppression of the tumor-promoting M2 transition ($CD206^+$) (Figures 6D and S7B). IHC confirmed these immune shifts and showed marked upregulation of the E3 ligase β-TrCP in tumor tissues (Figures 6E and S7C). Similar immunophenotypic changes were observed in MC38 tumors by flow cytometry (Figures S8E and S9A) and IHC (Figure S10A), reinforcing that EZA promotes ubiquitination-mediated PD-L1 degradation. Overall, EZA not only matches anti-PD-L1 monotherapy in efficacy but also synergizes with anti-CTLA-4 to reprogram the immunosuppressive TIME, enabling multifaceted immune potentiation.

High PD-L1 with low β-TrCP predicts improved response and survival in NSCLC patients receiving neoadjuvant immunotherapy

To define the expression patterns of β-TrCP and PD-L1 in tumors and their associations with prognosis and treatment response, we analyzed public The Cancer Genome Atlas (TCGA) and GEO datasets. Notably, Kaplan-Meier survival curves revealed that high β-TrCP and low PD-L1 expression was significantly associated with prolonged overall survival (OS) in lung

(C) Western blot analysis of PD-L1 protein levels in H1975 cells treated with cycloheximide ([CHX], 50 μg/mL) for the indicated times, with or without 12-h pretreatment with 40 μM EZA.
(D) Western blot analysis of PD-L1 in H1975 and RKO cells cotreated with EZA (40 μM, 12 h) and either proteasome inhibitor MG132 (5 μM, 6 h) or lysosome inhibitor chloroquine (CQ, 40 μM, 12 h).
(E) Co-immunoprecipitation (coIP) of PD-L1 from RKO cells overexpressing hemagglutinin-tagged ubiquitin, followed by immunoblotting with an anti-ubiquitin antibody to assess PD-L1 ubiquitination levels upon EZA treatment (40 μM, 6 h).
(F) Flow cytometry analysis of cell-surface PD-L1 expression in H1975 cells co-treated with EZA and MG132 or CQ as in (D).
(G) Representative immunofluorescence (IF) images showing membrane PD-L1 (red) in H1975 cells treated as in (D). A 2.5D reconstruction (bottom row) quantifies the fluorescence intensity distribution. Scale bar, 100 μm.
(H) Western blot of ubiquitinated PD-L1 in the LLC subcutaneous tumors from mice treated with vehicle or EZA (25 or 50 mg/kg).
(I) Western blot analysis of β-TrCP and PD-L1 expression in H1975 and RKO cells transfected with siRNA targeting β-TrCP (si-β-TrCP) or non-targeting control (si-NC).
(J and K) CoIP assays in MG132-treated RKO cells using PD-L1 antibody (J) or β-TrCP antibody (K), followed by immunoblotting to assess the PD-L1/β-TrCP interaction upon EZA treatment (40 μM, 6 h).
(L) Cellular thermal shift assay (CETSA) assessing β-TrCP thermal stability in cell lysates incubated with DMSO or 40 μM EZA across a temperature gradient (37°C–52°C).
(M) Effect of increasing EZA concentrations on β-TrCP stability at 46°C.
(N) Dose-dependent stabilization of β-TrCP by EZA at a protease/protein ratio of 1:300.
(O) Representative IF images showing the subcellular localization of PD-L1 (red) and β-TrCP (green) in RKO cells transfected with si-β-TrCP or si-NC for 48 h, followed by treatment with 40 μM EZA for 6 h. Colocalization was quantified through fluorescence intensity profiling. Yellow arrows denote representative co-localized regions. Scale bars, 20 μm.
(P–S) (P) Predicted binding pose of EZA (cyan sticks) within the surface model of β-TrCP. (Q) Interaction analysis, including hydrogen bonds and hydrophobic contacts. (R) Predicted key binding residues: Leu347 (hydrophobic, backbone donor), Leu387 (hydrophobic, solvent-exposed, arene-H interaction), and Arg560 (basic, side-chain donor). (S) Microscale thermophoresis (MST) measurement of the binding affinity (K_d) of EZA for WT vs. mutant (L347A, L387A, and R560A) GFP-tagged β-TrCP overexpressed in 293T cells.
Data are presented as mean ± SD from three independent experiments. *$p < 0.05$, **$p < 0.01$, ***$p < 0.001$, ****$p < 0.0001$; ns, not significant. See also Figures S5 and S6.

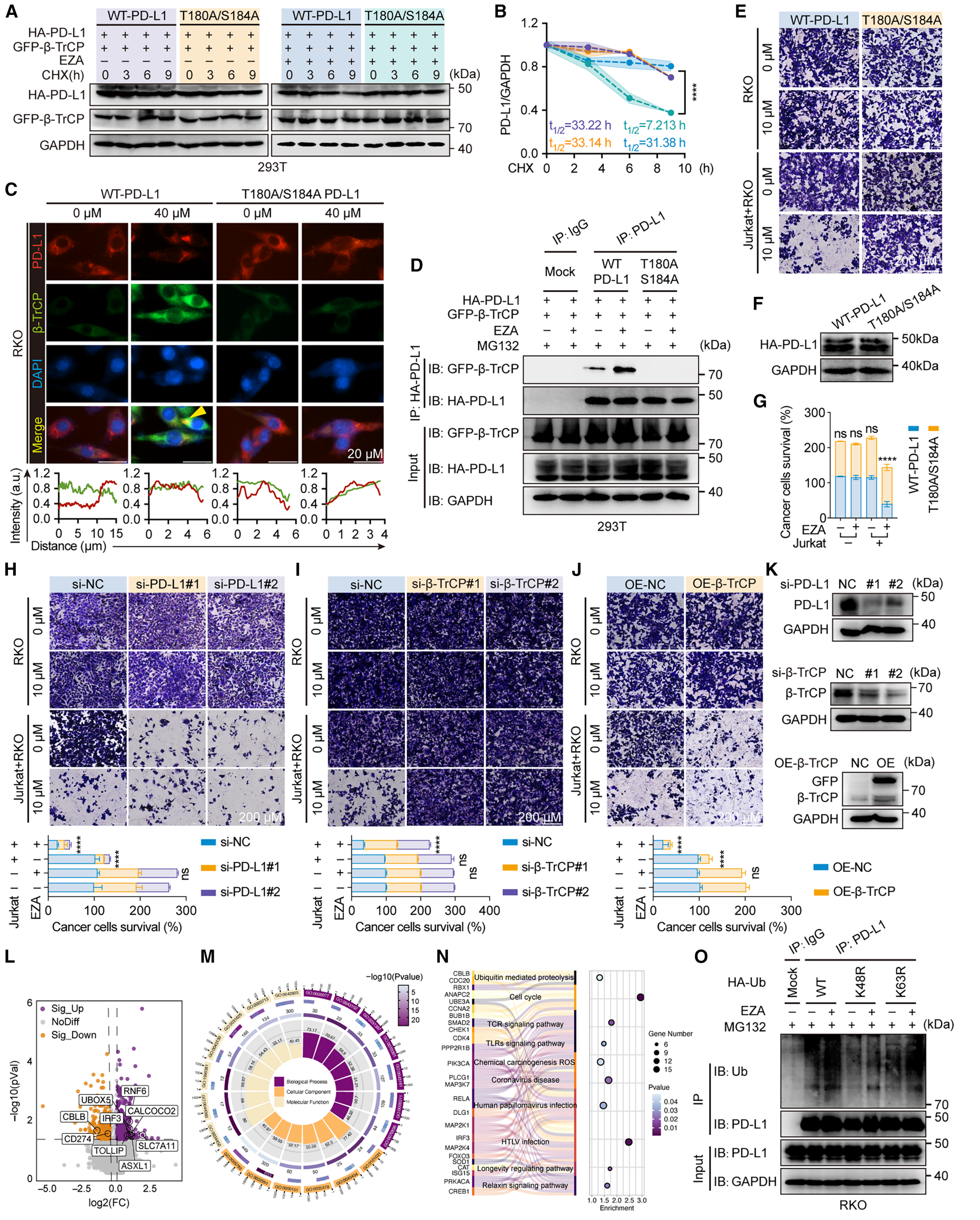

(legend on next page)

CellPress
OPEN ACCESS

adenocarcinoma (LUAD) and colon adenocarcinoma (COAD) cohorts, thereby identifying this combination as a putative favorable prognostic biomarker (Figures S12A–S12D). However, among patients receiving anti-PD-1 therapy, this trend reversed: those with high β-TrCP and low PD-L1 expression experienced poorer survival (Figures S12E and S12F), indicating that the interplay between β-TrCP and PD-L1 critically influences immunotherapy outcomes.

We subsequently examined tumor samples from 32 locally advanced NSCLC patients with LUAD and lung squamous cell carcinoma (LUSC) who received neoadjuvant chemoimmunotherapy (NICT). The cohort comprised 72% LUSC and 72% stage I–III patients, with 50% achieving major pathological response (MPR) or complete pathological response (pCR) (Figures 7A and 7B; Table S1). Statistical analysis revealed that biological sex had no significant impact on the therapeutic response or clinical outcomes. Computed tomography (CT) imaging showed significantly greater tumor reduction in responders vs. non-responders ($p = 0.024$) (Figures 7A and 7C). Consistent with the database analysis, IF revealed higher PD-L1 and lower β-TrCP expression in responders (Figures 7D and 7E), confirming their inverse relationship. Furthermore, survival analysis revealed that patients with high PD-L1/low β-TrCP expression had superior OS (36.2 ± 13.5 vs. 22.5 ± 13.1 months) and progression-free survival (PFS) (36.4 ± 13.8 vs. 18.9 ± 14.2 months) (Figures 7F and 7G). These results suggest that β-TrCP and PD-L1 expression levels are associated with NICT benefit in this cohort, although prospective validation in larger independent cohorts is needed.

To explore the role of β-TrCP within the TIME, we assessed immune infiltration in the TCGA-COAD cohort ($N = 282$). β-TrCP expression correlated positively with the stromal score (Pearson $R = 0.34$, $p < 0.0001$) and overall immune infiltration score ($R = 0.57$, $p < 0.0001$), mirroring the PD-L1 pattern (Figures S12G and S12H). β-TrCP also positively correlated with the infiltration of B cells, $CD8^+$ T cells, macrophages, and DCs (Figure S12I), suggesting a role in recruiting immune cells. Finally, across integrated TCGA, GTEx, and TARGET datasets, β-TrCP expression showed significant positive correlations ($p < 0.05$) with 150 immune-related genes (Figure S12J), including chemokines (e.g., CXCL12 and CCL16), chemokine receptors (e.g., CCR5 and CXCR3), antigen-presentation molecules (e.g., TAP1, TAP2, and HLA-DOB), and costimulatory immune regulators (e.g., CD40, CD80, and IL6R). Taken together, these findings suggest that β-TrCP not only regulates PD-L1 expression but also orchestrates TIME remodeling, thereby influencing tumor immunogenicity and immunotherapy response.

DISCUSSION

ICT targeting PD-1/PD-L1 has revolutionized cancer treatment.[1,2] However, its clinical benefits remain limited, with objective response rates of only 20%–30% and frequent acquired resistance.[6,7] Promoting PD-L1 protein degradation thus represents a promising therapeutic strategy.[10,11] Here, we identified EZA, a classical CAI, as a small-molecule inducer of PD-L1 degradation with antitumor activity. Mechanistically, EZA induces β-TrCP-mediated PD-L1 phosphorylation and K48-linked ubiquitination, driving proteasomal degradation to reverse T cell immunosuppression. EZA sensitizes lung and CRC cells to T cell-mediated killing, enhances $CD8^+$ T cell infiltration, and reduces MDSCs and Tregs in subcutaneous tumor and AOM/DSS-induced CAC models. Moreover, EZA synergizes with anti-CTLA-4 to further activate the TIME. In our clinical cohort, high PD-L1 with low β-TrCP levels correlated with improved response and survival in NSCLC patients receiving NICT, suggesting a potential prognostic association that requires validation in larger, independent studies. These findings indicate that EZA is a PD-L1 degrader with antitumor activity and immunomodulatory capacity, suggesting a potential strategy for cancer therapy.

Classic CAIs like EZA are thought to primarily regulate the pH and metabolic adaptation of the microenvironment by inhibiting

Figure 5. EZA induces β-TrCP-mediated, phosphorylation-dependent K48-linked ubiquitination and degradation of PD-L1

(A) Western blot analysis of PD-L1 protein levels and half-life under CHX (50 μg/mL) time-gradient treatment in RKO cells transfected with WT-PD-L1 or the T180A/S184A mutant, with or without pre-treatment with 40 μM EZA for 9 h.

(B) Quantification of the PD-L1 and β-TrCP half-lives corresponding to (A).

(C) Immunofluorescence of PD-L1 (red) and β-TrCP (green) colocalization in EZA-treated RKO cells expressing either WT or T180A/S184A PD-L1 and treated with 40 μM EZA for 12 h. Colocalization was quantified through fluorescence intensity profiling. Yellow arrows denote representative colocalized regions. Scale bar, 20 μm.

(D) CoIP assay in 293T cells co-expressing GFP-β-TrCP and either WT or T180A/S184A PD-L1. Cells were treated with 40 μM EZA or 5 μM MG132 for 6 h. Interaction was analyzed by IP using anti-PD-L1 antibody followed by immunoblotting.

(E) Crystal violet staining of RKO cells transfected with WT or T180A/S184A PD-L1, following a 48-h coculture with PD-1-overexpressing Jurkat T cells in the presence or absence of 40 μM EZA. Scale bar, 200 μm.

(F) Western blot validating the overexpression of WT and T180A/S184A PD-L1 in RKO cells.

(G) Quantification of residual tumor cells from the coculture assay shown in (E).

Data are normalized to the corresponding tumor cell-only control group.

(H–J) Crystal violet staining and quantification of residual tumor cells from coculture assays. RKO cells were transfected with si-PD-L1 (H), si-β-TrCP (I), or OE-β-TrCP (J) and then cocultured with PD-1-overexpressing Jurkat T cells for 48 h ±40 μM EZA. Scale bars, 200 μm.

(K) Western blot confirming the knockdown or overexpression efficiency of PD-L1 and β-TrCP in the samples used for coculture assays (H–J).

(L–N) Quantitative proteomic analysis of RKO cells treated with DMSO or 40 μM EZA for 24 h ($n = 3$ biological replicates per group). (L) Volcano plot of differentially expressed proteins. (M) Top 20 enriched GO biological process terms. (N) Top 20 enriched KEGG pathways.

(O) Ubiquitination profiling of RKO cells transfected with WT-Ub, K48R-Ub, or K63R-Ub following 40 μM EZA and 5 μM MG132 treatment for 6 h. PD-L1 immunoprecipitates were probed for chain-specific ubiquitination.

Data in (A) and (G)–(J) are presented as mean ± SD from three independent experiments. Statistical significance was determined by two-way ANOVA (A and G–J). *$p < 0.05$, **$p < 0.01$, ***$p < 0.001$, ****$p < 0.0001$; ns, not significant. See also Figure S6.

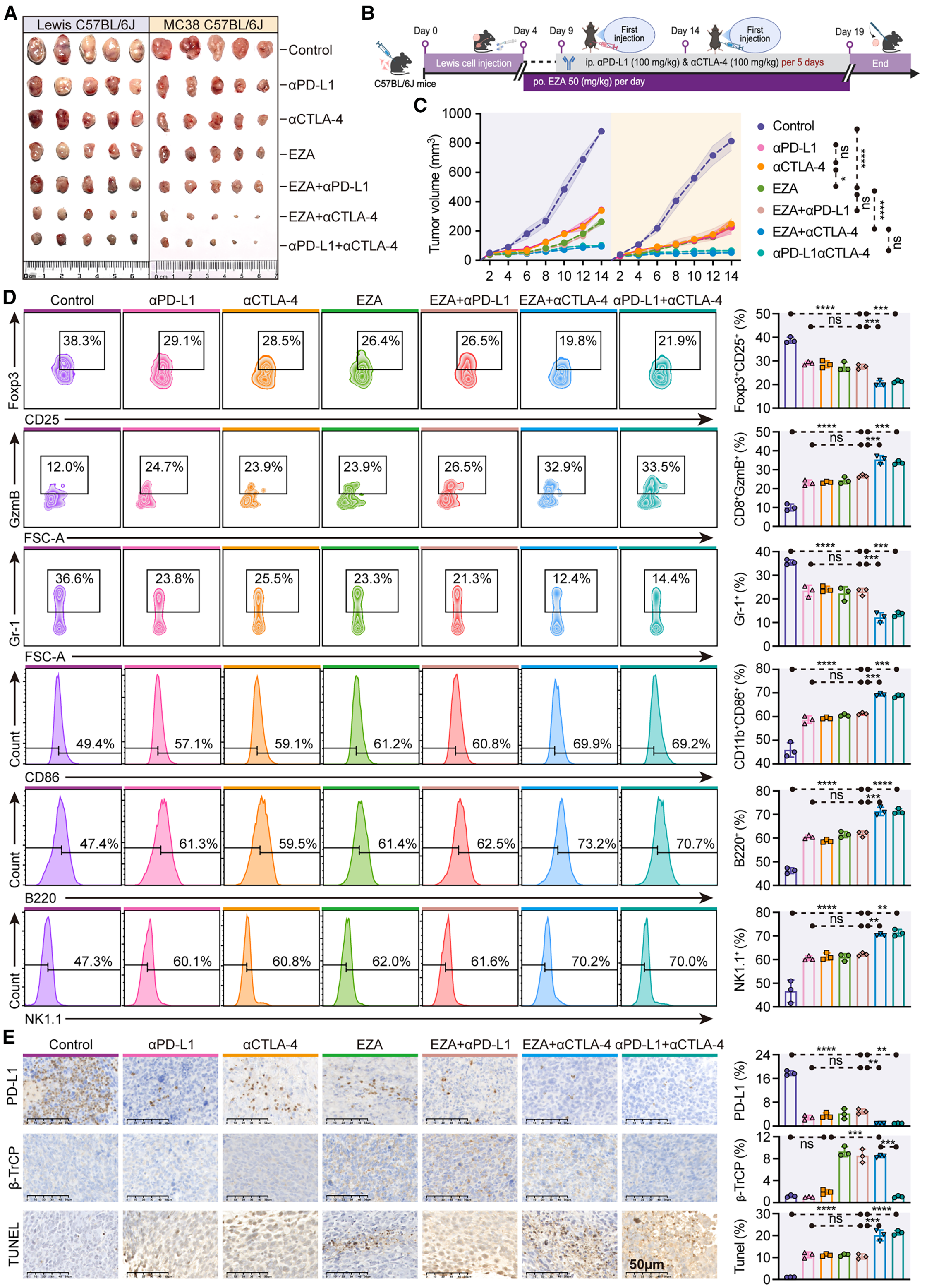

Figure 6. EZA exhibits PD-L1 blockade-like antitumor activity and exerts synergistic effects with anti-CTLA-4

(A) Representative images of excised tumors from C57BL/6J mice bearing Lewis lung carcinoma (LLC) or MC38 tumors after treatment with vehicle, EZA (50 mg/kg), anti-PD-L1 (100 μg/mouse), anti-CTLA-4 (100 μg/mouse), or their combinations (n = 5 mice per group).

(B) Schematic diagram of the experimental design.

(legend continued on next page)

CA IX/XII.[22,54–57] However, emerging evidence suggests broader immunomodulatory potential of CAIs, including influencing macrophage polarization and immune cell infiltration.[22,58] Whether CAIs directly target immune checkpoints like PD-L1 was unclear.[54–56] Our study shows that EZA binds β-TrCP to promote PD-L1 phosphorylation at T180/S184, triggering K48-linked polyubiquitination and proteasomal degradation. This mechanism overcomes PD-L1-mediated immune evasion and positions CAIs as a class of immune checkpoint modulators. Across lung cancer, CRC, and AOM/DSS-induced CAC models, EZA reshaped the TIME by promoting the infiltration of $CD8^+$ T cells, B cells, NK cells, DCs, and M1 macrophages, while suppressing MDSCs, Tregs, and M2 macrophages. Thus, EZA exerts antitumor effects by alleviating immunosuppression and activating antitumor immunity, providing a rationale for repurposing EZA and a framework for developing other PD-L1 degraders.

The UPS, which mediates substrate-specific degradation through the E1-E2-E3 enzyme cascade, is central to protein homeostasis and immune responses.[13,14,16,59] Its dysregulation promotes tumorigenesis, metastasis, and therapy resistance.[4] Small molecules targeting UPS components, such as E3 ligases or deubiquitinases, are promising antitumor agents.[38–40,42,45,60] For instance, PROTACs exploit E3 ligases CRBN and VHL to degrade oncoproteins such as IKZF1/3 and HIF-1α, respectively, whereas MDM2 inhibitors (e.g., Nutlin-3) activate tumor-suppressive pathways by blocking p53 ubiquitination.[61–65] β-TrCP, an SCF adaptor, regulates the stability of key proteins (e.g., cyclin E, Wee1, and IκBα) by recognizing phosphodegron motifs, thereby modulating cell cycle progression, DNA damage response, and NF-κB signaling.[66–69] β-TrCP dysfunction or mutation is closely linked to cancer progression.[38,67] Our study revealed that EZA specifically binds the Leu387 residue of β-TrCP, triggering K48-linked ubiquitination and proteasomal degradation of PD-L1. Notably, EZA exhibits favorable membrane permeability and pharmacokinetics, enabling efficient β-TrCP targeting and enhanced PD-L1 interaction, thereby overcoming the limitations of antibody therapies.[20] These findings elucidate a mechanism of immune checkpoint regulation by EZA and may inform the development of E3 ligase-based small-molecule degraders against membrane proteins like PD-L1.

Our study highlights the clinical translational potential of EZA via β-TrCP-mediated PD-L1 degradation. While PD-L1 antibodies improve NSCLC survival, approximately 40%–60% PD-L1-positive patients show primary resistance and up to 31.2% experience grade 3–5 irAEs.[4,70] By degrading PD-L1 rather than merely blocking PD-1/PD-L1 interactions, EZA achieved an 89.13% complete tumor regression rate in LLC models with low toxicity, addressing key issues of resistance and safety. Moreover, EZA synergizes with anti-CTLA-4, matching the efficacy of combined antibody therapy. Its action of PD-L1 degradation, oral bioavailability, and safety profile in preclinical models may warrant further clinical evaluation. Notably, the AOM/DSS model suggests the potential of the EZA for early intervention in inflammation-driven tumors, aligning with reports of aberrant PD-L1 in ulcerative CAC.[71] Crucially, high PD-L1/low β-TrCP patients had longer OS (MPR + pCR: 36.2 ± 13.5 months vs. non-MPR: 22.5 ± 13.1 months), consistent with trials such as CheckMate 816 and KEYNOTE-671.[72,73] β-TrCP may warrant further evaluation as a candidate stratification marker, pending validation in prospective studies. A companion diagnostic system based on the dynamic β-TrCP/PD-L1 ratio could be explored in future studies. This strategy leverages two key attributes of EZA: first, its favorable oral absorption profile—characterized by high solubility, efficient intestinal epithelial permeability *in vitro*, and rapid systemic exposure (Tmax = 0.17 h in mice) *in vivo*[24,74]—and second, its capacity to reprogram TIME through β-TrCP targeting. By integrating these properties, the diagnostic approach is designed to accelerate the translation of mechanistic insights into clinical benefits.

In summary, our findings indicate that EZA reduces PD-L1 levels and reverses immunosuppression via a β-TrCP-mediated phosphorylation-UPS pathway. These findings offer a strategy to overcome the limitations of ICTs and may inform patient stratification strategies for personalized immunotherapy.

Limitations of the study

Despite the positive implications of these findings, several limitations of the present study warrant further consideration. While EZA demonstrated a favorable safety profile in murine models, its long-term impact on PD-L1-expressing physiological tissues necessitates comprehensive evaluation through systematic pharmacokinetic and pharmacodynamic studies to establish a precise clinical dosing window. Furthermore, although we have validated that EZA achieves PD-L1 degradation efficiency comparable to the heterobifunctional PROTAC degrader PA8, fundamental pharmacological distinctions between these two modalities remain. Specifically, while PROTACs typically operate via sub-stoichiometric catalytic turnover, EZA's monovalent modulation of the β-TrCP E3 ligase may involve distinct binding kinetics and degradation scales that require more exhaustive kinetic profiling across diverse cellular contexts. Additionally, the potential off-target effects of EZA beyond its established targets, alongside its broader impact on the immune checkpoint landscape, remain to be fully elucidated. Last, the clinical correlation among β-TrCP, PD-L1, and immunotherapy response was derived from a single cohort without external validation. Consequently, the clinical utility of these biomarkers must be further established in larger, multi-center trials. Due to the modest

(C) Tumor growth curves of LLC and MC38 models during the treatment period.

(D) Flow cytometric analysis and quantification of immune cell subsets from LLC tumors, including $Foxp3^+$ Tregs, $Gr\text{-}1^+$ MDSCs, granzyme B^+ cytotoxic cells, B cells ($B220^+$), $NK1.1^+$ cells, and M1 macrophages ($F4/80^+CD11b^+CD86^+$).

(E) Immunohistochemical analysis of PD-L1, β-TrCP, and TUNEL expression in tumor tissues. Scale bar, 50 μm.

Data in (C)–(E) are presented as mean ± SD from three independent experiments. Statistical significance was determined by one-way ANOVA (D and E) or two-way ANOVA (C). *$p < 0.05$, **$p < 0.01$, ***$p < 0.001$, ****$p < 0.0001$; ns, not significant. See also Figures S7–S11.

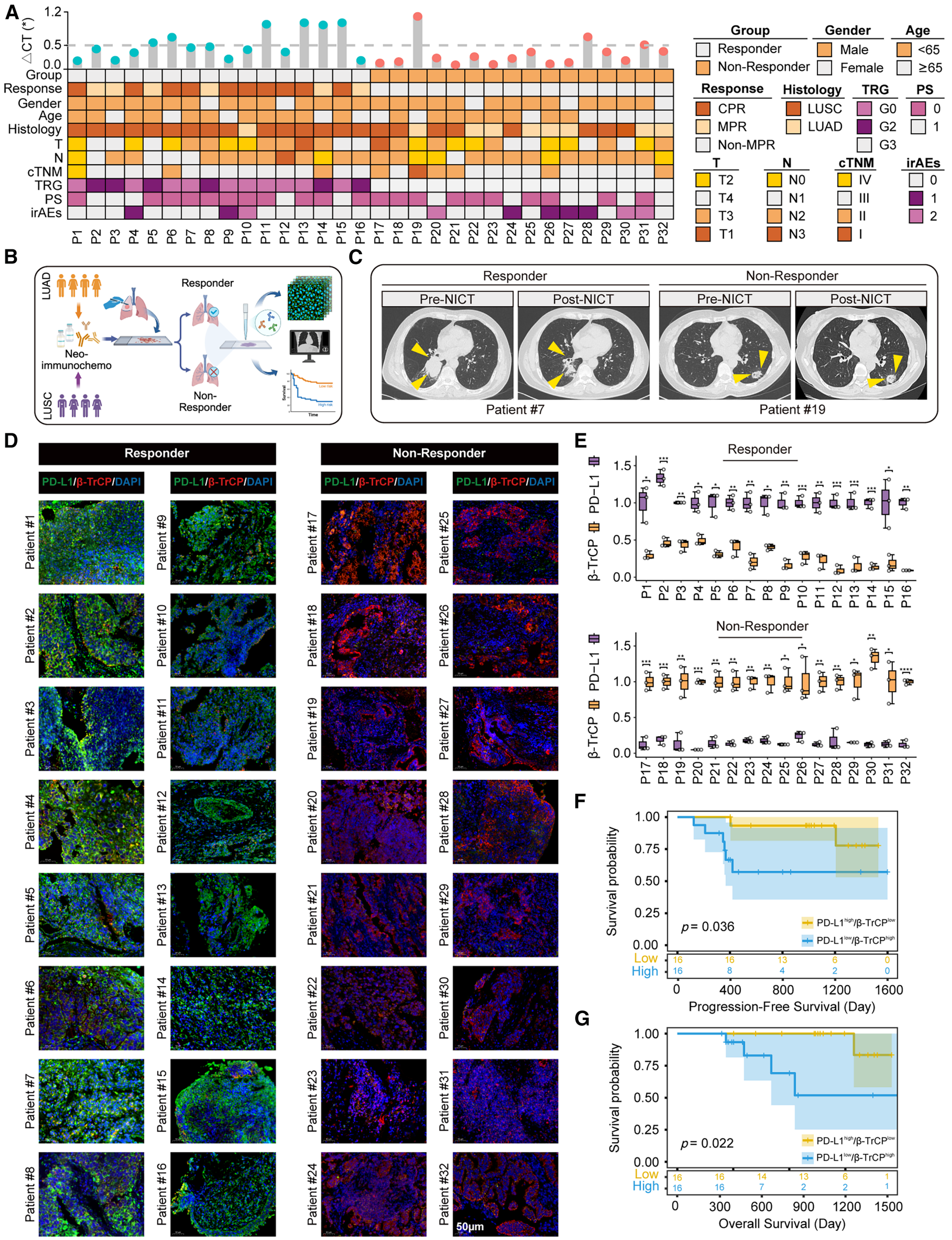

Figure 7. High PD-L1 with low β-TrCP predicts improved response and survival in NSCLC patients receiving neoadjuvant immunotherapy

(A) Baseline characteristics and changes in the maximal tumor diameter assessed by CT imaging before and after neoadjuvant immunochemotherapy (NICT) in 32 patients with locally advanced NSCLC.

(B) Flowchart of patient enrollment and selection for the clinical cohort.

(C) Representative axial CT images from one responder (left) and one non-responder (right) showing tumor lesions before and after NICT. The maximal tumor diameter is indicated by yellow arrows.

(legend continued on next page)

size of this patient cohort, our preliminary observation that biological sex had no significant confounding effect may lack the statistical power to capture subtle, sex-specific variations in antitumor immunity. Future validation in a larger, multi-center, and more gender-balanced population is required to ensure the generalizability of these findings across diverse demographic groups.

RESOURCE AVAILABILITY

Lead contact

Requests for further information and resources should be directed to and will be fulfilled by the lead contact, Xinying Xue (xuexinying@xwhosp.org).

Materials availability

This study did not generate reagents.

Data and code availability

- RNA-seq data have been deposited in the GEO database under accession number GSE318640 (https://www.ncbi.nlm.nih.gov/geo/query/acc.cgi?acc=GSE318640) and are publicly available as of the date of publication. Proteomics data have been deposited in the PRIDE (ProteomeXchange) database under accession number PXD074302 (https://www.ebi.ac.uk/pride/archive/projects/PXD074302) and are publicly available as of the date of publication. All other data are available in the main text or the supplementary materials.
- This paper does not report original code.
- Any additional information required to reanalyze the data reported in this paper is available from the lead contact upon request.

ACKNOWLEDGMENTS

This study was supported by the Beijing Peaking Talent Support Program (DFL20240703), the Medical High-level Talent Program - National Outstanding Young Physician (2-2-008-0247), the Capital Medical University Outstanding Young Talent Program (A2310), the National Natural Science Foundation of China (62176166, 82374086, 82574633, 82430119, and 82104459), the Postdoctoral Innovation Talents Support Program (BX20240234), State Key Laboratory of Drug Research (SKLDR-2025-KF-01), National Key Research and Development Program of China (2022YFC3502000), Shanghai Municipal Science and Technology Major Project (ZD2021CY001), Three-year Action Plan for Shanghai TCM Development and Inheritance Program (ZY (2021-2023)-0401), Innovation Team and Talents Cultivation Program of National Administration of Traditional Chinese Medicine (ZYYCXTDD-202004), Science and Technology Commission of Shanghai Municipality (20YF1458700), Organizational Key Research and Development Program of Shanghai University of Traditional Chinese Medicine (2023YZZ02), CAMS Innovation Fund for Medical Sciences (CIFMS) (2023-I2M-3-009), and Key Project at Central Government Level: The Ability Establishment of Sustainable Use for Valuable Chinese Medicine Resources (2060302-2305-02). We thank the staff members of the Large-scale Protein Preparation System at the National Facility for Protein Science in Shanghai for providing technical support and assistance in data collection and analysis.

AUTHOR CONTRIBUTIONS

X.X., S. Liu, and W.Z., conceptualization, original draft, methodology, review and editing, funding acquisition, and supervision; X. Lin., Q.W., and M. Xu, analyzed the data, carried out the experiments, generated the figures, and wrote the paper; D.Y., H.H., Q.Z., J.Y., M. Xie, H.X., X.Z., J.L., Y.C., L.L., X.T., X.L., S. Li, S.X., and Y.T. participated in part of the experiments.

DECLARATION OF INTERESTS

The authors declare no competing interests.

STAR★METHODS

Detailed methods are provided in the online version of this paper and include the following:

- KEY RESOURCES TABLE
- EXPERIMENTAL MODEL AND STUDY PARTICIPANT DETAILS
  - Cell lines and cell culture
  - Animal experiments
  - Human samples
- METHOD DETAILS
  - Cell viability and proliferation assays
  - Real-time PCR
  - Western blotting
  - Co-immunoprecipitation
  - Immunofluorescence staining
  - Flow cytometry
  - Immunohistochemistry (IHC)
  - siRNA and plasmid transfection
  - *In vitro* T cell-mediated tumor killing
  - Cellular thermal shift assay (CETSA)
  - Molecular docking
  - Microscale thermophoresis (MST)
  - Transcriptomics
  - Proteomics
  - Bioinformatics analysis
- QUANTIFICATION AND STATISTICAL ANALYSIS

SUPPLEMENTAL INFORMATION

Supplementary data related to this article can be found online at https://doi.org/10.1016/j.xcrm.2026.102920.

Received: August 16, 2025
Revised: January 20, 2026
Accepted: June 19, 2026
Published: July 13, 2026

REFERENCES

1. Singh, M., Morris, V.K., Bandey, I.N., Hong, D.S., and Kopetz, S. (2024). Advancements in combining targeted therapy and immunotherapy for colorectal cancer. Trends Cancer *10*, 598–609. https://doi.org/10.1016/j.trecan.2024.05.001.

(D) Representative immunofluorescence (IF) staining of pretreatment tumor biopsy specimens from a responder and a non-responder, showing the expression and spatial localization of PD-L1 (green) and β-TrCP (red). Nuclei were counterstained with DAPI (blue). Scale bar, 50 μm.
(E) Quantification of the mean fluorescence intensity (MFI) of PD-L1 and β-TrCP from IF analysis shown in (D). Data are presented for responders ($n = 16$) and non-responders ($n = 16$).
(F and G) Kaplan-Meier survival analysis showing that patients with high PD-L1 and low β-TrCP expression had significantly longer PFS ($p = 0.036$) (F) and OS ($p = 0.022$) (G).
Data in (E) are presented as mean ± SD. Two-tailed Student's t test was used for statistical quantification (A and E).
Survival differences in (F) and (G) were assessed using the log rank test. See also Figure S12.

2. Sharma, P., Goswami, S., Raychaudhuri, D., Siddiqui, B.A., Singh, P., Nagarajan, A., Liu, J., Subudhi, S.K., Poon, C., Gant, K.L., et al. (2023). Immune checkpoint therapy-current perspectives and future directions. Cell *186*, 1652–1669. https://doi.org/10.1016/j.cell.2023.03.006.

3. Sharma, P., Siddiqui, B.A., Anandhan, S., Yadav, S.S., Subudhi, S.K., Gao, J., Goswami, S., and Allison, J.P. (2021). The Next Decade of Immune Checkpoint Therapy. Cancer Discov. *11*, 838–857. https://doi.org/10.1158/2159-8290.CD-20-1680.

4. Cha, J.H., Chan, L.C., Li, C.W., Hsu, J.L., and Hung, M.C. (2019). Mechanisms Controlling PD-L1 Expression in Cancer. Mol. Cell *76*, 359–370. https://doi.org/10.1016/j.molcel.2019.09.030.

5. Zemek, R.M., Anagnostou, V., Pires da Silva, I., Long, G.V., and Lesterhuis, W.J. (2024). Exploiting temporal aspects of cancer immunotherapy. Nat. Rev. Cancer *24*, 480–497. https://doi.org/10.1038/s41568-024-00699-2.

6. Morad, G., Helmink, B.A., Sharma, P., and Wargo, J.A. (2022). Hallmarks of response, resistance, and toxicity to immune checkpoint blockade. Cell *185*, 576. https://doi.org/10.1016/j.cell.2022.01.008.

7. Emens, L.A., Romero, P.J., Anderson, A.C., Bruno, T.C., Capitini, C.M., Collyar, D., Gulley, J.L., Hwu, P., Posey, A.D., Jr., Silk, A.W., and Wargo, J.A. (2024). Challenges and opportunities in cancer immunotherapy: a Society for Immunotherapy of Cancer (SITC) strategic vision. J. Immunother. Cancer *12*, e009063. https://doi.org/10.1136/jitc-2024-009063.

8. Ramos-Casals, M., and Sisó-Almirall, A. (2024). Immune-Related Adverse Events of Immune Checkpoint Inhibitors. Ann. Intern. Med. *177*, ITC17–ITC32. https://doi.org/10.7326/AITC202402200.

9. Lin, X., Xie, M., Yao, J., Ma, X., Qin, L., Zhang, X.M., Song, J., Bao, X., Zhang, X., Zhang, Y., et al. (2024). Immune-related adverse events in non-small cell lung cancer: Occurrence, mechanisms and therapeutic strategies. Clin. Transl. Med. *14*, e1613. https://doi.org/10.1002/ctm2.1613.

10. Yi, M., Zheng, X., Niu, M., Zhu, S., Ge, H., and Wu, K. (2022). Combination strategies with PD-1/PD-L1 blockade: current advances and future directions. Mol. Cancer *21*, 28. https://doi.org/10.1186/s12943-021-01489-2.

11. Dai, X., Gao, Y., and Wei, W. (2022). Post-translational regulations of PD-L1 and PD-1: Mechanisms and opportunities for combined immunotherapy. Semin. Cancer Biol. *85*, 246–252. https://doi.org/10.1016/j.semcancer.2021.04.002.

12. Garcia-Diaz, A., Shin, D.S., Moreno, B.H., Saco, J., Escuin-Ordinas, H., Rodriguez, G.A., Zaretsky, J.M., Sun, L., Hugo, W., Wang, X., et al. (2019). Interferon Receptor Signaling Pathways Regulating PD-L1 and PD-L2 Expression. Cell Rep. *29*, 3766. https://doi.org/10.1016/j.celrep.2019.11.113.

13. Yan, Y., Zheng, L., Du, Q., Yan, B., and Geller, D.A. (2020). Interferon regulatory factor 1 (IRF-1) and IRF-2 regulate PD-L1 expression in hepatocellular carcinoma (HCC) cells. Cancer Immunol. Immunother. *69*, 1891–1903. https://doi.org/10.1007/s00262-020-02586-9.

14. Li, Z., Yu, X., Yuan, Z., Li, L., and Yin, P. (2024). New horizons in the mechanisms and therapeutic strategies for PD-L1 protein degradation in cancer. Biochim. Biophys. Acta Rev. Canc *1879*, 189152. https://doi.org/10.1016/j.bbcan.2024.189152.

15. Wang, Q., Wang, J., Yu, D., Zhang, Q., Hu, H., Xu, M., Zhang, H., Tian, S., Zheng, G., Lu, D., et al. (2024). Benzosceptrin C induces lysosomal degradation of PD-L1 and promotes antitumor immunity by targeting DHHC3. Cell Rep. Med. *5*, 101357. https://doi.org/10.1016/j.xcrm.2023.101357.

16. Su, W., Tan, M., Wang, Z., Zhang, J., Huang, W., Song, H., Wang, X., Ran, H., Gao, Y., Nie, G., and Wang, H. (2023). Targeted Degradation of PD-L1 and Activation of the STING Pathway by Carbon-Dot-Based PROTACs for Cancer Immunotherapy. Angew. Chem., Int. Ed. Engl. *62*, e202218128. https://doi.org/10.1002/anie.202218128.

17. Moon, Y., Cho, H., Kim, J., Song, S., Yeon Park, J., Young Min, J., Hee Han, E., Kim, Y., Seong, J.K., Kyu Shim, M., and Kim, K. (2025). Self-Assembled Peptide-Derived Proteolysis-Targeting Chimera (PROTAC) Nanoparticles for Tumor-Targeted and Durable PD-L1 Degradation in Cancer Immunotherapy. Angew. Chem., Int. Ed. Engl. *64*, e202414146. https://doi.org/10.1002/anie.202414146.

18. Yang, X., Wang, W., and Ji, T. (2024). Metabolic remodeling by the PD-L1 inhibitor BMS-202 significantly inhibits cell malignancy in human glioblastoma. Cell Death Dis. *15*, 186. https://doi.org/10.1038/s41419-024-06553-5.

19. Rui, M., Zhang, W., Mi, K., Ni, H., Ji, W., Yu, X., Qin, J., and Feng, C. (2023). Design and evaluation of alpha-helix-based peptide inhibitors for blocking PD-1/PD-L1 interaction. Int. J. Biol. Macromol. *253*, 126811. https://doi.org/10.1016/j.ijbiomac.2023.126811.

20. Yamaguchi, H., Hsu, J.M., Yang, W.H., and Hung, M.C. (2022). Mechanisms regulating PD-L1 expression in cancers and associated opportunities for novel small-molecule therapeutics. Nat. Rev. Clin. Oncol. *19*, 287–305. https://doi.org/10.1038/s41571-022-00601-9.

21. Adams, J.L., Smothers, J., Srinivasan, R., and Hoos, A. (2015). Big opportunities for small molecules in immuno-oncology. Nat. Rev. Drug Discov. *14*, 603–622. https://doi.org/10.1038/nrd4596.

22. Supuran, C.T. (2025). Multi- and polypharmacology of carbonic anhydrase inhibitors. Pharmacol. Rev. *77*, 100004. https://doi.org/10.1124/pharmrev.124.001125.

23. De Simone, G., Langella, E., Esposito, D., Supuran, C.T., Monti, S.M., Winum, J.Y., and Alterio, V. (2017). Insights into the binding mode of sulphamates and sulphamides to hCA II: crystallographic studies and binding free energy calculations. J. Enzym. Inhib. Med. Chem. *32*, 1002–1011. https://doi.org/10.1080/14756366.2017.1349764.

24. Youse, M.S., Abutaleb, N.S., Nocentini, A., S Abdelsattar, A., Ali, F., Supuran, C.T., Seleem, M.N., and Flaherty, D.P. (2024). Optimization of Ethoxzolamide Analogs with Improved Pharmacokinetic Properties for In Vivo Efficacy against Neisseria gonorrhoeae. J. Med. Chem. *67*, 15537–15556. https://doi.org/10.1021/acs.jmedchem.4c01187.

25. Petreni, A., Iacobescu, A., Simionescu, N., Petrovici, A.R., Angeli, A., Fifere, A., Pinteala, M., and Supuran, C.T. (2022). Carbonic Anhydrase Inhibitors bearing organotelluride moieties as novel agents for antitumor therapy. Eur. J. Med. Chem. *244*, 114811. https://doi.org/10.1016/j.ejmech.2022.114811.

26. Zhang, H., Zhang, Y., Feng, Z., Shuai, M., Ma, X., Wang, S., Yu, S., Deng, R., Luo, D., Shi, J., et al. (2024). Discovery of Novel Proteolysis-Targeting Chimera Molecules as Degraders of Programmed Cell Death-Ligand 1 for Breast Cancer Therapy. J. Med. Chem. *67*, 10589–10600. https://doi.org/10.1021/acs.jmedchem.3c02259.

27. Yi, M., Wu, Y., Niu, M., Zhu, S., Zhang, J., Yan, Y., Zhou, P., Dai, Z., and Wu, K. (2022). Anti-TGF-beta/PD-L1 bispecific antibody promotes T cell infiltration and exhibits enhanced antitumor activity in triple-negative breast cancer. J. Immunother. Cancer *10*, e005543. https://doi.org/10.1136/jitc-2022-005543.

28. van Gulijk, M., van Krimpen, A., Schetters, S., Eterman, M., van Elsas, M., Mankor, J., Klaase, L., de Bruijn, M., van Nimwegen, M., van Tienhoven, T., et al. (2023). PD-L1 checkpoint blockade promotes regulatory T cell activity that underlies therapy resistance. Sci. Immunol. *8*, eabn6173. https://doi.org/10.1126/sciimmunol.abn6173.

29. Jeong, H., Koh, J., Kim, S., Yim, J., Song, S.G., Kim, H., Li, Y., Lee, S.H., Chung, Y.K., Kim, H., et al. (2025). Cell-intrinsic PD-L1 signaling drives immunosuppression by myeloid-derived suppressor cells through IL-6/Jak/Stat3 in PD-L1-high lung cancer. J. Immunother. Cancer *13*, e010612. https://doi.org/10.1136/jitc-2024-010612.

30. Shah, S.C., and Itzkowitz, S.H. (2022). Colorectal Cancer in Inflammatory Bowel Disease: Mechanisms and Management. Gastroenterology *162*, 715–730.e3. https://doi.org/10.1053/j.gastro.2021.10.035.

31. Hu, H., Wang, Q., Yu, D., Tao, X., Guo, M., Tian, S., Zhang, Q., Xu, M., Geng, X., Zhang, H., et al. (2025). Berberine Derivative B68 Promotes Tumor Immune Clearance by Dual-Targeting BMI1 for Senescence Induction and CSN5 for PD-L1 Degradation. Adv. Sci. *12*, e2413122. https://doi.org/10.1002/advs.202413122.

32. Thanki, K.K., Johnson, P., Higgins, E.J., Maskey, M., Phillips, C., Dash, S., Almenas, F.A., Govar, A.A., Tian, B., Villéger, R., et al. (2022). Deletion of cystathionine-gamma-lyase in bone marrow-derived cells promotes colitis-associated carcinogenesis. Redox Biol. *55*, 102417. https://doi.org/10.1016/j.redox.2022.102417.

33. Gong, Y., Liu, Z., Yuan, Y., Yang, Z., Zhang, J., Lu, Q., Wang, W., Fang, C., Lin, H., and Liu, S. (2022). PUMILIO proteins promote colorectal cancer growth via suppressing p21. Nat. Commun. *13*, 1627. https://doi.org/10.1038/s41467-022-29309-1.

34. Guo, D., Zhang, M., Wei, T., Zhang, X., Shi, X., Tang, H., Ding, M., Li, J., Zhang, S., and Guo, W. (2024). NFKBIZ regulates NFkappaB signaling pathway to mediate tumorigenesis and metastasis of hepatocellular carcinoma by direct interaction with TRIM16. Cell. Mol. Life Sci. *81*, 167. https://doi.org/10.1007/s00018-024-05182-7.

35. Yu, X., Li, W., Liu, H., Wang, X., Coarfa, C., Cheng, C., Yu, X., Zeng, Z., Cao, Y., Young, K.H., and Li, Y. (2023). PD-L1 translocation to the plasma membrane enables tumor immune evasion through MIB2 ubiquitination. J. Clin. Investig. *133*, e160456. https://doi.org/10.1172/JCI160456.

36. Rape, M. (2018). Ubiquitylation at the crossroads of development and disease. Nat. Rev. Mol. Cell Biol. *19*, 59–70. https://doi.org/10.1038/nrm.2017.83.

37. Wang, P., Dai, X., Jiang, W., Li, Y., and Wei, W. (2020). RBR E3 ubiquitin ligases in tumorigenesis. Semin. Cancer Biol. *67*, 131–144. https://doi.org/10.1016/j.semcancer.2020.05.002.

38. Li, C.W., Lim, S.O., Xia, W., Lee, H.H., Chan, L.C., Kuo, C.W., Khoo, K.H., Chang, S.S., Cha, J.H., Kim, T., et al. (2016). Glycosylation and stabilization of programmed death ligand-1 suppresses T-cell activity. Nat. Commun. *7*, 12632. https://doi.org/10.1038/ncomms12632.

39. Zhang, J., Bu, X., Wang, H., Zhu, Y., Geng, Y., Nihira, N.T., Tan, Y., Ci, Y., Wu, F., Dai, X., et al. (2018). Cyclin D-CDK4 kinase destabilizes PD-L1 via cullin 3-SPOP to control cancer immune surveillance. Nature *553*, 91–95. https://doi.org/10.1038/nature25015.

40. Cha, J.H., Yang, W.H., Xia, W., Wei, Y., Chan, L.C., Lim, S.O., Li, C.W., Kim, T., Chang, S.S., Lee, H.H., et al. (2018). Metformin Promotes Antitumor Immunity via Endoplasmic-Reticulum-Associated Degradation of PD-L1. Mol. Cell *71*, 606–620.e7. https://doi.org/10.1016/j.molcel.2018.07.030.

41. Burr, M.L., Sparbier, C.E., Chan, Y.C., Williamson, J.C., Woods, K., Beavis, P.A., Lam, E.Y.N., Henderson, M.A., Bell, C.C., Stolzenburg, S., et al. (2017). CMTM6 maintains the expression of PD-L1 and regulates anti-tumour immunity. Nature *549*, 101–105. https://doi.org/10.1038/nature23643.

42. Qian, G., Guo, J., Vallega, K.A., Hu, C., Chen, Z., Deng, Y., Wang, Q., Fan, S., Ramalingam, S.S., Owonikoko, T.K., et al. (2021). Membrane-Associated RING-CH 8 Functions as a Novel PD-L1 E3 Ligase to Mediate PD-L1 Degradation Induced by EGFR Inhibitors. Mol. Cancer Res. *19*, 1622–1634. https://doi.org/10.1158/1541-7786.MCR-21-0147.

43. Wu, Y., Zhang, C., Liu, X., He, Z., Shan, B., Zeng, Q., Zhao, Q., Zhu, H., Liao, H., Cen, X., et al. (2021). ARIH1 signaling promotes anti-tumor immunity by targeting PD-L1 for proteasomal degradation. Nat. Commun. *12*, 2346. https://doi.org/10.1038/s41467-021-22467-8.

44. Xia, J., Xu, M., Hu, H., Zhang, Q., Yu, D., Cai, M., Geng, X., Zhang, H., Zhang, Y., Guo, M., et al. (2024). 5,7,4'-Trimethoxyflavone triggers cancer cell PD-L1 ubiquitin-proteasome degradation and facilitates antitumor immunity by targeting HRD1. MedComm *5*, e611. https://doi.org/10.1002/mco2.611.

45. Tian, S., Xu, M., Geng, X., Fang, J., Xu, H., Xue, X., Hu, H., Zhang, Q., Yu, D., Guo, M., et al. (2025). Network Medicine-Based Strategy Identifies Maprotiline as a Repurposable Drug by Inhibiting PD-L1 Expression via Targeting SPOP in Cancer. Adv. Sci. *12*, e2410285. https://doi.org/10.1002/advs.202410285.

46. Martinez Molina, D., Jafari, R., Ignatushchenko, M., Seki, T., Larsson, E.A., Dan, C., Sreekumar, L., Cao, Y., and Nordlund, P. (2013). Monitoring drug target engagement in cells and tissues using the cellular thermal shift assay. Science *341*, 84–87. https://doi.org/10.1126/science.1233606.

47. Yau, R., and Rape, M. (2016). The increasing complexity of the ubiquitin code. Nat. Cell Biol. *18*, 579–586. https://doi.org/10.1038/ncb3358.

48. Sun, Y., Jiang, L., Zhang, Z., Zhu, R., Liang, J., Liu, Z., He, Y., Huang, Z., Ling, C., Zhou, X., and Mao, X. (2025). RNF6 Inhibits Lung Adenocarcinoma Cell Proliferation by Promoting Cyclin D2 Degradation. Mol. Cancer Res. *23*, 426–437. https://doi.org/10.1158/1541-7786.MCR-24-0703.

49. Liu, X., Cui, L., Tao, Y., Xia, S., Hou, J., Cao, X., and Xu, S. (2024). The deubiquitinase BAP1 and E3 ligase UBE3C sequentially target IRF3 to activate and resolve the antiviral innate immune response. Cell Rep. *43*, 114608. https://doi.org/10.1016/j.celrep.2024.114608.

50. Hao, J., Li, J., Zhang, Z., Yang, Y., Zhou, Q., Wu, T., Chen, T., Wu, Z., Zhang, P., Cui, J., and Li, Y.P. (2023). NLRC5 restricts dengue virus infection by promoting the autophagic degradation of viral NS3 through E3 ligase CUL2 (cullin 2). Autophagy *19*, 1332–1347. https://doi.org/10.1080/15548627.2022.2126614.

51. Rowshanravan, B., Halliday, N., and Sansom, D.M. (2018). CTLA-4: a moving target in immunotherapy. Blood *131*, 58–67. https://doi.org/10.1182/blood-2017-06-741033.

52. Edner, N.M., Carlesso, G., Rush, J.S., and Walker, L.S.K. (2020). Targeting co-stimulatory molecules in autoimmune disease. Nat. Rev. Drug Discov. *19*, 860–883. https://doi.org/10.1038/s41573-020-0081-9.

53. Lin, X., Ma, X., Zhao, S., Yao, J., Han, L., Jing, Y., and Xue, X. (2024). Cardiovascular toxicity in antitumor therapy: biological and therapeutic insights. Trends Cancer *10*, 920–934. https://doi.org/10.1016/j.trecan.2024.07.004.

54. Elsawi, A.E., Elbadawi, M.M., Nocentini, A., Almahli, H., Giovannuzzi, S., Shaldam, M., Salem, R., Ibrahim, T.M., Abdel-Aziz, H.A., Supuran, C.T., and Eldehna, W.M. (2023). 1,5-Diaryl-1,2,4-triazole Ureas as New SLC-0111 Analogues Endowed with Dual Carbonic Anhydrase and VEGFR-2 Inhibitory Activities. J. Med. Chem. *66*, 10558–10578. https://doi.org/10.1021/acs.jmedchem.3c00721.

55. Zheng, C., Song, Q., Zhao, H., Kong, Y., Sun, L., Liu, X., Feng, Q., and Wang, L. (2021). A nanoplatform to boost multi-phases of cancer-immunity-cycle for enhancing immunotherapy. J. Contr. Release *339*, 403–415. https://doi.org/10.1016/j.jconrel.2021.10.011.

56. Chen, X., Ding, J.C., Hu, G.S., Shu, X.Y., Liu, Y., Du, J., Wen, Z.J., Liu, J.Y., Huang, H.H., Tang, G.H., and Liu, W. (2023). Estrogen-Induced LncRNA, LINC02568, Promotes Estrogen Receptor-Positive Breast Cancer Development and Drug Resistance Through Both In Trans and In Cis Mechanisms. Adv. Sci. *10*, e2206663. https://doi.org/10.1002/advs.202206663.

57. Bonardi, A., Nocentini, A., Giovannuzzi, S., Paoletti, N., Ammara, A., Bua, S., Abutaleb, N.S., Abdelsattar, A.S., Capasso, C., Gratteri, P., et al. (2024). Development of Penicillin-Based Carbonic Anhydrase Inhibitors Targeting Multidrug-Resistant Neisseria gonorrhoeae. J. Med. Chem. *67*, 9613–9627. https://doi.org/10.1021/acs.jmedchem.4c00740.

58. Ning, W.R., Jiang, D., Liu, X.C., Huang, Y.F., Peng, Z.P., Jiang, Z.Z., Kang, T., Zhuang, S.M., Wu, Y., and Zheng, L. (2022). Carbonic anhydrase XII mediates the survival and prometastatic functions of macrophages in human hepatocellular carcinoma. J. Clin. Investig. *132*, e153110. https://doi.org/10.1172/JCI153110.

59. Deng, C., Ma, J., Liu, Y., Tong, X., Wang, L., Dong, J., Shi, P., Wang, M., Zheng, W., and Ma, X. (2025). Targeting intracellular cancer proteins with tumor-microenvironment-responsive bispecific nanobody-PROTACs for enhanced therapeutic efficacy. MedComm *6*, e70068. https://doi.org/10.1002/mco2.70068.

60. Deng, L., Qian, G., Zhang, S., Zheng, H., Fan, S., Lesinski, G.B., Owonikoko, T.K., Ramalingam, S.S., and Sun, S.Y. (2019). Inhibition of mTOR complex 1/p70 S6 kinase signaling elevates PD-L1 levels in human cancer cells through enhancing protein stabilization accompanied with enhanced beta-TrCP degradation. Oncogene *38*, 6270–6282. https://doi.org/10.1038/s41388-019-0877-4.

61. Wang, Z., Shaabani, S., Gao, X., Ng, Y.L.D., Sapozhnikova, V., Mertins, P., Krönke, J., and Dömling, A. (2023). Direct-to-biology, automated, nano-scale synthesis, and phenotypic screening-enabled E3 ligase modulator discovery. Nat. Commun. *14*, 8437. https://doi.org/10.1038/s41467-023-43614-3.

62. Lee, P., Chandel, N.S., and Simon, M.C. (2020). Cellular adaptation to hypoxia through hypoxia inducible factors and beyond. Nat. Rev. Mol. Cell Biol. *21*, 268–283. https://doi.org/10.1038/s41580-020-0227-y.

63. Liu, H., Tang, Y., Singh, A., Vong, J., Cordero, J., Mathes, A., Gao, R., Jia, Y., Garvalov, B.K., Acker, T., et al. (2025). RNF20 links the DNA damage response and metabolic rewiring in lung cancer through HIF1alpha. Nat. Commun. *16*, 4929. https://doi.org/10.1038/s41467-025-60223-4.

64. Vassilev, L.T., Vu, B.T., Graves, B., Carvajal, D., Podlaski, F., Filipovic, Z., Kong, N., Kammlott, U., Lukacs, C., Klein, C., et al. (2004). In vivo activation of the p53 pathway by small-molecule antagonists of MDM2. Science *303*, 844–848. https://doi.org/10.1126/science.1092472.

65. Hafner, A., Bulyk, M.L., Jambhekar, A., and Lahav, G. (2019). The multiple mechanisms that regulate p53 activity and cell fate. Nat. Rev. Mol. Cell Biol. *20*, 199–210. https://doi.org/10.1038/s41580-019-0110-x.

66. Fan, J., Bellon, M., Ju, M., Zhao, L., Wei, M., Fu, L., and Nicot, C. (2022). Clinical significance of FBXW7 loss of function in human cancers. Mol. Cancer *21*, 87. https://doi.org/10.1186/s12943-022-01548-2.

67. Simonetta, K.R., Taygerly, J., Boyle, K., Basham, S.E., Padovani, C., Lou, Y., Cummins, T.J., Yung, S.L., von Soly, S.K., Kayser, F., et al. (2019). Prospective discovery of small molecule enhancers of an E3 ligase-substrate interaction. Nat. Commun. *10*, 1402. https://doi.org/10.1038/s41467-019-09358-9.

68. Liu, J., Bai, W., Zhou, T., Xie, Y., Yang, B., Sun, J., Wang, Y., Li, X., Hou, X., Liu, Z., et al. (2023). SDCBP promotes pancreatic cancer progression by preventing YAP1 from beta-TrCP-mediated proteasomal degradation. Gut *72*, 1722–1737. https://doi.org/10.1136/gutjnl-2022-327492.

69. Huang, W., Wu, D., Cai, C., Yao, H., Tian, Z., Yang, Y., Pang, M., Rong, L., and Liu, B. (2024). Inhibition of MST1 ameliorates neuronal apoptosis via GSK3beta/beta-TrCP/NRF2 pathway in spinal cord injury accompanied by diabetes. Redox Biol. *71*, 103104. https://doi.org/10.1016/j.redox.2024.103104.

70. Mok, T.S.K., Wu, Y.L., Kudaba, I., Kowalski, D.M., Cho, B.C., Turna, H.Z., Castro, G., Jr., Srimuninnimit, V., Laktionov, K.K., Bondarenko, I., et al. (2019). Pembrolizumab versus chemotherapy for previously untreated, PD-L1-expressing, locally advanced or metastatic non-small-cell lung cancer (KEYNOTE-042): a randomised, open-label, controlled, phase 3 trial. Lancet *393*, 1819–1830. https://doi.org/10.1016/S0140-6736(18)32409-7.

71. Lin, X., Kang, K., Chen, P., Zeng, Z., Li, G., Xiong, W., Yi, M., and Xiang, B. (2024). Regulatory mechanisms of PD-1/PD-L1 in cancers. Mol. Cancer *23*, 108. https://doi.org/10.1186/s12943-024-02023-w.

72. Deutsch, J.S., Cimino-Mathews, A., Thompson, E., Provencio, M., Forde, P.M., Spicer, J., Girard, N., Wang, D., Anders, R.A., Gabrielson, E., et al. (2024). Association between pathologic response and survival after neoadjuvant therapy in lung cancer. Nat. Med. *30*, 218–228. https://doi.org/10.1038/s41591-023-02660-6.

73. Spicer, J.D., Garassino, M.C., Wakelee, H., Liberman, M., Kato, T., Tsuboi, M., Lee, S.H., Chen, K.N., Dooms, C., Majem, M., et al. (2024). Neoadjuvant pembrolizumab plus chemotherapy followed by adjuvant pembrolizumab compared with neoadjuvant chemotherapy alone in patients with early-stage non-small-cell lung cancer (KEYNOTE-671): a randomised, double-blind, placebo-controlled, phase 3 trial. Lancet *404*, 1240–1252. https://doi.org/10.1016/S0140-6736(24)01756-2.

74. Maren, T.H. (1967). Carbonic anhydrase: chemistry, physiology, and inhibition. Physiol. Rev. *47*, 595–781. https://doi.org/10.1152/physrev.1967.47.4.595.

75. Deng, J., Zhao, L., Yuan, X., Li, Y., Shi, J., Zhang, H., Zhao, Y., Han, L., Wang, H., Yan, Y., et al. (2022). Pre-Administration of Berberine Exerts Chemopreventive Effects in AOM/DSS-Induced Colitis-Associated Carcinogenesis Mice via Modulating Inflammation and Intestinal Microbiota. Nutrients *14*, 726. https://doi.org/10.3390/nu14040726.

STAR★METHODS

KEY RESOURCES TABLE

REAGENT or RESOURCE	SOURCE	IDENTIFIER
**Antibodies**		
Anti-PD-L1 (for Western blot)	Abcam	Cat# ab213524; RRID: AB_2857903
PD-L1 antibody (for Immunoprecipitation)	Proteintech	Cat# 17952-1-AP; RRID: AB_10597552
PE anti-human CD274 (B7-H1, PD-L1) (Flow Cytometry)	Biolegend	Cat# 329706; RRID: AB_940368
InVivoMab anti-mouse PD-L1 (B7-H1) (for *in vivo*)	Bioxcell	Cat# BE0101; RRID: AB_10949073
InVivoPlus anti-mouse CTLA-4 (CD152) (for *in vivo*)	Bioxcell	Cat# BP0032; RRID: AB_2894790
β-TrCP antibody (for Western blot)	Proteintech	Cat# 28393-1-AP; RRID: AB_2935467
β-TrCP Rabbit mAb (for Immunoprecipitation)	Cell Signaling Technology	Cat# 11984; RRID: AB_10545763
Recombinant Anti-Ubiquitin Antibody	Abcam	Cat# ab134953; RRID: AB_2801561
GAPDH antibody	Proteintech	Cat# 60004-1; RRID: AB_2107436
PE anti-mouse CD25	Biolegend	Cat# 101904; RRID: AB_312847
AF700 anti-Granzyme B	Biolegend	Cat# 372222; RRID: AB_2728389
APC-Cy7 anti-mouse CD3ε	Multi Sciences	Cat# 560176; RRID: AB_1645475
PerCP-Cy5.5 anti-mouse CD8α	Multi Sciences	Cat# 561109; RRID: AB_10563417
PE-Cy7 anti-mouse CD4	Multi Sciences	Cat# 563933; RRID: AB_2738492
APC anti-mouse Foxp3	Multi Sciences	Cat# A27056; RRID: AB_2536115
BV421 anti-mouse Ly-6G/Ly-6C	BD Biosciences	Cat# 756295; RRID: AB_3688533
mFluor 450 anti-CD11b (M1/70)	Multi Sciences	Cat# 48-0112-82; RRID: AB_1582236
FITC anti-mouse MHC II (I-A/I-E)	Multi Sciences	Cat# 65122-1; RRID: AB_2918416
PE anti-mouse CD11c (Clone HL3)	Multi Sciences	Cat# 561044; RRID: AB_2033996
PE-Cy7 anti-mouse F4/80	Multi Sciences	Cat# 569224; RRID: AB_3684883
APC anti-mouse CD80	Biolegend	Cat# 104714; RRID: AB_313135
AF700 anti-mouse CD206 (MMR)	Biolegend	Cat# 141734; RRID: AB_2629637
Brilliant Violet 605(TM) anti-mouse CD86	Biolegend	Cat# 105037; RRID: AB_11204429
BUV395 anti-mouse CD45R (B220) (RA3-6B2)	Invitrogen	Cat# 363-0452-82; RRID: AB_2925266
PE anti-mouse CD19	Multi Sciences	Cat# 561736; RRID: AB_10896141
FITC anti-mouse NK1.1	Multi Sciences	Cat# 561082; RRID: AB_10563221
FITC anti-mouse CD45 (I3/2.3)	Absin	Cat# LS-C45195; RRID: AB_1060104
**Biological samples**		
Human lung cancer samples	Beijing Shijitan Hospital; Beijing Chaoyang Hospital, Capital Medical University	N/A
**Chemicals, peptides, and recombinant proteins**		
Ethoxzolamide	MedChemExpress	Cat# HY-101950
Ethoxzolamide	Macklin	Cat# C125195
Fetal Bovine Serum (FBS)	Biological Industries	Cat# 04-001-1ACS
Methazolamide	MedChemExpress	Cat# HY-B0553
Acetazolamide	MedChemExpress	Cat# HY-B0782
PA8	MedChemExpress	Cat# HY-163757
MG-132	MedChemExpress	Cat# HY-13259
Chloroquine (CQ)	MedChemExpress	Cat# HY-17589A
Bafilomycin (BAF)	MedChemExpress	Cat# HY-100558
3-Methyladenine (3-MA)	MedChemExpress	Cat# HY-19312

(*Continued on next page*)

Continued		
REAGENT or RESOURCE	SOURCE	IDENTIFIER
Cycloheximide (CHX)	MedChemExpress	Cat# HY-12320
Azoxymethane (AOM)	Sigma-Aldrich	Cat# A5486
Dextran Sulfate Sodium Salt (DSS)	Meilunbio	Cat# MB1286
Phytohemagglutinin (PHA)	Sigma-Aldrich	Cat# L1668
Phorbol 12-myristate 13-acetate (PMA)	Sigma-Aldrich	Cat# P8139
Collagenase IV	Sigma-Aldrich	Cat# C5138
DNase I	Sigma-Aldrich	Cat# DN25
Critical commercial assays		
Cell Counting Kit-8 (CCK-8)	Meilunbio	Cat# MA0218
EdU-488 Assay Kit	Beyotime	Cat# C0071S
PrimeScript RT Reagent Kit	Takara	Cat# RR037A
BCA Protein Assay Kit	Beyotime	Cat# P0012S
Deposited data		
RNA-Seq data of EZA treated RKO cells	GEO	GSE318640
Proteins data of EZA treated RKO cells	ProteomeXchange (PRIDE)	PXD074302
Experimental models: Cell lines		
Human: H1975	ATCC	Cat# CRL-5908; RRID: CVCL_1511
Mouse: MC38	ATCC	Cat# CRL-2640; RRID: CVCL_J763
Human: 293T	ATCC	Cat# CRL-3216; RRID: CVCL_0063
Human: RKO	ATCC	Cat# CRL-2577; RRID: CVCL_0504
Mouse: Lewis Lung cancer	ATCC	Cat# CRL-1642; RRID: CVCL_4358
Human: Jurkat	Gift from Prof. Kongming Wu (Tongji Hospital)	N/A
Experimental models: Organisms/strains		
Mouse: C57BL/6J (female/male)	Shanghai Jihui Laboratory Animal Co., Ltd	N/A
Mouse: BALB/c nude (female)	Shanghai Jihui Laboratory Animal Co., Ltd	N/A
Oligonucleotides		
siRNA	GenePharma	Sequences in Table S2
RT-PCR Primers	This paper	Sequences in Table S2
Recombinant DNA		
pcDNA3.1-Ub	This paper	N/A
pcDNA3.1-β-TrCP	This paper	N/A
pcDNA3.1-PD-L1	This paper	N/A
Software and algorithms		
GraphPad Prism v10.3.0	GraphPad Software	https://www.graphpad.com
FlowJo v10.8.1	BD Biosciences	https://www.flowjo.com
ImageJ	NIH	https://imagej.nih.gov/ij
ImageLab	Bio-Rad	https://www.bio-rad.com
Molecular Operating Environment	Chemical Computing Group	https://www.chemcomp.com
MO. Affinity Analysis v2.3	NanoTemper	https://nanotempertech.com
Large-scale Protein Preparation System	National Facility for Protein Science	https://cstr.cn/31129.02.NFPS.LSPS

EXPERIMENTAL MODEL AND STUDY PARTICIPANT DETAILS

Cell lines and cell culture

H1975, MC38, and 293T cells were cultured in DMEM (Meilunbio, China). RKO cells were maintained in MEM (Meilunbio, China), while LLC and Jurkat cells were cultured in RPMI-1640 medium (Meilunbio, China). All media were supplemented with 10% fetal bovine serum (FBS; Biological Industries, USA), 100 U/mL penicillin, and 100 μg/mL streptomycin (Meron Bio, China). Cells were incubated at 37°C in a humidified atmosphere with 5% CO_2 (Thermo Fisher Scientific, USA). The Jurkat cell line was kindly provided by Prof. Kongming Wu's research group (Tongji Hospital, Tongji Medical College, Huazhong University of Science and Technology). All other cell lines, including human-derived H1975, 293T, RKO, and mouse-derived MC38 and LLC, were purchased from the

CellPress
OPEN ACCESS

American Type Culture Collection (ATCC). All human cell lines obtained from ATCC were authenticated by short tandem repeat (STR) profiling by the vendor prior to distribution. The engineered Jurkat-PD-1 cells overexpressing PD-1 and granzyme B (GZMB) were verified via qPCR assay before experiments. All cell lines were routinely tested and confirmed negative for mycoplasma contamination using a qPCR-based Mycoplasma Detection Kit (Meilunbio, China).

Animal experiments

All animal procedures were reviewed and approved by the Experimental Animal Ethics Committee of Shanghai University of Traditional Chinese Medicine (Approval number: PZSHUTCM2507100007). Female and male C57BL/6J mice and female BALB/c nude mice (6–8 weeks old, 18–20 g) were purchased from Shanghai Jihui Laboratory Animal Co., Ltd. All operations were conducted in a specific pathogen-free (SPF) facility maintained at a controlled temperature of 22 ± 2°C, 50–60% humidity, and a 12 h light/dark cycle with *ad libitum* access to standard chow and sterile water. For subcutaneous tumor models, LLC (3×10^7) or MC38 cells (1×10^6) were injected subcutaneously into wild-type mice. When tumors reached ~50 mm^3, mice were randomized into groups. EZA was administered daily (oral gavage in corn oil). Anti-PD-L1 (100 μg/Mouse) and anti-CTLA4 antibodies (100 μg/Mouse) were injected intraperitoneally every 5 days. Tumor volume and body weight were monitored every two days. For AOM/DSS-induced Colitis-Associated CRC Model, male wild-type C57BL/6J mice were administered a single intraperitoneal injection of 12.5 mg/kg azoxymethane (AOM; Sigma-Aldrich, USA) to initiate tumorigenesis.[33] One week later, mice received 2.5% dextran sulfate sodium (DSS; Meilunbio, China) in their drinking water for 7 consecutive days to induce colitis, followed by 14 days of regular water. This cycle was repeated three times.[75]

Human samples

A multicenter retrospective cohort study was conducted to evaluate patients with locally advanced NSCLC who received neoadjuvant immunochemotherapy (PD-1/PD-L1 inhibitors combined with chemotherapy) at Beijing Shijitan Hospital and Beijing Chaoyang Hospital, Capital Medical University. A total of 32 eligible patients were enrolled (total sample size $N = 32$). The collected data included demographics (including age, biological sex, and self-reported race/ethnicity, which was 100% Chinese Han), ECOG performance status, CT imaging, histological subtype, treatment response (according to RECIST 1.1), and survival outcomes. Detailed clinical characteristics of the cohort are summarized in Table S1. Subsequent analysis demonstrated that biological sex had no significant influence on the clinical parameters or treatment response. All surgical specimens were processed using standardized protocols and subjected to IF staining. This study was approved by the Medical Ethics Committee of Beijing Shijitan Hospital (Approval No. sjtkyll-lx-2022(35)). Written informed consent was obtained from all participants. The study strictly adhered to the principles of the Declaration of Helsinki and the ethical guidelines for biomedical research involving human subjects issued by the Chinese authorities. As this study was an observational retrospective cohort design evaluating pre-existing clinical outcomes, patients were not prospectively or randomly allocated to experimental arms by the investigators. Instead, allocation into experimental groups (responders, $n = 16$ vs. non-responders, $n = 16$) was defined retrospectively based on whether they achieved a major pathological response (MPR) or complete pathological response (pCR) at the surgical resection endpoint.

METHOD DETAILS

Cell viability and proliferation assays

For CCK-8 Assay, cells were seeded at 5×10^3 cells/well in 96-well plates. After treatment with EZA for 24 h, CCK-8 reagent was added and incubated for 2 h at 37°C. Absorbance was measured at 450 nm. For EdU Assay, cells were seeded at 2×10^5 cells/well in 12-well plates, treated with EZA for 24 h, and then processed using the EdU-488 Cell Proliferation Assay Kit according to the manufacturer's instructions. Fluorescence images were captured and analyzed using a Cytation 5 imaging system (BioTek).

Real-time PCR

Total RNA was extracted using TRIzol reagent. cDNA was synthesized using the PrimeScript RT Reagent Kit. RT-PCR was performed using the LightCycler 96 System (Roche). Primer sequences are listed in Table S2.

Western blotting

Cells were lysed with RIPA lysis buffer (Beyotime) supplemented with 1% PMSF (MCE). Protein concentrations were determined by BCA assay. Equal amounts of protein were separated by SDS-PAGE, transferred to PVDF membranes, blocked with 5% non-fat milk, and incubated with primary antibodies overnight at 4°C and HRP-conjugated secondary antibodies for 1 h at room temperature. Signals were visualized using a Bio-Rad imaging system.

Co-immunoprecipitation

Cells were lysed with prechilled IP lysis buffer with protease inhibitors (Beyotime, China). The lysates were incubated with specific antibodies overnight at 4°C with rotation, followed by incubation with protein A/G magnetic beads (Santa Cruz) for 3 h. The beads

were washed five times and eluted by boiling in 2× SDS loading buffer at 95°C for 10 min. The eluates were analyzed by Western blotting. IgG isotype controls were included, and all procedures were performed at 4°C to preserve protein integrity.

Immunofluorescence staining

Cells seeded on coverslips were treated with EZA for 24 h, fixed with 4% paraformaldehyde, blocked with 5% BSA, and incubated with primary antibodies overnight at 4°C and fluorophore-conjugated secondary antibodies for 1 h at room temperature. Nuclei were stained with DAPI. Images were acquired using a Cytation 5 Imaging System (BioTek).

Flow cytometry

Cell surface PD-L1 expression was quantified via flow cytometry. After EZA treatment, cells were washed with ice-cold PBS and incubated with PE-conjugated anti-human CD274 (1:100 dilution) for 30 min at 4°C in the dark. After being washed with PBS, the samples were analyzed via a Beckman Coulter Cytoflex flow cytometer (USA). Tumor tissues were digested with collagenase IV (1 mg/mL) and DNase I (0.1 mg/mL) at 37°C for 1 h to obtain single-cell suspensions. Cells were stained with surface antibody cocktails (CD3, CD4, CD8, CD25, CD11b, Gr-1, F4/80, CD80, CD86, CD206, CD11c, MHC II, NK1.1) at 4°C for 30 min. For intracellular Foxp3 staining, cells were fixed and permeabilized prior to staining. Data were acquired on a Beckman CytoFLEX and analyzed with FlowJo v10.8.1.

Immunohistochemistry (IHC)

Fresh tissues were fixed in 4% paraformaldehyde, paraffin-embedded, and sectioned. IHC was performed for CD8, cleaved caspase-3, Ki-67, Foxp3, TUNEL, CD86, CD206, F4/80, NK1.1, CD11c, PD-L1, and β-TrCP.

siRNA and plasmid transfection

RKO cells were transfected with gene-specific siRNAs (PD-L1, β-TrCP, HRD1, ARIH1) (GenePharma, China) or overexpression plasmids (pcDNA3.1-Ub, pcDNA3.1-β-TrCP, pcDNA3.1-PD-L1) via Lipofectamine 2000 (Invitrogen, USA). 293T cells were transfected with polyethyleneimine (PEI; Polysciences, USA). After 6–8 h, the media were replaced with complete medium, and the cells were cultured for 36–48 h prior to drug treatment. The transfection efficiency was validated by qPCR and Western blot (Table S2). Key functional mutants were generated using site-directed mutagenesis PCR, including: (1) substrate-binding domain mutants of β-TrCP (Leu347A, Leu387A, and Arg560A); (2) phosphorylation site mutants of PD-L1 (T180A/S184A). All mutant plasmids were verified via Sanger sequencing (Jinzhiwei, Shanghai).

In vitro T cell-mediated tumor killing

To assess the effect of EZA on tumor cell susceptibility to T cell-mediated killing, two complementary T cell models were employed. In the first model, given that wild-type Jurkat cells exhibit CD4-biased characteristics and lack endogenous GZMB expression—a key effector molecule required for cytotoxicity—we employed engineered Jurkat-PD-1 cells that were co-transduced to stably overexpress both human PD-1 and GZMB. This dual-overexpression system enables the cells to recapitulate key functional aspects of cytotoxic T cells *in vitro*. Adherent tumor cells were pretreated with EZA for 12 h and then co-cultured for 48 h with PD-1-overexpressing Jurkat T cells at an effector-to-target (E:T) ratio of 9:1 that had been pre-activated with 1 μg/mL PHA and 50 ng/mL PMA for 12 h. In the second model, primary human T cells were expanded from healthy-donor PBMCs by 7-day culture in CTSTM AIM V SFM medium supplemented with 1000 U/mL recombinant human IL-2 and a human CD3/CD28/CD2 T cell activator. Tumor cells (RKO, H1975) were seeded, treated with indicated concentrations of EZA for 12 h, and then co-cultured with the activated primary T cells for 48 h at an E:T ratio of 3:1. After co-culture, non-adherent cells and debris were removed by washing, and the remaining viable adherent tumor cells were stained with crystal violet for 15 min. Images of stained cells were acquired and analyzed using a Cytation 5 Imaging System (BioTek).

Cellular thermal shift assay (CETSA)

RKO cells were harvested and lysed via IP lysis buffer supplemented with 1% protease inhibitor cocktail (Beyotime, China). The supernatant was incubated with 100 μM EZA or DMSO at room temperature for 10 min. The mixtures were aliquoted and subjected to heat treatment at different temperatures (37°C–52°C) for 3 min. After centrifugation (12,000×g, 10 min, 4°C), the supernatants were analyzed by Western blotting to assess the thermal stability of the target proteins.

Molecular docking

The 3D structure of EZA was retrieved from the PubChem database (https://pubchem.ncbi.nlm.nih.gov/), and the amino acid sequence of β-TrCP was obtained from the UniProt database (https://www.uniprot.org/). Molecular docking was performed via Molecular Operating Environment (MOE). The protein structure was prepared by protonation and energy minimization. Potential binding pockets on β-TrCP were predicted using the SiteFinder module. EZA was prepared by conformational search and energy optimization. Docking of EZA into the predicted sites was performed, and resulting poses were ranked based on binding free energy (ΔG, S-score) and analysis of specific molecular interactions (e.g., hydrogen bonds, hydrophobic contacts). Residues Leu347, Leu387, and Arg560 were selected for subsequent experimental mutagenesis based on their consistent appearance in high-ranking poses,

their substantial contribution to the calculated binding energy, and their predicted involvement in key interactions with EZA within the binding pocket.

Microscale thermophoresis (MST)

Based on docking results, WT GFP-β-TrCP and the Leu347A/Leu387A/Arg560A mutants were transfected into 293T cells for 48 h. Cell lysates were mixed with EZA at 16 concentrations, and the binding affinity was measured using a Monolith NT.115 MST instrument (NanoTemper, Germany). Dissociation constants (Kd) were calculated by nonlinear fitting with MO. Affinity Analysis software.

Transcriptomics

Total RNA was extracted from EZA-treated and control RKO cells ($n = 3$ per group) via the TRIzol method. RNA quality was assessed via agarose gel electrophoresis, NanoDrop spectrophotometry (OD260/280 $\geq$ 1.8, OD260/230 $\geq$ 2.0), and an Agilent 2100 Bioanalyzer (RIN >7.0). Messenger RNA was enriched using oligo(dT) magnetic beads, and sequencing libraries were constructed using the NEBNext Ultra RNA Library Prep Kit, generating fragments of 250–300 bp. Paired-end sequencing was performed on the Illumina platform. Bioinformatic analysis included quality control of raw reads using FastQC, alignment to the human reference genome (hg38) with HISAT2, gene expression quantification using FeatureCounts, and differential expression analysis using DESeq2. Genes with a |log2(fold change)| $\geq$ 1 and a p value <0.05 were considered differentially expressed. KEGG pathway enrichment analysis was subsequently conducted to identify biological processes associated with these genes.

Proteomics

RKO cells were treated with 40 μM EZA or DMSO for 24 h before protein extraction. Protein samples were lysed in 8M urea containing protease inhibitors, followed by centrifugation to collect the supernatant. Protein concentration was measured, and samples were reduced with DTT, diluted in ABC buffer, and digested with trypsin. Peptides were desalted using C18 columns, concentrated, and freeze-dried for storage. Liquid chromatography-tandem mass spectrometry (LC-MS/MS) was performed using a Q Exactive HF-X mass spectrometer coupled with an EASY-nLC 1200 system, which employs a 60-min gradient elution. Data acquisition was carried out in data-dependent Top-40 mode with high resolution. Protein identification and quantification were conducted via Proteome Discoverer 2.4, referencing the UniProt human protein database, with the false discovery rate (FDR) controlled below 1%. The functional annotation and analysis of the differentially expressed proteins were performed via the InterProScan-5, COG, and KEGG databases.

Bioinformatics analysis

This study utilized the Kaplan-Meier Plotter database to obtain immunotherapy cohort data for COAD and LUAD patients. These datasets were used to evaluate the prognostic and predictive value of β-TrCP (BTRC) and PD-L1 (CD274) expression in relation to patient survival and response to immunotherapy. For the TCGA-COAD cohort ($N = 282$), the ESTIMATE algorithm (v 1.0.13) was applied to calculate immune-related scores of the TME, including the stromal score, to assess the associations between β-TrCP and PD-L1 expression and immune infiltration. Correlation analyses were conducted via the corr.test function. Further correlation analysis was performed via the TIMER database (Tumor IMmune Estimation Resource) to explore the relationships between β-TrCP expression and the infiltration levels of various immune cell subsets, including B cells, $CD8^+$ T cells, macrophages, and dendritic cells. Pearson correlation coefficients were calculated and adjusted for tumor purity to improve the accuracy and robustness of the results. In addition, a pancancer dataset containing normalized expression profiles (TCGA, TARGET, GTEx; PANCAN; $N = 19,131$ samples, G = 60,499 genes) from the UCSC Xena platform was used to investigate the correlation between β-TrCP expression and a curated panel of 150 immune-related marker genes. These genes represent five major immune function categories: chemokines ($n = 41$), chemokine receptors ($n = 18$), MHC genes ($n = 21$), immunoinhibitory factors ($n = 24$), and immunostimulatory factors ($n = 46$). Data preprocessing steps included filtering for primary tumor samples, excluding genes with zero expression values and normal tissue samples, and applying $\log_2(x + 0.001)$ transformation to all gene expression values. Correlation analyses were performed using Pearson correlation based on expression matrices annotated by standardized gene symbols.

QUANTIFICATION AND STATISTICAL ANALYSIS

Experimental data are presented as mean ± SD. Statistical analyses were performed using GraphPad Prism v10.3.0. Two-tailed Student's t test was used for comparisons between two groups. One-way ANOVA or two-way ANOVA was used for comparisons among multiple groups. Statistical significance was defined as $*p < 0.05$, $**p < 0.01$, $***p < 0.001$, $****p < 0.0001$.

Cell Reports Medicine, Volume *7*

Supplemental information

Repurposed ethoxzolamide reprograms antitumor immunity through β-TrCP-dependent PD-L1 ubiquitination

Xuwen Lin, Qun Wang, Mengting Xu, Dianping Yu, Hongmei Hu, Qing Zhang, Jiannan Yao, Mei Xie, Hanchi Xu, Xuefeng Zang, Jia Li, Yu Chen, Linyang Li, Xiaoyu Tao, Xinru Li, Simeng Li, Shize Xie, Yating Tian, Weidong Zhang, Sanhong Liu, and Xinying Xue

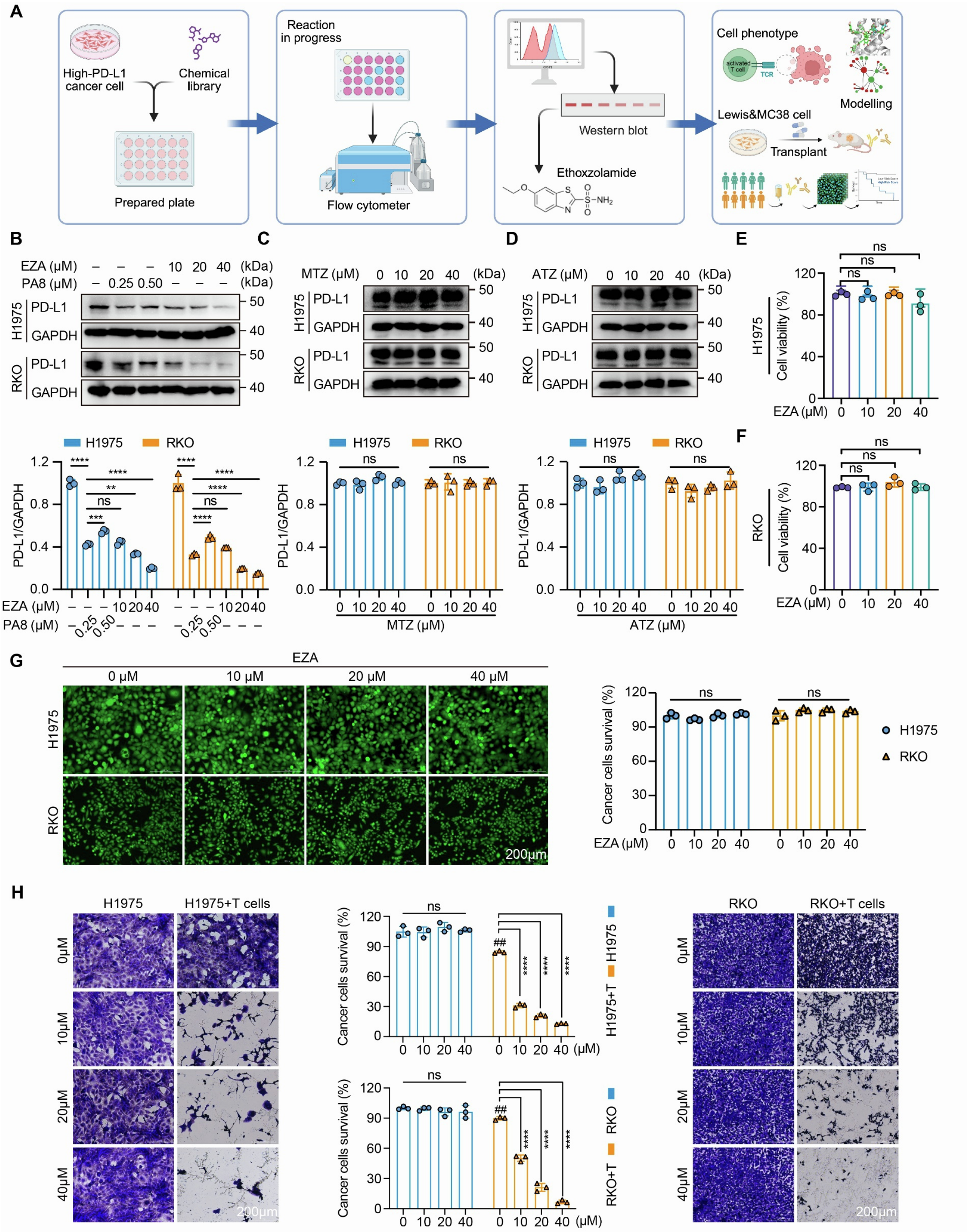

Figure S1. EZA reduces PD-L1 expression without significant cytotoxicity in cancer cells, related to Figure 1.

(A) Workflow for screening a small molecule compound library that reduces PD-L1 expression.

(B) Western blot analysis (top) and quantification (bottom) of PD-L1 protein levels in H1975 and RKO cells treated with the indicated concentrations of EZA for 24 hours. PA8, a known PROTAC PD-L1 degrader (0.25, 0.50 μM), was included as a positive control.

(C) Western blot analysis (top) and quantification (bottom) of PD-L1 protein levels in H1975 and RKO cells treated with methazolamide (MTZ; 0, 10, 20, or 40 μM) for 24 hours.

(D) Western blot analysis (top) and quantification (bottom) of PD-L1 protein levels in H1975 and RKO cells treated with acetazolamide (ATZ; 0, 10, 20, or 40 μM) for 24 hours.

(E-F) Cell viability assessed by CCK-8 assay in H1975 (E) and RKO (F) cells treated with the indicated concentrations of EZA for 24 hours.

(G) The EdU assay was used to quantitatively analyze the ratios of EdU-positive cells to assess the proliferative capacity of H1975 and RKO cells after 24 hours of treatment with various concentrations of EZA.

(H) H1975 and RKO cells were pretreated with the indicated concentrations of EZA for 24 hours and then cocultured with activated primary human T cells for 24 hours. Residual adherent tumor cells were stained with crystal violet and quantified. #P < 0.05 and ##P < 0.01 versus the corresponding tumor cell-only control (H1975 Control or RKO Control); **P < 0.01, ****P < 0.0001 versus the corresponding untreated coculture group (H1975 + T/Jurkat cells or RKO + T/Jurkat cells). Data are presented as mean ± SD from three independent experiments. Statistical significance was determined by one-way ANOVA (E-F) or two-way ANOVA (B, C, G, and H). *$P < 0.05$, **$P < 0.01$, ***$P < 0.001$, ****$P < 0.0001$; ns, not significant.

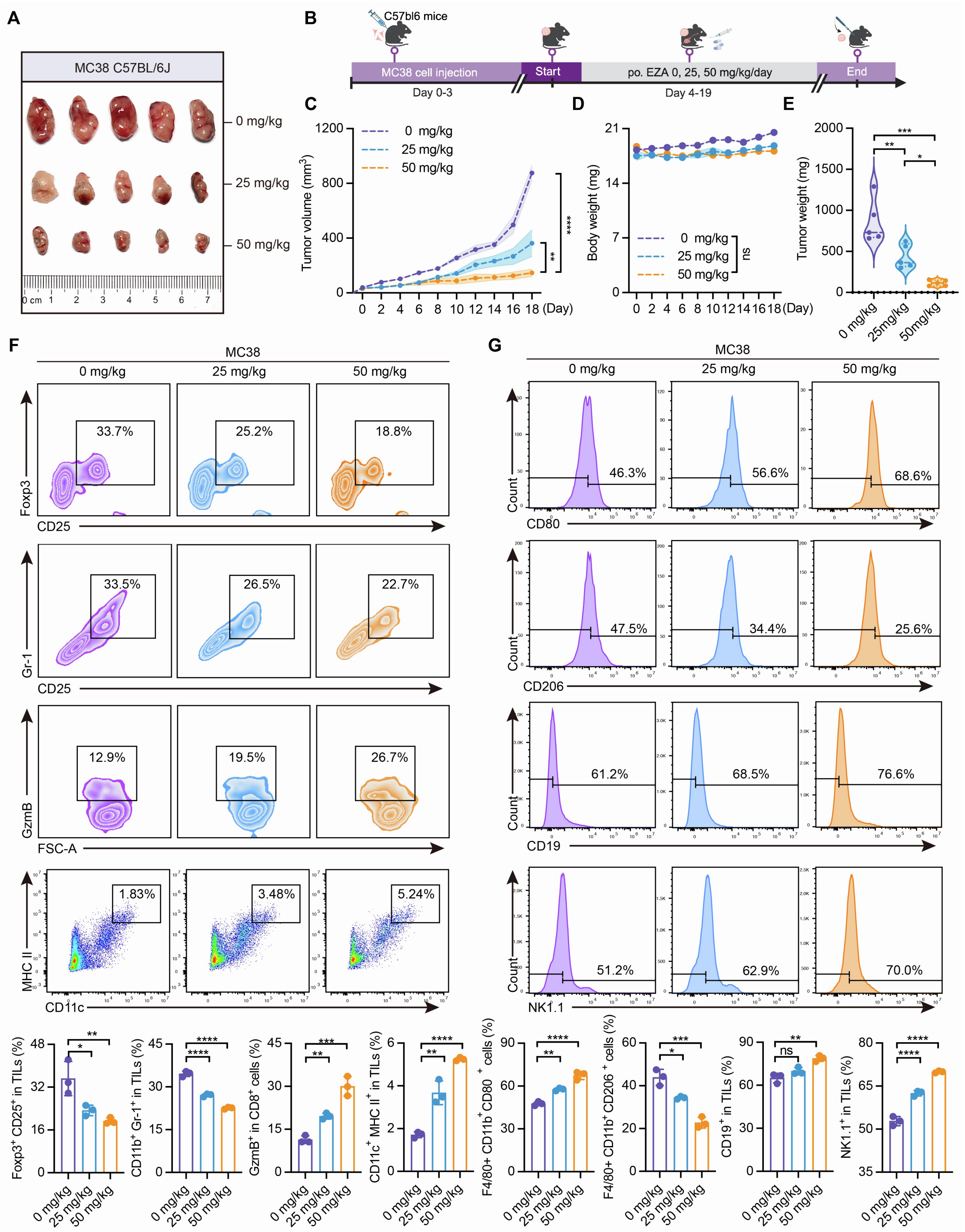

Figure S2. EZA suppresses subcutaneous MC38 colorectal tumor growth by modulating the immune microenvironment, related to Figure 2.

(A) Representative images of subcutaneous MC38 tumors from female C57BL/6J mice treated with vehicle or EZA (25 or 50 mg/kg) for the duration indicated in (B) (n = 5 mice per group).

(B) Schematic of the experimental timeline for the MC38 subcutaneous tumor model and drug administration regimen.

(C) Tumor growth curves of mice during the treatment period.

(D) Body weight changes of mice throughout the experimental timeline.

(E) Final tumor weights at the experimental endpoint.

(F-G) Flow cytometry and quantitative analysis of immune cell infiltration in the tumor microenvironment, including Tregs ($CD4^+CD25^+Foxp3^+$), Granzyme B^+ cytotoxic lymphocytes, MDSCs ($CD11b^+Gr\text{-}1^+$), mature DCs ($CD11c^+MHC\text{-}II^+$), M1 macrophages ($F4/80^+CD11b^+CD80^+$), M2 macrophages ($F4/80^+CD11b^+CD206^+$), and NK cells ($NK1.1^+$). Data in (C-G) are presented as mean ± SD. Statistical significance was determined by one-way ANOVA (E-G) or two-way ANOVA (C, D). *$P < 0.05$, **$P < 0.01$, ***$P < 0.001$, ****$P < 0.0001$; ns, not significant.

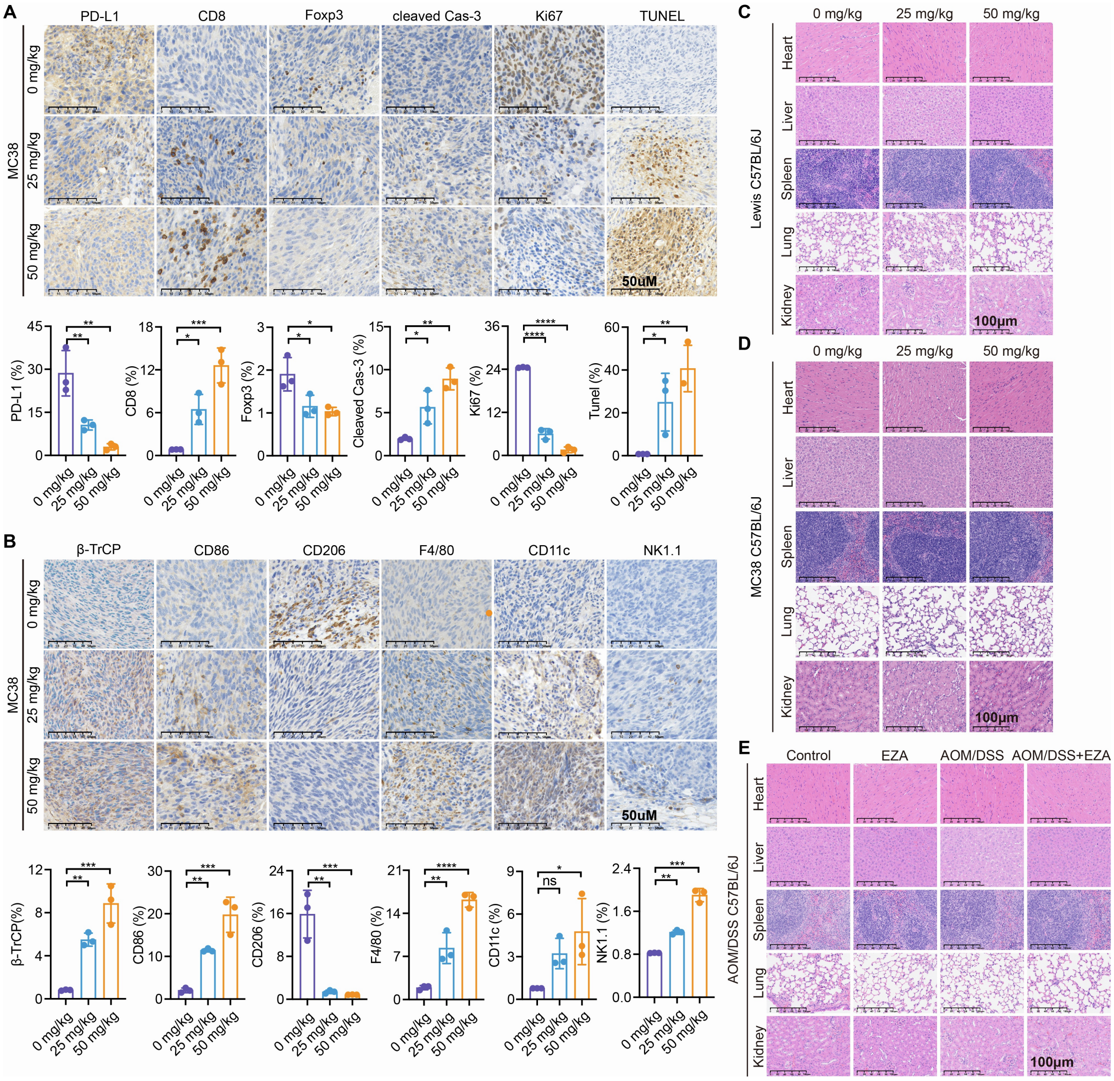

Figure S3. Analysis of tumor microenvironment remodeling and systemic safety following EZA treatment in multiple in vivo models, related to Figures 2 and 3.

(A-B) Immunohistochemical profiling of the tumor microenvironment in MC38 subcutaneous tumors. Representative images of immunohistochemical staining and quantitative analysis of tumor tissues following EZA treatment. The evaluated parameters included PD-L1, CD8, Foxp3, Caspase-3, TUNEL, Ki-67, CD86, F4/80, CD206, CD11c, and NK1.1. Scale bar, 50 μm.

(C-E) Histopathological assessment of major organ safety across multiple tumor models. Representative hematoxylin and eosin-stained sections of major organs (heart, liver, spleen, lung, and kidney) harvested from tumor-bearing mice at the experimental endpoint. (C) Subcutaneous Lewis lung carcinoma (LLC) model treated with vehicle or EZA (25 or 50 mg/kg). (D) Subcutaneous MC38 colorectal tumor model treated with vehicle or EZA (25 or 50 mg/kg). (E) AOM/DSS-induced colitis-associated colorectal cancer model treated with vehicle or EZA (50 mg/kg). No significant treatment-related pathological alterations were observed. Scale bar, 100 μm. Data are presented as mean ± SD from three independent experiments. Statistical significance was determined by one-way ANOVA (A-B)**.** Statistical significance was determined by one-way ANOVA (A-B). **P* < 0.05, ***P* < 0.01, ****P* < 0.001, *****P* < 0.0001; ns, not significant.

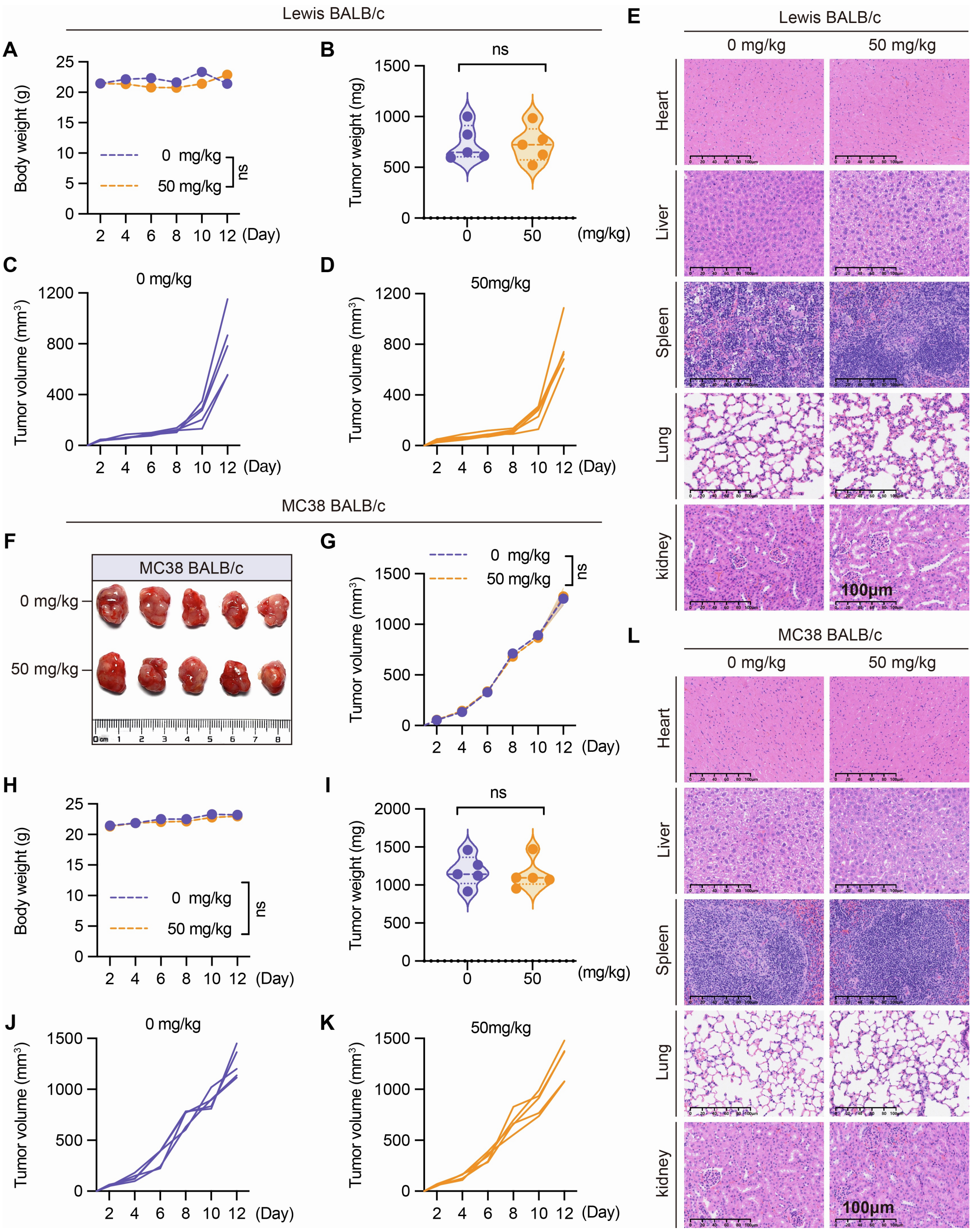

Figure S4. EZA does not inhibit lung or colorectal cancer growth in immunodeficient mouse models, related to Figures 2 and 6.

(A-D) Analysis of the LLC subcutaneous tumor model in nude mice. (A) Body weight changes of mice during the treatment period. (B) Final tumor weights at the experimental endpoint. (C-D) Individual tumor growth curves for mice treated with vehicle (C) or 50 mg/kg EZA (D).

(E) Representative H&E image of major organs from LLC-bearing nude mice at the endpoint. Scale bar, 100 μm.

(F) Representative images of excised MC38 subcutaneous tumors from nude mice treated with vehicle or 50 mg/kg EZA (n = 5 per group).

(G-I) Quantitative analysis of the MC38 model in nude mice. (G) Tumor growth curves. (H) Body weight

changes during treatment. (I) Final tumor weights.

(J-K) Individual tumor growth curves for MC38-bearing nude mice treated with vehicle (J) or 50 mg/kg EZA (K).

(L) Representative H&E-stained sections of major organs from MC38-bearing nude mice at the endpoint. Scale bar, 100 μm. Data in (A-B and G-J) are presented as mean ± SD (n = 5 mice per group). Statistical significance was determined by one-way ANOVA (B and I) or two-way ANOVA (A, G, and H). ns, not significant.

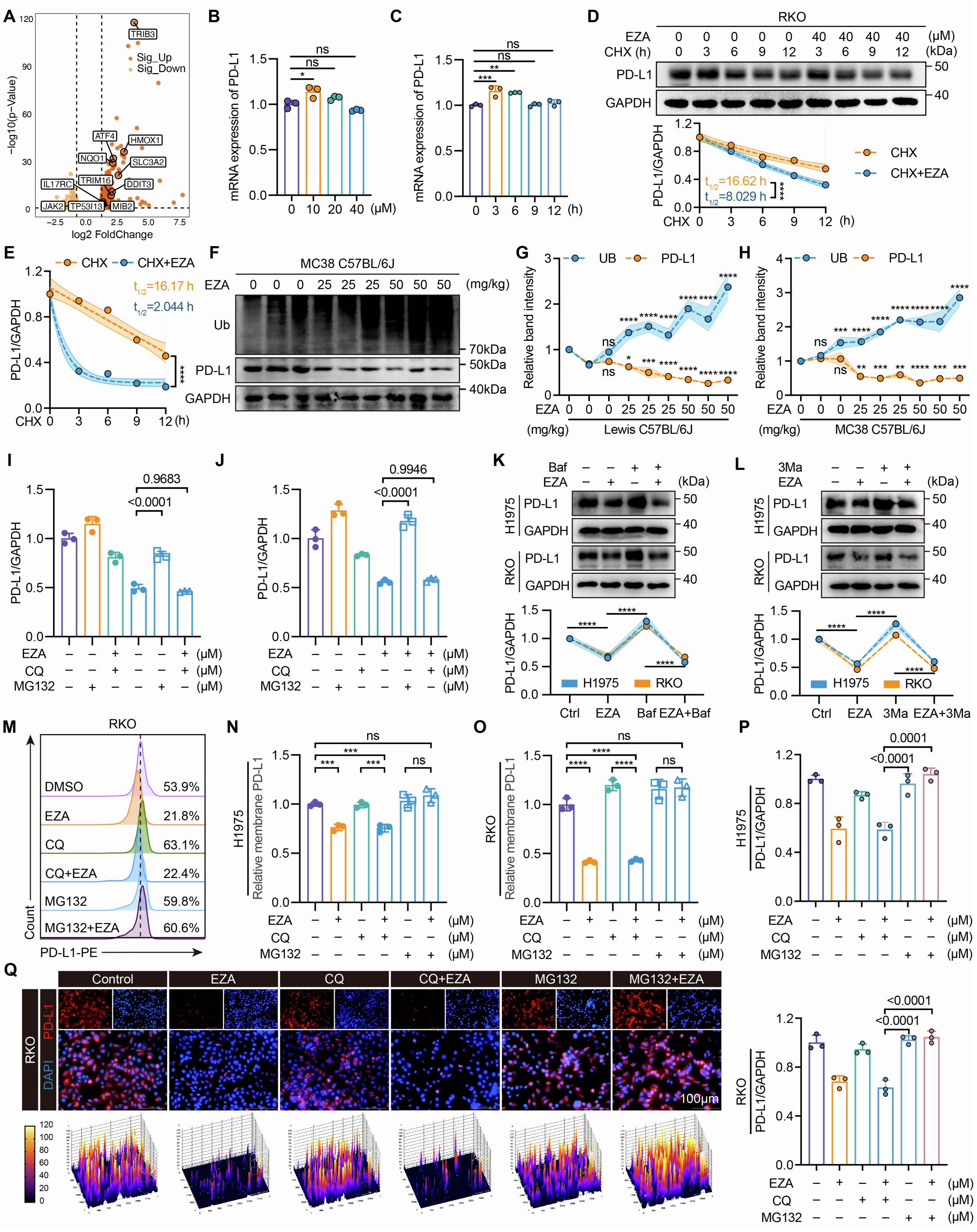

Figure S5. EZA promotes PD-L1 degradation via the ubiquitin-proteasome pathway, related to Figure 4.

(A) Volcano plot of differentially expressed genes (DEGs) in RKO cells treated with 40 μM EZA versus DMSO for 24 hours ($|\log_2$ fold change$| > 1$, $P < 0.05$). Upregulated genes are shown in red, downregulated genes in blue.

(B-C) RT qPCR analysis of PD-L1 mRNA levels in RKO cells treated with increasing concentrations of EZA for 24 hours (B) or with 40 μM EZA for the indicated durations (C). Data are normalized to GAPDH.

(D) Western blot analysis of PD-L1 protein levels in RKO cells treated with cycloheximide (CHX, 50 μg/mL) for the indicated times, with or without 40 μM EZA.

(E) Quantification of PD-L1 protein half-life from the data in (D).

(F-G) Analysis of PD-L1 ubiquitination in vivo. (F) Western blot analysis of ubiquitinated PD-L1 in MC38 subcutaneous tumors from mice treated with vehicle or EZA (25 or 50 mg/kg). (G) Corresponding quantitative analysis of the ubiquitination signal.

(H) Quantification of PD-L1 ubiquitination in Lewis lung carcinoma (LLC) subcutaneous tumors from mice treated as indicated (related to Figure 4H).

(I-J) Western blot analysis of PD-L1 protein levels in H1975 (I) and RKO (J) cells treated with EZA (40 μM, 12 h) in the presence or absence of the proteasome inhibitor MG132 (5 μM, 6 h) or the lysosome inhibitor chloroquine (CQ, 40 μM, 6 h).

(K-L) Western blot analysis (top) and quantification (bottom) of PD-L1 levels in H1975 and RKO cells co-treated with EZA and bafilomycin A1 (Baf, 800 nM, 12 h) (K) or 3-methyladenine (3MA, 1 mM, 6 h) (L).

(M) Flow cytometry analysis of cell-surface PD-L1 expression in RKO cells treated as in (I-J).

(N-O) Flow cytometry analysis of cell-surface PD-L1 expression in H1975 (N) and RKO (O) cells under the indicated treatment conditions.

(P) Quantification of the mean fluorescence intensity (MFI) of membrane PD-L1 from immunofluorescence analysis in H1975 cells (related to Figure 4G).

(Q) Representative immunofluorescence (IF) images showing membrane PD-L1 (red) in RKO cells treated as in (M). The 2.5D reconstruction (bottom) quantifies the fluorescence intensity distribution. Scale bar, 100 μm. Data are presented as mean ± SD from three independent experiments. Statistical significance was determined by one-way ANOVA (B, C, I, J, and N-Q) or two-way ANOVA (D, E, G, H, K and L). *P < 0.05, **P < 0.01, ***P < 0.001, ****P < 0.0001; ns, not significant.

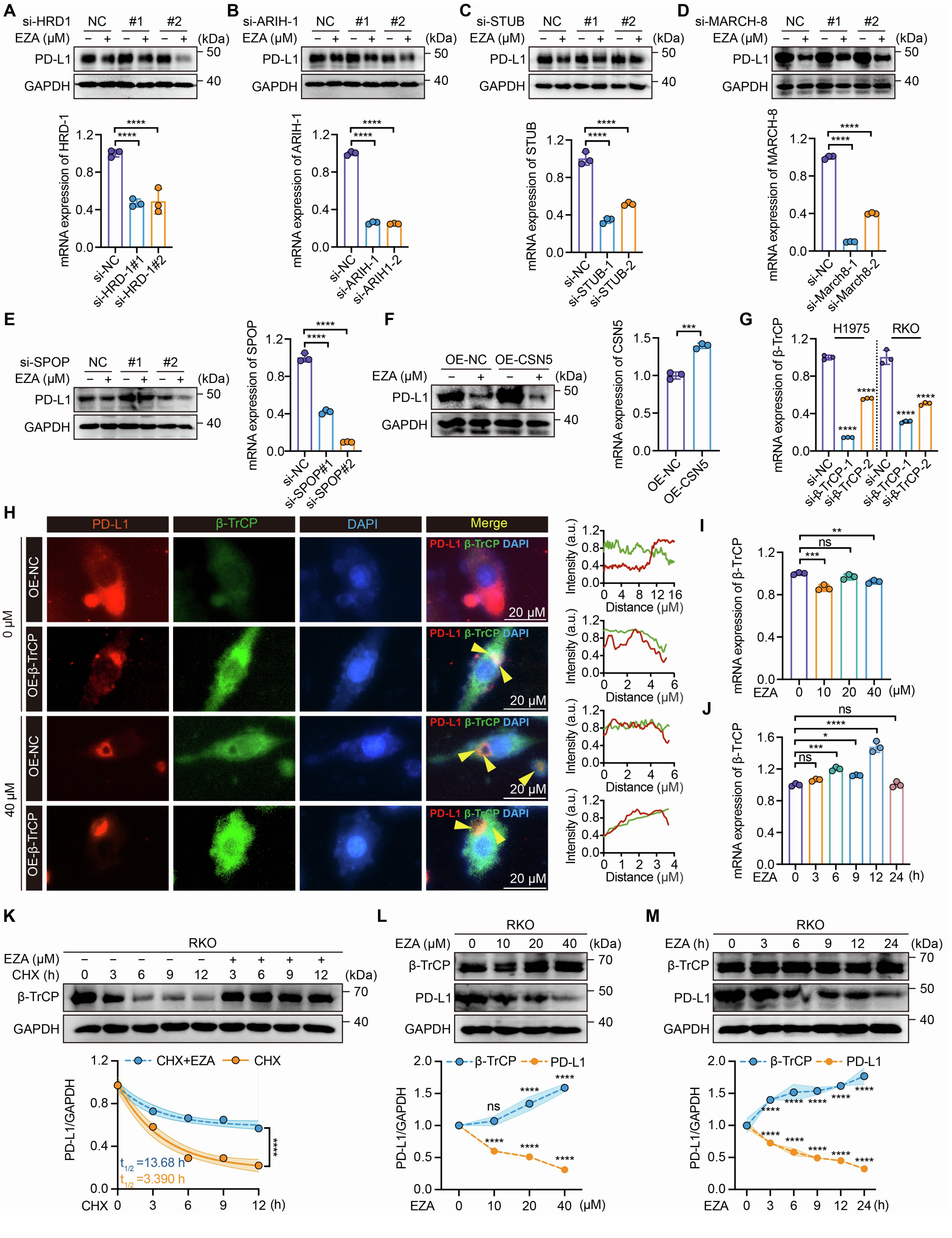

Figure S6. EZA promotes PD-L1 ubiquitination and degradation by targeting β-TrCP, related to Figure 4.

(A-F) Western blot analysis of PD-L1 protein levels in RKO cells treated with 40 μM EZA for 24 hours following knockdown or overexpression of indicated E3 ubiquitin ligases: (A) HRD1, (B) ARIH1, (C) STUB1, (D) MARCH8, (E) SPOP, and (F) CSN5. RT-qPCR confirmed the knockdown or overexpression efficiency.

(G) RT-qPCR analysis confirming the knockdown efficiency of β-TrCP in RKO cells transfected with si-β-TrCP.

(H) Representative immunofluorescence images showing the subcellular colocalization of PD-L1 (red)

and β-TrCP (green) in RKO cells transiently overexpressing β-TrCP (OE-β-TrCP) or a control vector (OE-NC), following treatment with 40 μM EZA for 6 hours. Scale bar, 20 μm.

(I-J) RT-qPCR analysis of β-TrCP mRNA expression in H1975 cells. (I) Dose-dependent effect after 24-hour treatment with the indicated concentrations of EZA. (J) Time-dependent effect after treatment with 40 μM EZA.

(K) Western blot analysis (top) and quantification (bottom) of PD-L1 protein levels in RKO cells treated with cycloheximide (CHX, 50 μg/mL) over time, with or without a 12-hour pre-treatment with 40 μM EZA.

(L-M) Western blot analysis (top) and quantification (bottom) of β-TrCP protein levels in RKO cells. (L) Dose-dependent effect after 24-hour treatment with the indicated concentrations of EZA. (M) Time-dependent effect after treatment with 40 μM EZA. Data are presented as mean ± SD from three independent experiments. Statistical significance was determined by one-way ANOVA (A-F and H-J) or two-way ANOVA (G, K, L and M). *$P < 0.05$, **$P < 0.01$, ***$P < 0.001$, ****$P < 0.0001$; ns, not significant.

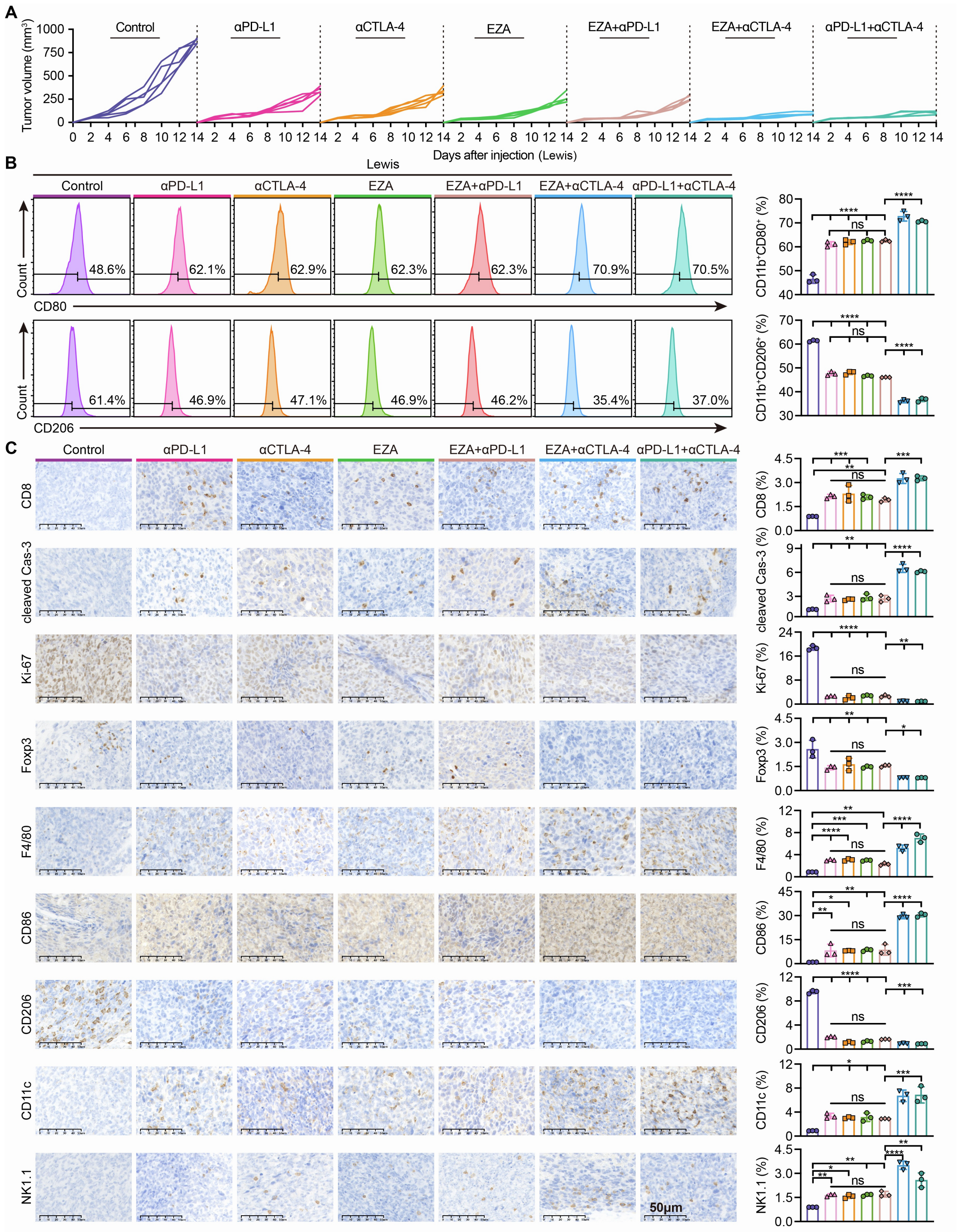

Figure S7. EZA synergizes with anti-CTLA-4 therapy to enhance antitumor immunity in the subcutaneous Lewis lung carcinoma model, related to Figure 6.

(A) Individual tumor growth curves for C57BL/6J mice bearing subcutaneous Lewis lung carcinoma (LLC) and treated with vehicle, EZA (50 mg/kg), anti-PD-L1 antibody (100 μg/mouse), anti-CTLA-4 antibody (100 μg per mouse), or their combination (n = 5 mice per group).

(B) Flow cytometric quantification of the following tumor-infiltrating macrophage subsets: M1 ($F4/80^{+}CD11b^{+}CD80^{+}$) and M2 ($F4/80^{+}CD11b^{+}CD206^{+}$).

(C) Immunohistochemical staining images and quantification of LLC tumor: CD8, Foxp3, cleaved Caspase-3, Ki-67, CD86, F4/80, CD206, CD11c, and NK1.1. Scale bar, 50 μm. Data are presented as

mean ± SD. Statistical significance was determined by one-way ANOVA (B, C). *$P < 0.05$, **$P < 0.01$, ***$P < 0.001$, ****$P < 0.0001$; ns, not significant.

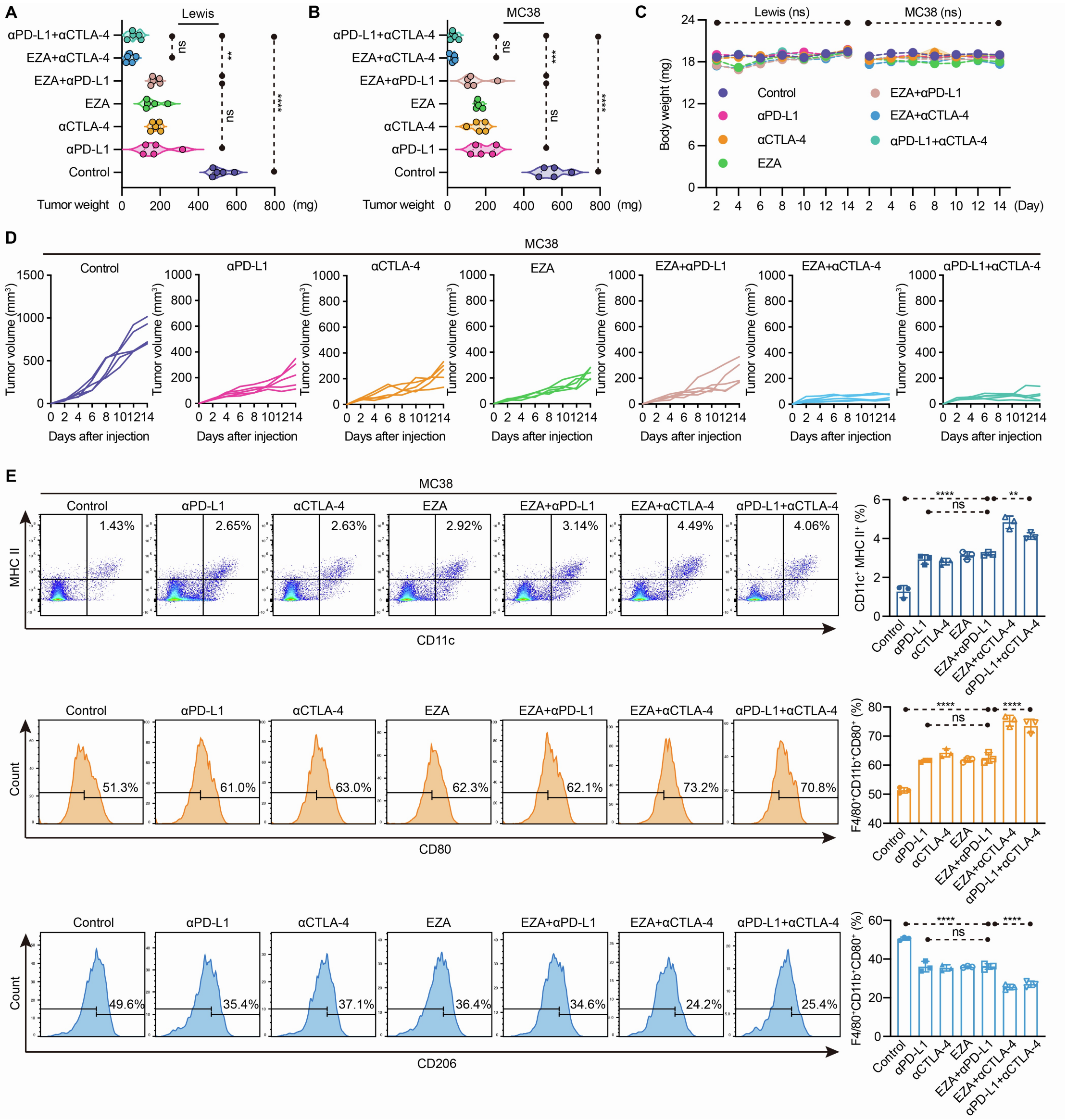

Figure S8. EZA synergizes with anti-CTLA-4 therapy to enhance antitumor immunity in the MC38 colorectal cancer subcutaneous model, related to Figure 6. (A) Schematic of the experimental design for drug treatment in MC38 tumor-bearing mice. Female C57BL/6J mice were treated with vehicle, EZA (50 mg/kg), anti-PD-L1 antibody (100 µg/mouse), anti-CTLA-4 antibody (100 µg/mouse), or the indicated combination (n = 5 mice per group).

(B) Body weight changes of mice during the treatment period.

(C) Final tumor weights at the experimental endpoint.

(D) Individual tumor growth curves for each mouse in the treatment groups described in (A). Tumor volume was measured twice weekly.

(E) Flow cytometric analysis of immune cell subsets within the tumor immune microenvironment. Quantified populations include mature dendritic cells ($CD11c^{+}MHC\text{-}II^{+}$), M1 macrophages ($F4/80^{+}CD11b^{+}CD86^{+}$), and M2 macrophages ($F4/80^{+}CD11b^{+}CD206^{+}$). Data in (B), (C), (D), and (E) are presented as mean ± SD. Statistical significance was determined by two-way ANOVA (B, D) or by one-way ANOVA (C, E). *$P < 0.05$, **$P < 0.01$, ***$P < 0.001$, ****$P < 0.0001$; ns, not significant.

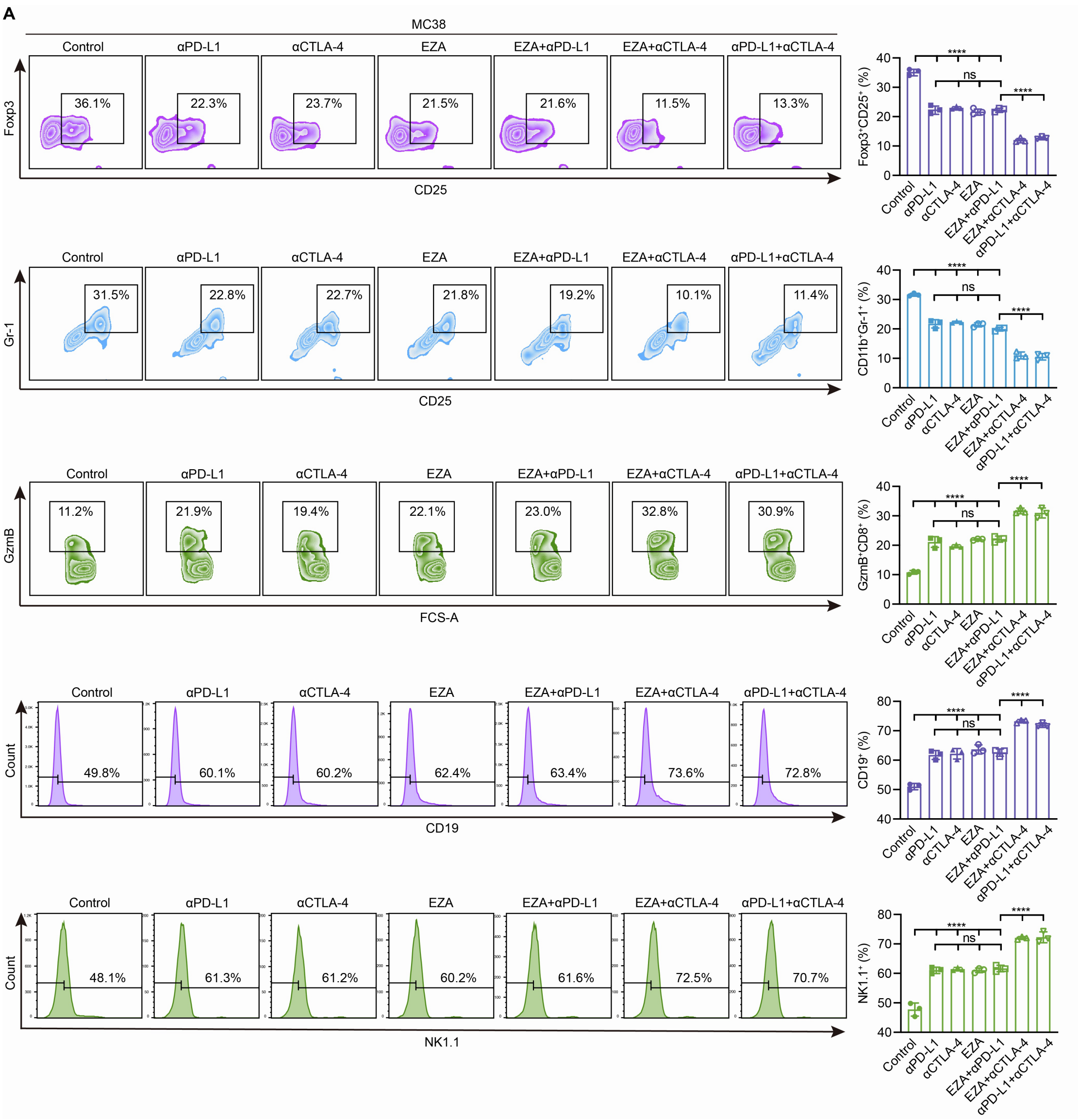

Figure S9. Flow cytometric analysis of immune cell modulation by the EZA/anti-CTLA-4 combination in the subcutaneous MC38 colorectal cancer model, related to Figure 6.

(A) Quantification of immune cell subsets within the tumor immune microenvironment of MC38 tumors from mice treated with vehicle, EZA (50 mg/kg), anti-PD-L1 antibody (100 μg/mouse), anti-CTLA-4 antibody (100 μg per mouse), or their combination. Quantified populations include Tregs ($CD4^+CD25^+Foxp3^+$), MDSCs ($CD11b^+Gr\text{-}1^+$), Granzyme B^+ cytotoxic lymphocytes, B cells ($CD19^+$), NK cells ($NK1.1^+$), and M1 macrophages ($F4/80^+CD11b^+CD86^+$). Data are presented as mean ± SD. Statistical significance was determined by one-way ANOVA (A). *$P < 0.05$, **$P < 0.01$, ***$P < 0.001$, ****$P < 0.0001$; ns, not significant.

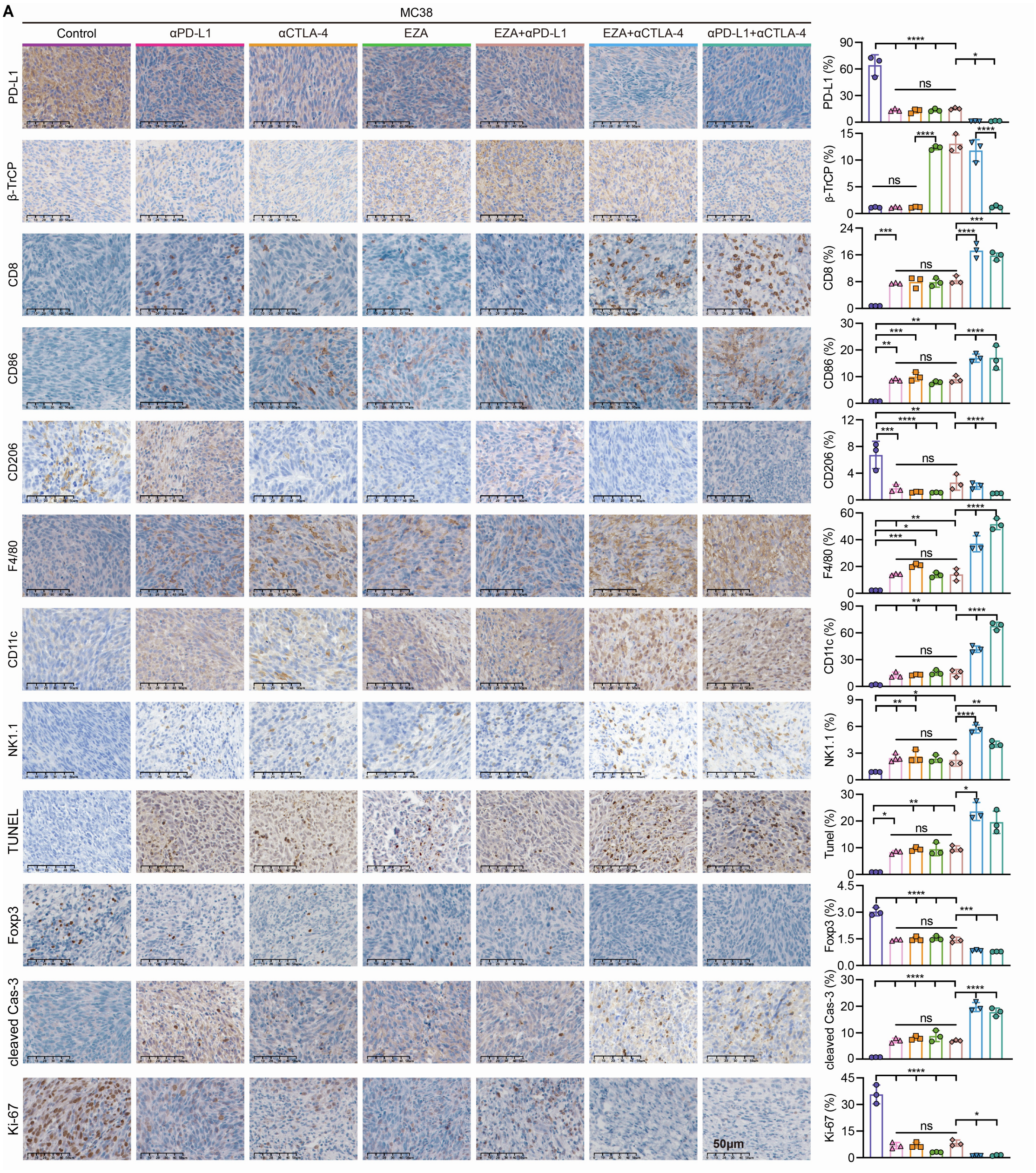

Figure S10. Immunohistochemical analysis of immune cell modulation by the EZA/anti-CTLA-4 combination in the subcutaneous MC38 colorectal cancer model, related to Figure 6.

(A) Immunohistochemical and quantitative analysis of the MC38 tumor microenvironment from mice treated with vehicle, EZA (50 mg/kg), anti-PD-L1 antibody (100 µg/mouse), anti-CTLA-4 antibody (100 µg/mouse), or their combination: PD-L1, β-TrCP, CD8, CD86, F4/80, CD206, CD11c, NK1.1, TUNEL, cleaved caspase-3, Ki-67, and Foxp3+ Tregs. scale bar: 50 µm. Data in (A) are presented as mean ± SD. Statistical significance was determined by one-way ANOVA (A). $^{*}P < 0.05$, $^{**}P < 0.01$, $^{***}P < 0.001$, $^{****}P < 0.0001$; ns, not significant.

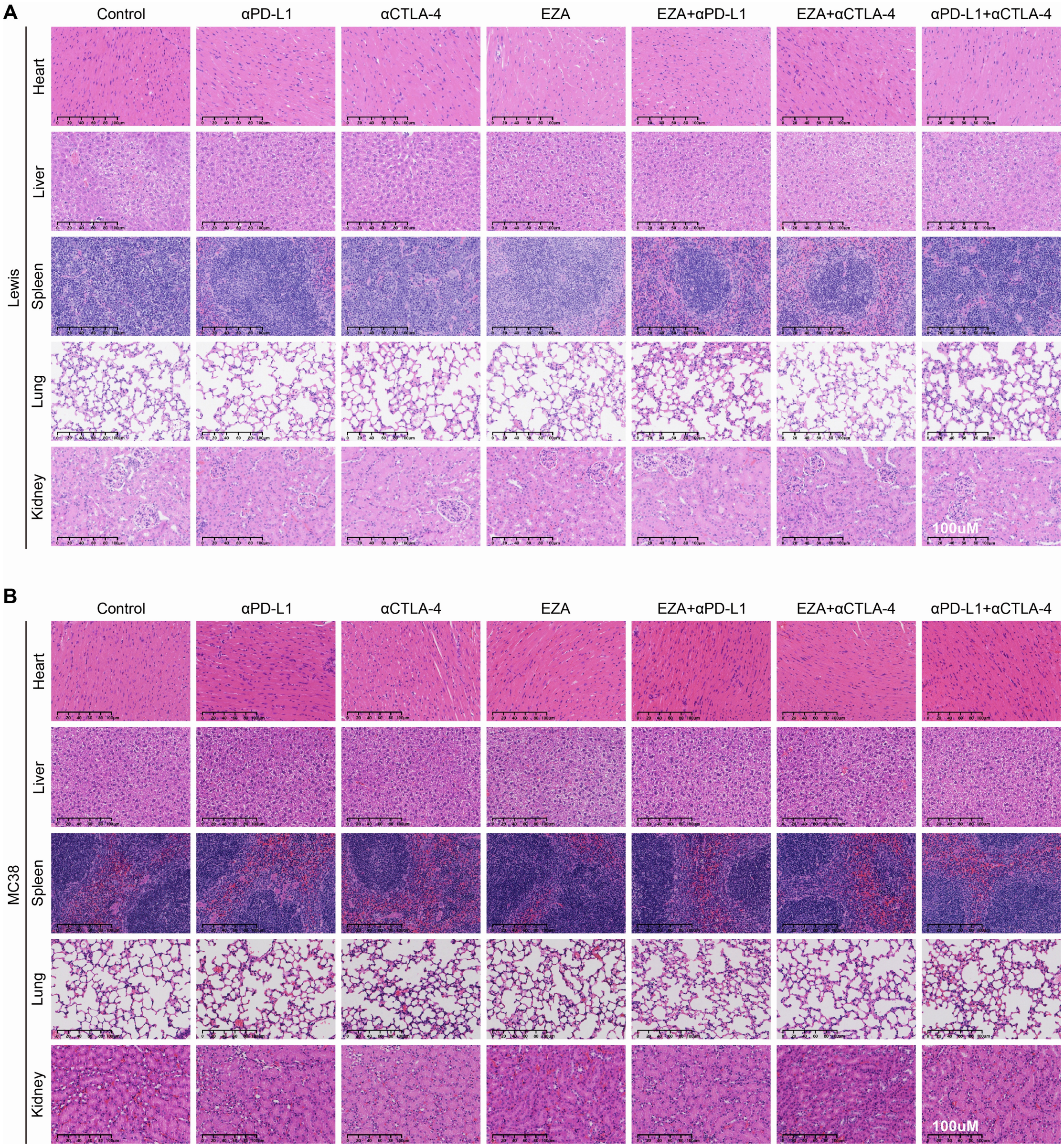

Figure S11. Safety evaluation of EZA and its combination therapy in subcutaneous tumor models, related to Figures 6.

(A-B) Histopathological assessment of major organs by H&E staining in: (A) subcutaneous Lewis lung carcinoma model; (B) subcutaneous MC38 colorectal cancer model (scale bar: 100 μm). No significant treatment-related pathological alterations were observed in any examined organ. Scale bar, 100 μm.

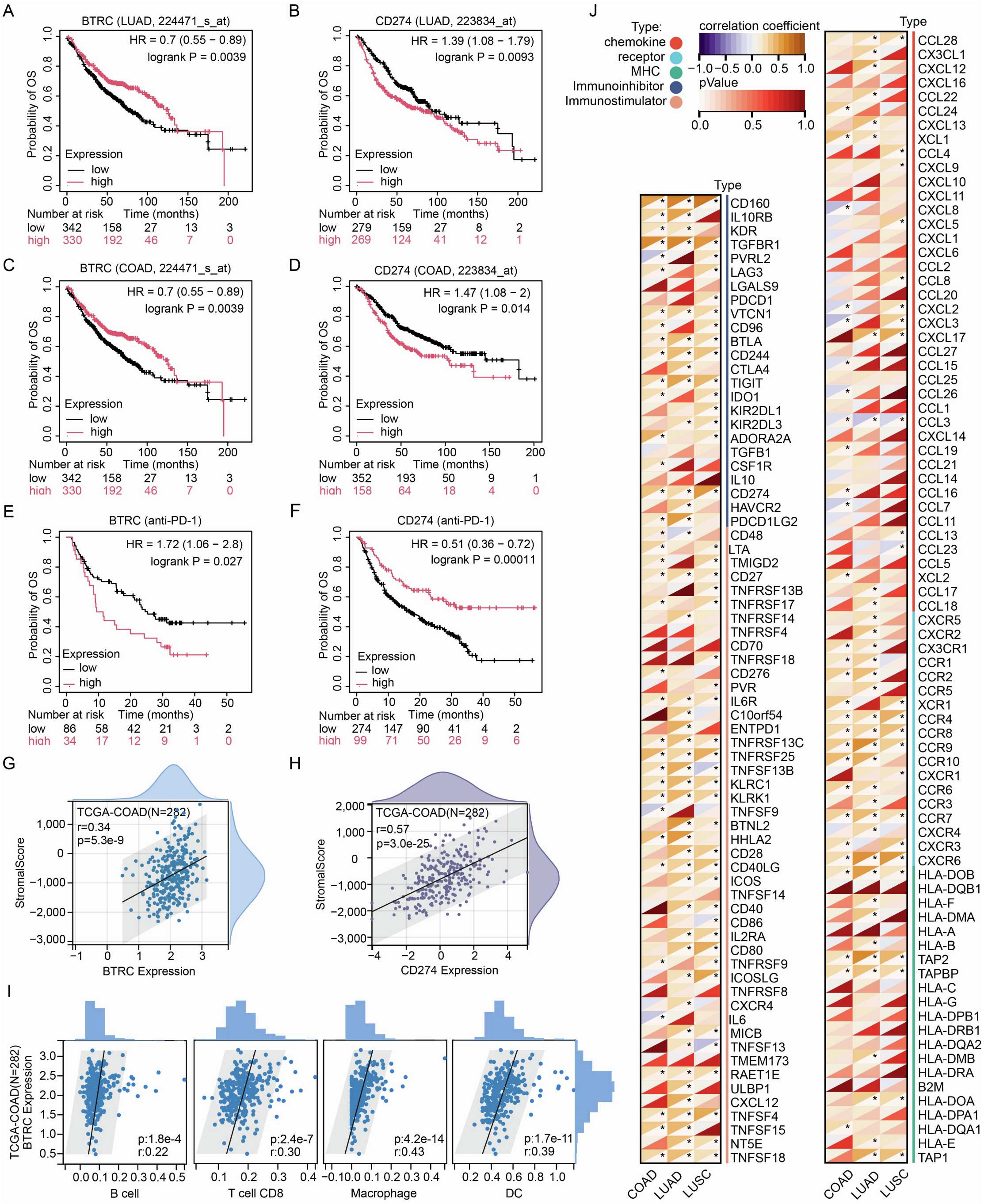

Figure S12. Prognostic and immunomodulatory significance of β-TrCP (BTRC) and PD-L1 (CD274) across cancer types, related to Figures 7.

(A-D) Kaplan-Meier overall survival (OS) analysis in the TCGA cohorts. Patients with lung adenocarcinoma (LUAD, A-B) and colon adenocarcinoma (COAD, C-D) were stratified by high or low expression of BTRC (A, C) or CD274 (B, D). *P* values were calculated using the log-rank test.

(E-F) Correlation between OS and BTRC (E) or CD274 (F) expression in a pancancer cohort of patients treated with anti-PD-1 therapy.

(G-H) Stromal scores estimated by the ESTIMATE algorithm in the TCGA-COAD cohort, plotted against expression levels of BTRC (G) and CD274 (H).

(I) Spearman correlation analysis between BTRC expression and the infiltration levels of selected immune cell types (B cells, CD8^{+} T cells, macrophages, dendritic cells) in the TCGA-COAD cohort.

(J) Heatmap of Pearson correlation coefficients between BTRC expression and 150 immune-related genes across TCGA-COAD, LUAD, and lung squamous cell carcinoma (LUSC) cohorts. Genes are grouped into five functional categories: chemokines (n=41), receptors (n=18), MHC molecules (n=21), immunoinhibitors (n=24), and immunostimulators (n=46). Survival differences in (A-F) were assessed using the log-rank test. Correlations in (G-I) were evaluated by Spearman's rank correlation, while the correlation matrix in (J) was generated using Pearson correlation. *$P < 0.05$.

Supplementary tables

Table S1. Baseline Demographic, Clinical, and Pathologic Characteristics of NSCLC Patients Treated with Neoadjuvant Immunochemotherapy, related to Figure 7

Variable	Overall N = 32[1]	Non-Responder N = 16[1]	Responder N = 16[1]	*P*-value[2]
**Response**				<0.001
pCR[3]	10 (31%)	0 (0%)	10 (63%)	
MPR[4]	6 (19%)	0 (0%)	6 (38%)	
Non-MPR	16 (50%)	16 (100%)	0 (0%)	
**Sex**				>0.9
Female	5 (16%)	3 (19%)	2 (13%)	
Male	27 (84%)	13 (81%)	14 (88%)	
**Age**				0.3
<65	19 (59%)	8 (50%)	11 (69%)	
≥65	13 (41%)	8 (50%)	5 (31%)	
**Histology**				0.015
LUAD[5]	9 (28%)	8 (50%)	1 (6.3%)	
LUSC[6]	23 (72%)	8 (50%)	15 (94%)	
**T**				>0.9
1b	1 (3.1%)	0 (0%)	1 (6.3%)	
1c	1 (3.1%)	1 (6.3%)	0 (0%)	
2a	4 (13%)	2 (13%)	2 (13%)	
2b	7 (22%)	4 (25%)	3 (19%)	
3	10 (31%)	5 (31%)	5 (31%)	
4	9 (28%)	4 (25%)	5 (31%)	
**N**				0.3
0	6 (19%)	4 (25%)	2 (13%)	
1	5 (16%)	1 (6.3%)	4 (25%)	
2	20 (63%)	11 (69%)	9 (56%)	
3	1 (3.1%)	0 (0%)	1 (6.3%)	
**cTNM stage**				0.7
IB	2 (6.3%)	1 (6.3%)	1 (6.3%)	

Variable	Overall<br>N = 32[1]	Non-Responder<br>N = 16[1]	Responder<br>N = 16[1]	*P*-value[2]
IIA	1 (3.1%)	1 (6.3%)	0 (0%)	
IIB	6 (19%)	4 (25%)	2 (13%)	
IIIA	9 (28%)	3 (19%)	6 (38%)	
IIIB	13 (41%)	7 (44%)	6 (38%)	
IIIC	1 (3.1%)	0 (0%)	1 (6.3%)	
**TRG**[7]				<0.001
0	10 (31%)	0 (0%)	10 (63%)	
1	6 (19%)	0 (0%)	6 (38%)	
2	14 (44%)	14 (88%)	0 (0%)	
3	2 (6.3%)	2 (13%)	0 (0%)	
**PS**[8]				0.7
≥ 1	11 (34%)	6 (38%)	5 (31%)	
< 1	21 (66%)	10 (62%)	11 (69%)	
**Adverse effect**				0.4
0	22 (69%)	9 (56%)	13 (81%)	
1	6 (19%)	4 (25%)	2 (13%)	
2	4 (13%)	3 (19%)	1 (6.3%)	
**Change in CT value (ΔCT)**[9]	0.35<br>(0.17, 0.54)	0.22<br>(0.12, 0.36)	0.44<br>(0.27, 0.82)	0.024
**PFS (month)**[10]	27.6 ± 16.8	18.9 ± 14.2	36.4 ± 13.8	0.036
**OS (month)**[11]	29.4 ± 14.8	22.5 ± 13.1	36.2 ± 13.5	0.022

[1]n (%); Median (Q1, Q3); [2]Fisher's exact test; Pearson's Chi-squared test; Wilcoxon rank sum test; [3]pCR, pathological complete response; [4]MPR, major pathologic response;[5]LUAD, Lung Adenocarcinoma;[6]LUSC, Lung Squamous Cell Carcinoma; [7]TRG, Tumor Regression Grade; [8]PS, performance status; [9]ΔCT, Ratio of Post-treatment to Pre-treatment Maximum Tumor Diameter on Computed Tomography; [10]PFS, Progression-Free Survival; [11]OS, Overall Survival.

Table S2. Sequences of siRNAs and PCR Primers, related to STAR Methods

siRNA/PCR Primer	Sequences (5′→3′)
si-β-TrCP-1	GCGUUGUAUUCGAUUUGAUAA
si-β-TrCP-2	GCUGAACUUGUGUGCAAGGAA
si-PD-L1-1	TCAATTGTCATATTGCTAC
si-PD-L1-2	TTGACTCCATCTTTCTTCA
si-HRD1-1	UGUCUGGCCUUCACCGUUU
si-HRD1-2	CCAAGAGACUGCCCUGCAA
si-ARIH1-1	CGAGAUAUUUCCCAAGAUUUU
si-ARIH1-2	CCAUGUUGUUAAAGUCCAAUA
si-SPOP-1	CACAAGGCUAUCUUAGCAGCU
si-SPOP-2	CUCCUACAUGUGGACCAUCAA
si-MARCH8-1	GGACATTTCATGAGTCATT
si-MARCH8-2	GGAAGAGACTCAAGGCCTA
si-STUB1-1	GCAGUCUGUGAAGGCGCACUU
si-STUB1-2	CCCAAGUUCUGCUGUUGGACU
PD-L1 *F*	GGCATTTGCTGAACGCAT
PD-L1 *R*	CAATTAGTGCAGCCAGGT
T180A PD-L1 *F*	GGTAAGACCgCCACCACCAATTTCAAG
T180A PD-L1 *R*	ATTGGTGGTGGcGGTCTTACCACTCAGG
S184A PD-L1 *F*	ACCACCAATgCCAAGAGAGAGGAGAAGCTTTTC
S184A PD-L1 *R*	TCCTCTCTCTTGGcATTGGTGGTGGTGGTCTTA
β-TrCP *F*	TGGCTCATCTGACAACACTATC
β-TrCP *R*	CGAATACAACGCACCAATTCC
β-actin *F*	ATTCCTATGTGGGCGACGAG
β-actin *R*	CCAGATTTTCTCCATGTCGTCC
HRD1 *F*	TGCGTAACATCCACACACTG
HRD1 *R*	CTTTGAGTTTGTATCTTGGATGCC
ARIH1 *F*	GCATCTTCAGGTAGCACAAGGC

ARIH1 *R*	ACTTTGATGGAAACCTGGAGAA
SPOP *F*	GCCAGTGAAATACGAGTTAGGG
SPOP *R*	CCTGGAGCGCTTAAAGGTCA
STUB1 *F*	CGAATACAACGCACCAATTCC
STUB1 *R*	TCAAGGAGCAGGGCAATCGTCT
MARCH8 *F*	AGTGACATTCCACGTCATTGC
MARCH8 *R*	GATCTCCTCAGCAGTACGGTC
